# Accessibility and contribution to glucan masking of natural and genetically tagged versions of yeast wall protein 1 of *Candida albicans*

**DOI:** 10.1371/journal.pone.0191194

**Published:** 2018-01-12

**Authors:** Bruce L. Granger

**Affiliations:** Department of Microbiology and Immunology, Montana State University, Bozeman, Montana, United States of America; Institute of Biology Valrose, FRANCE

## Abstract

Yeast wall protein 1 (Ywp1) is an abundant glycoprotein of the cell wall of the yeast form of *Candida albicans*, the most prevalent fungal pathogen of humans. Antibodies that bind to the polypeptide backbone of isolated Ywp1 show little binding to intact yeast cells, presumably because the Ywp1 epitopes are masked by the polysaccharides of the mannoproteins that form the outer layer of the cell wall. Rare cells do exhibit much greater anti-Ywp1 binding, however, and one of these was isolated and characterized. No differences were seen in its Ywp1, but it exhibited greater adhesiveness, sensitivity to wall perturbing agents, and exposure of its underlying β-1,3-glucan layer to external antibodies. The molecular basis for this greater epitope accessibility has not been determined, but has facilitated exploration of how these properties change as a function of cell growth and morphology. In addition, previously engineered strains with reduced quantities of Ywp1 in their cell walls were also found to have greater β-1,3-glucan exposure, indicating that Ywp1 itself contributes to the masking of wall epitopes, which may be important for understanding the anti-adhesive effect of Ywp1. Ectopic production of Ywp1 by hyphae, which reduces the adhesivity of these filamentous forms of *C*. *albicans*, was similarly found to reduce exposure of the β-1,3-glucan in their walls. To monitor Ywp1 in the cell wall irrespective of its accessibility, green fluorescent protein (Gfp) was genetically inserted into wall-anchored Ywp1 using a bifunctional cassette that also allowed production from a single transfection of a soluble, anchor-free version. The wall-anchored Ywp1-Gfp-Ywp1 accumulated in the wall of the yeast forms but not hyphae, and appeared to have properties similar to native Ywp1, including its adhesion-inhibiting effect. Some pseudohyphal walls also detectably accumulated this probe. Strains of *C*. *albicans* with tandem hemagglutinin (HA) epitopes inserted into wall-anchored Ywp1 were previously created by others, and were further explored here. As above, rare cells with much greater accessibility of the HA epitopes were isolated, and also found to exhibit greater exposure of Ywp1 and β-1,3-glucan. The placement of the HA cassette inhibited the normal N-glycosylation and propeptide cleavage of Ywp1, but the wall-anchored Ywp1-HA-Ywp1 still accumulated in the cell wall of yeast forms. Bifunctional transformation cassettes were used to additionally tag these molecules with Gfp, generating soluble Ywp1-HA-Gfp and wall-anchored Ywp1-HA-Gfp-Ywp1 molecules. The former revealed unexpected electrophoretic properties caused by the HA insertion, while the latter further highlighted differences between the *presence* of a tagged Ywp1 molecule (as revealed by Gfp fluorescence) and its *accessibility* in the cell wall to externally applied antibodies specific for HA, Gfp and Ywp1, with accessibility being greatest in the rapidly expanding walls of budding daughter cells. These strains and results increase our understanding of cell wall properties and how *C*. *albicans* masks itself from recognition by the human immune system.

## Introduction

The most prevalent fungal pathogen of humans is *Candida albicans*. It colonizes the skin, alimentary tract and other mucosal surfaces of most of us as a benign commensal, but can cause significant morbidity or mortality when our immune defenses are diminished [1, 2]. This stealthy opportunism relies in part on the ability of *C*. *albicans* to remain unrecognized by elements of the innate and adaptive immune system [3]. The innate immune system normally responds rapidly to molecular patterns common to pathogens but absent from the host, while the adaptive immune system can respond more slowly and specifically to nearly any foreign antigen. For *C*. *albicans*, the primary point of contact and recognition is the cell wall, a dynamic and pliable exoskeleton that surrounds each cell, with inner layers rich in polymers of glucose and outer layers rich in mannosylated glycoproteins [4]. Cells of the innate immune system are capable of immediate responses to distinctive polysaccharides present at the surface and at various depths within the cell wall [3, 5, 6]; in addition, some surface carbohydrate and polypeptide epitopes can elicit protective antibody and cellular responses by the adaptive immune system, and have shown efficacy in vaccine formulations [7–12]. None of these molecules can elicit a protective response if they are hidden from the immune system, and indeed some of them, such as the β-1,3-glucan that comprises the bulk of the cell wall structure, are largely masked by more innocuous molecules that physically overlie them [5, 13–15].

Each cell makes its own wall as it grows. Most of the proteins of the cell wall first arrive at the surface of the cell linked by their trimmed C-termini to glycosylphosphatidylinositol (GPI), a lipid with its acyl chains anchored in the plasma membrane. Subsequent cleavage of the glycosyl moiety allows transfer of that end of the protein to the glucan network, where it is covalently attached to chains of β-1,6-glucan, themselves linked to the strands of β-1,3-glucan. These GPI proteins have typically been highly decorated with mannose-rich glycans during synthesis, and as such collectively form a shielding layer of mannoproteins at the surface of the assembled wall [16, 17]. The cells themselves are polymorphic, with distinct ovoid (yeast form) and elongated (filamentous hyphal and pseudohyphal) unicellular and multicellular forms depending on environmental conditions and demands [18–20]. One of the most abundant GPI proteins of the wall of yeast forms (blastoconidia) is yeast wall protein 1 (Ywp1) [21–27], which is absent from hyphae (reviewed in [21]). During synthesis, this 533 amino acid (aa) polypeptide loses its N-terminal 21 aa as it translocates into the secretory pathway, and its C-terminal 22 aa as it acquires its temporary GPI anchor; cleavage at tribasic and dibasic sites creates a propeptide of about 100 aa that remains firmly associated with the 378 aa core, which becomes anchored to the wall glucan; the core is heavily O-mannosylated, and the associated propeptide harbors the protein’s single N-glycan [21, 22]. The only known consequence of the loss of Ywp1 or its wall anchorage is greater cellular adhesiveness; ectopic production of Ywp1 diminishes the adhesivity of hyphae, suggesting an antiadhesive effect for wall-anchored Ywp1 [21, 22], although the molecular mechanism remains unknown. Yeast that are starved for phosphate increase their production of Ywp1, perhaps as a mechanism for passive relocation to a better nutrient source, even though other forms of starvation do not similarly increase Ywp1 [21, 22]. Antibodies specific for the Ywp1 polypeptide, created by DNA vaccination (genetic immunization) of mice, exhibit little if any binding to intact yeast cells that have abundant Ywp1 in their cell walls, presumably because the epitopes are shielded by the outer mannoprotein layer or other component(s) of the cell wall [22]. Insertion of tandem HA epitopes into Ywp1, however, has allowed visualization of binding of anti-HA antibodies to the yeast surface [28].

The expression of *YWP1* in yeast cells has been visualized and quantified using the cytosolic reporters green fluorescent protein (Gfp) [21], red fluorescent protein [29] and click beetle luciferase [30], and some of the biochemical properties of Ywp1 have been elucidated using truncated, secreted versions tagged with Gfp at their C-termini [21], but visualization of Ywp1 in the cell wall has not been achieved without the use of external probes (antibodies) that may have trouble quantitatively accessing their cognate epitopes [28]. Here, genetic insertion of *GFP* into *YWP1* has successfully generated wall-anchored Ywp1-Gfp-Ywp1 that allows full visualization of Ywp1 in the cell wall, even when shielded from external probes. In addition, strain variants with diminished shielding have been isolated and found to exhibit greater accessibility to antibodies that bind to Ywp1 and β-1,3-glucan epitopes, and Ywp1 itself has been found to contribute to the masking of glucan. Although the molecular architecture of the fungal cell wall has been described in general terms, details of the biophysical properties and spatial arrangements of specific molecules remain largely uncertain; this work sheds light on these relationships in the context of an abundant mannoprotein (Ywp1) and the glucan matrix to which it is linked, in variant and wild type cells, under a variety of growth conditions. These results and tools may lead to a better understanding of the ways in which *C*. *albicans* shields itself from immune recognition.

## Materials and methods

### Strains

Strains of *Candida albicans* used here include clinical isolate SC5314 [31], auxotrophic (*Δarg4 Δhis1 Δura3*) derivative BWP17 [32], restored prototroph DAY185 [33], BWP17 with *YWP1* disrupted and/or restored (strains 3L1, 4L1, 7r1, 16r1, 16r2, 16s1 and 16s2) [21, 22], BWP17 with the coding sequence of one or both alleles of *YWP1* replaced with the coding sequence of *GFP* (strains BJ4eS8 and BJ4eS2, respectively, which are independent versions of BJ3 and BJ3a1a [22]), BWP17 derivatives (strains HY13aΔU1ΔYB and HY16bΔU2ΔYB) that had both native *YWP1* alleles disrupted and the coding sequence of one allele of *HWP1* replaced with the coding sequence of *YWP1* to allow ectopic, hyphal production of Ywp1, paired with negative controls that had the ectopic *YWP1* subsequently disrupted (strains HY13aΔU1ΔYA and HY16bΔU2ΔYC) [21], and *YWP1-HA* strains M1793 and M1796 (M1796 is also *Δsap9 Δsap10*) [28]. Strains further characterized or modified for this report are described in Table 1.

**Table 1:** *Candida albicans* strain abbreviations and descriptions.

Strain designation	Brief description (critical features in bold)	Origin strain	Laboratory name(s)
**BWP17c**	Subclones of BWP17 that exhibit **typical exposure of Ywp1 epitopes**	BWP17	• BWP17XYC3 • BWP17XYC6
**BWP17x**	Subclones of BWP17 that exhibit **greater exposure of Ywp1 epitopes**	BWP17	• BWP17XY20 • BWP17XY44 • BWP17XY52 • BWP17XY107
**YG**	*YWP1-6HA-YWP1 / YWP1-GFP-URA3-YWP1*; wall-anchored Ywp1-6HA-Ywp1; **secretes Ywp1-Gfp**	ETY3	3F3
	*YWP1-6HA-YWP1 / YWP1-GFP-URA3-YWP1*; wall-anchored Ywp1-6HA-Ywp1; **secretes Ywp1(aa1-520)-Gfp**		3G2
	*YWP1 / YWP1-GFP-URA3-GFP-YWP1*; wall-anchored Ywp1; **secretes Ywp1-Gfp**	BWP17	• βWT1a
**YGY**	*YWP1 / YWP1-GFP-YWP1*; **wall-anchored** Ywp1 and **Ywp1-Gfp-Ywp1**	• βWT1a	• βWT1aΔU1 • βWT1aΔU1a
**YGY-Y**	**Wall-anchored Ywp1-Gfp-Ywp1; no wild type Ywp1**	βWT1aΔU1	βWT1aΔU1ΔY3
**YGY-G**	*YWP1-GFP-YWP1* disrupted; **has only wild type Ywp1**		βWT1aΔU1ΔY5
**YGY+U**	**YGY** with *URA3* inserted outside of *YWP1* locus		βWT1aΔU1ΔY7
**YGY-Y’**	**Subclone of YGY-Y**	βWT1aΔU1ΔY3	βWT1aΔU1ΔY3a6
**YGY-Y-G**	**Subclone of YGY-Y that lost its Gfp**	βWT1aΔU1ΔY3	βWT1aΔU1ΔY3a2
YGY*	*YWP1 / YWP1-GFP-YWP1*; **increased quantity of wall-anchored Ywp1-Gfp-Ywp1 observed**	βWT1aΔU1	• βWT1aΔU1X1 • βWT1aΔU1X1a
**YHY**	*YWP1 / YWP1-6HA-YWP1*; **wall anchored** Ywp1 and **Ywp1-6HA-Ywp1**	M1793	• ETY1 • ETY3 • ETY3e • ETY3eL • ETY3e5 • ETY3e7 • ETY3eS (mix)
**YHYx**	*YWP1-6HA-YWP1 / YWP1-6HA-YWP1*; wall-anchored Ywp1-6HA-Ywp1; **increased exposure of HA epitopes**	ETY3e	• ETY3e13 • ETY3e13t • ETY3e13tt • ETY3e14 • ETY3e17
**YHG**	*YWP1 / YWP1-6HA-GFP-URA3-YWP1*; wall-anchored Ywp1; **secretes Ywp1-6HA-Gfp**	ETY1	2F4
		ETY3	3F2
	*YWP1 / YWP1-6HA-GFP-URA3-YWP1*; wall-anchored Ywp1; **secretes Ywp1(aa1-520)-6HA-Gfp**	ETY3	3G9
	*YWP1-6HA-YWP1 / YWP1-6HA-GFP-URA3-YWP1*; wall-anchored Ywp1-6HA-Ywp1; **secretes Ywp1-6HA-Gfp**; increased exposure of epitopes	ETY3e13	4F1
	*YWP1-6HA-YWP1 / YWP1-3HA-GFP-URA3-YWP1*; wall-anchored Ywp1-6HA-Ywp1; **secretes Ywp1-3HA-Gfp**; increased exposure of epitopes	ETY3e13	4F6a
**YHGY**	*YWP1 / YWP1-3HA-GFP-YWP1*; **wall-anchored** Ywp1 and **Ywp1-3HA-Gfp-Ywp1**	M1796 (*Δsap9*,*10*)	β3S1i’ΔU1a
	*YWP1-GFP-YWP1 / YWP1-6HA-YWP1*; **wall anchored Ywp1-Gfp-Ywp1 and Ywp1-6HA-Ywp1**	ETY3	βH3b1ΔU1c
	*YWP1-6HA-YWP1 / YWP1-6HA-GFP-YWP1*; **wall-anchored** Ywp1-6HA-Ywp1 and **Ywp1-6HA-Gfp-Ywp1**; increased exposure of epitopes	ETY3e13	βH4a1aΔU1

Y Ywp1; G Gfp (inserted on C-terminal side of aa 165 of Ywp1, except as indicated for insertion on C-terminal side of aa 520); H HA epitope assembly (3HA or 6HA); U *URA3* inserted to disrupt transcription of upstream *YWP1* or *GFP*, and to confer uracil prototrophy; ETY Epitope Tagged Ywp1; ETY1 and ETY3 are subclones of strain M1793; c control strain (contemporary subclone); x strain with increased epitope exposure; β strains that were transformed with the bifunctional *YWP1-GFP-URA3-GFP-YWP1* cassette; Δ deleted or disrupted; ΔU *URA3* lost from β transformants, allowing flanking GFP segments to join; ΔY *YWP1* disrupted *upstream* from HA and Gfp insertion sites; mix Strain ETY3eS is a mixture of YHY and YHYx

### Culture media

Cells were grown in the following media: Rich medium (YPD: 1% yeast extract, 2% peptone, 2% dextrose); minimal medium 13 (MM13: 100 mM glucose, 80 mM NH_4_Cl, 5 mM NaCl, 5 mM KH_2_PO_4_, 1 mM MgSO_4_, 10 μM biotin, 0.5 mM succinic acid, 0.2 mM CaCl_2_, 2 μM FeCl_3_, 1 μM ZnCl_2_, 0.2 μM MnCl_2_, 0.2 μM CuCl_2_ [22]); buffered MM13 (BMM13: MM13 containing 100 mM MES [morpholinoethanesulfonate] and 50–100 mM [routinely 80 mM] Bis-Tris [bis-hydroxyethylimino tris-hydroxymethyl methane], pH 5.9–6.4); filamentation medium (RPMI-1640 containing 11 mM dextrose but no bicarbonate or phenol red, supplemented with 0.3 mM uridine, 25 mM HEPES and 15 mM NaOH, pH 7.7 [21]). Supplemental 1 mM arginine, 1 mM histidine and 0.5 mM uridine were included as necessary for auxotrophies. The phosphate concentration of BMM13 was sometimes reduced to less than 2 mM, the amount that is normally assimilated by batch cultures of MM13 prior to growth cessation [22]. For some experiments, BMM13 was supplemented with Tween-80 (0.01–0.02%) as a presumptive carrier to inhibit adsorptive loss of secreted Ywp1 [21], or 0.01–0.02% Pluronic® F-127 (Poloxamer 407, a 10–15 kDa triblock copolymer of polyoxyethylene and polyoxypropylene) to inhibit adsorption of the cells to plastic surfaces. Carbon source substitution in BMM13 involved replacement of the glucose with 100 mM lactic acid, elimination of the MES, and inclusion of 50 mM Bis-Tris. Media were sterilized by 0.2 μm filtration (all) or autoclaving (YPD only). For surface growth, media were hardened in Petri plates with 1.5% agar or 2.0% agarose.

### Antibodies

**Primary antibodies:** Mouse monoclonal: Anti-HA (BAbCO/Covance Research Products MMS-101R; HA.11 clone 16B12; IgG_1_); anti-β-1,3-glucan (Biosupplies Australia #400–2; IgG_1_); anti-Gfp (Developmental Studies Hybridoma Bank DSHB-GFP-4C9, -8H11 and -12A6); anti-β-1,2-mannotriose (B6.1 IgM, C3.1 IgG_3_ and G11.1 IgG_1_ [34–36]). Mouse antisera were all elicited by DNA vaccination with plasmids encoding *Candida albicans* polypeptides: Ywp1 aa 1–533, aa 21–116, aa 51–197 and aa 105–161; MP65 aa 17–378; Pho100 aa 18–323; and orf19.3621 aa 1–83 [22]. No monoclonal antibodies specific for Ywp1 are known to exist. **Secondary antibodies:** Goat anti-mouse IgG (H+L) conjugated to DTAF (dichlorotriazinyl aminofluorescein; Jackson ImmunoResearch), Alexa Fluor® 488 or Alexa Fluor® 568 (Molecular Probes); F(ab’)_2_ fraction of goat anti-mouse IgG (H+L) conjugated to Alexa Fluor® 594 (InVitrogen); monoclonal rat anti-mouse IgG_1_ conjugated to eFluor660 (eBioScience clone M1-14D12). Absorption/emission maxima (nm) of these fluorochromes: DTAF (493/519), Alexa Fluor® 488 (496/519), Alexa Fluor® 568 (578/603), Alexa Fluor® 594 (590/617), eFluor660 (633/668). The Alexa Fluor® 568 and 594 conjugates were not appropriate for the available flow cytometer lasers and filters, but otherwise usage was based largely on availability of the conjugates at the time of each experiment.

### Cell fixation and immunolabeling

Cell fixation procedures were designed to minimize the loss of intrinsic Gfp fluorescence, and began with an adjustment of the extracellular pH to 8, either by adding buffer directly to culture aliquots or by suspension of centrifugally pelleted cells. For formaldehyde fixation, pelleted cells were suspended in 200 mM HEPES (hydroxyethylpiperazine ethanesulfonate), 160 mM NaOH, 5 mM KCl, 0.01% Pluronic® F-127, pH 8.0, then mixed with 0.016 volumes of formalin (giving a final concentration of 200 mM formaldehyde), and incubated 1–2 hr at 20–25°C with rotation to keep the cells in suspension. The cells were then pelleted and suspended in 50 mM Tris (tris-hydroxymethyl aminomethane), 10 mM EDTA, 10 mM NaCl, 10 mM KCl, pH 8.0 (TESP) to remove and neutralize the remaining aldehyde. Alternatively, cells suspended in the pH 8 TESP or HEPES buffer were fixed by heating (60°C for 10–15 min) or by rapid mixing with an equal volume of the same buffer containing 80% ethanol (giving a final ethanol concentration of 40%) or by direct suspension in TE containing 40% ethanol. All three fixation procedures killed all of the cells. Subsequent washes and incubations with antibodies (typically 1–2 hr at 20–25°C for both primary and secondary antibodies) were in TESP containing 0.1% Triton X-100 or Nonidet P-40 (nonionic surfactants that reduced cell losses but otherwise had no discernable effects on antigen accessibility or other properties).

### Microscopy and flow cytometry

Differential interference contrast (DIC) and epifluorescence microscopy were performed with a Nikon Eclipse 80i microscope equipped with oil immersion apochromatic 40× and 60× objective lenses (numerical apertures 1.0 and 1.4), three filter cubes (FITC Ex465-495 DM505 BA515-555; TRITC Ex540/25 DM565 BA605/55; and Cy5 Ex620/60 DM660 BA700/75) and a multiband filter cube (FITC/TRITC). Micrographs were captured with a DS-Ri1 camera and NIS-Elements BR Imaging Software (3.10 SP3 Hotfix2). Images were cropped, resized and sometimes overlayed in Adobe Photoshop, but were not otherwise modified.

Flow cytometry was performed with a Becton-Dickinson BD Accuri™C6 flow cytometer with BD Accuri™C6 software (version 1.0.264.21) using slow sample uptake (14μl/min; 10μm core) and a FSC-H threshold of 200,000. The cytometer was equipped with 488 nm and 640 nm excitation lasers and the following emission filters: FL1 533/30, FL2 585/40, FL3 >670, and FL4 675/25.

Cell fixation and immunolabeling are described above; unfixed cells were adjusted to pH 8 immediately before analysis.

### Protein analysis

Production and analysis of Ywp1 and its derivatives were performed as described previously [21, 22]. The single N-glycan of Ywp1 (linked to N115 of the Ywp1 propeptide) was enzymatically removed with peptide-N-glycanase F (PNGase F; New England Biolabs). Sodium dodecyl sulfate polyacrylamide gel electrophoresis (SDS-PAGE) utilized modifications [21] that preserved Gfp fluorescence and allowed resolution of the deglycosylated Ywp1 propeptide at moderate acrylamide concentrations; briefly, its components were: Cathode buffer (100 mM Tris, 100 mM glycine, 0.1% SDS, pH 9.1); anode buffer (200 mM Tris, 50 mM HCl, pH 8.8); stacking gel (5% acrylamide, 0.13% bis-acrylamide, 200 mM Tris, 100 mM HCl, 0.1% SDS, pH8.3); and resolving gel (15% acrylamide, 0.15% bis-acrylamide, 400 mM Tris, 100 mM HCl, pH8.8). Polymerization of degassed acrylamide solutions was initiated with ammonium persulfate and TEMED. Samples were loaded with final concentrations of 200 mM Tris, 100 mM HCl, 1% SDS, 20 mM DTT (except as indicated), 2% Ficoll 400, and 40 μM Phenol Red. Visualization of in-gel Gfp fluorescence by 488 nm laser scanning was performed as described previously [21] using a GE Typhoon Trio with its PMT set at 350 and emission detection at 510 nm. Protein was visualized after gel fixation with 40% ethanol, 10% acetic acid, staining in the same mixture containing 0.1% Coomassie Blue R-250, and destaining with 15% ethanol, 5% acetic acid.

### Gene engineering

*Candida albicans* strains secreting Gfp-tagged versions of Ywp1 (Ywp165-Gfp and Ywp520-Gfp) were created as described previously [21], except the transforming DNA contained *GFP-URA3* rather than *GFP-HIS1*. The *GFP* that was used encoded yeast enhanced Gfp with codon optimization for *C*. *albicans* and the S65G and S72A mutations [37]. A gel-purified 2.16 kbp ApaI-HindIII fragment of pGT-GFP-URA3-14 (GenBank accession AY656808 [22]) served as the template for PCR amplifications of transforming cassettes using forward primer T1G1-5DR’ or TEP1-5GFP2 [21] coupled with a reverse primer (YWP1-3GU1: *aagactcttcaacttctgttcatgatagttggtataatgattgtaaggacagaatt****gaag*gaccacctttgattg**) that matched the 3’ untranslated regions of *YWP1* (*italicized*) and *URA3* (**bold**); amplicons were transfected into *ura3*-negative YHY strains, and recombinants were selected for growth in the absence of exogenous uracil or uridine.

A bifunctional template plasmid was created to allow both C-terminal and internal insertions of Gfp into any protein following a single transfection, and thus, in the present study, to generate both secreted and wall-anchored versions of Gfp-tagged Ywp1. A 199 bp segment of *GFP* (encoding amino acids 168–233) was amplified from an RsaI digest of pGT-GFP-URA3-14 using primers GFPC-F1 (gctctagacacaacattgaagatggttctg) and GY-R1 (cagcatgctgggcccagaacccgatggggaagcagaactcataccatgggtaataccagc); this amplicon was ligated into pGT-GFP-URA3-14 (after cutting both with ApaI and XbaI) to form pGEM-GFP-URA3-GFP (S1 File), a plasmid that can now be obtained from Addgene (www.addgene.org/72606/). A gel-purified 2.33 kbp HindIII-ApaI fragment of pGEM-GFP-URA3-GFP then served as the template for PCR amplification of *GFP-URA3-GFP* using primers T1G1-5DR’ [21] and YWP166-R (agatctgatagtagcagaatcagaaccagaaccggattcggaaccagatggagaagcagaacccgatggggaagcagaac); amplicons were transfected into *ura3*-negative strains, and recombinants were selected for growth in the absence of exogenous uracil or uridine. Transformants were monitored for secretion of Ywp1-Gfp using a Photon Technology International fluorometer; supernatants of phosphate-limited stationary phase cultures were adjusted to pH 8 with 0.087 volumes of 2.5 mM Tris, 25 mM EDTA and scanned for fluorescence emission between 504 and 522 nm upon excitation by 468 nm light, with a peak emission at 510–511 nm indicating the presence of Gfp. The anticipated genetic modifications were confirmed by PCR analyses of purified genomic DNA. Transformants were then plated on nutrient agar containing 5 mM 5-fluoroorotic acid (5-FOA) and 0.2 mM uridine to select for loss of the inserted *URA3*. Survivors were analyzed by fluorescence microscopy for Gfp fluorescence in their cell wall. PCR analyses of their genomic DNA confirmed that the *URA3* had been lost through recombination of the identical flanking *GFP* segments, which resulted in *GFP* bridging the upstream and downstream *YWP1* segments to form a single open reading frame encompassing all 534 codons of *YWP1* and codons 1–233 of *GFP*. Some of these heterozygotes (*YWP1 / YWP1-GFP-YWP1*) were then converted to *YWP1* hemizygotes by disrupting one of the two *YWP1* alleles, thus creating comparator strains that made only wild type Ywp1 or only Ywp1-Gfp-Ywp1. PCR amplicons made using primers YWP-5U and YWP-3U [21] were used to insert *URA3* upstream from the *GFP* insertion point and thus disrupt expression of that allele. Transformants were verified as above by analyzing their Gfp fluorescence and genomic DNA.

### Miscellany

Yeast biofilm spot adhesion assays were performed as described previously [21, 22]. DNA was commercially sequenced by GenScript or Eurofins. Oligodeoxynucleotides were synthesized by Integrated DNA Technologies. Caspofungin acetate was from ApexBio Technology. Calcofluor White (Fluorescent Brightener 28, F-6259) was from Sigma Chemical Co. Recombinant, protease-free β-1,3-glucanase (Quantazyme™ *ylg*) was from Quantum Biotechnologies.

## Results

### Accessibility of Ywp1 to antibodies

Mouse antisera specific for the Ywp1 polypeptide of *Candida albicans* previously showed little if any binding to intact yeast cells that had abundant Ywp1 in their cell walls, presumably because of epitope-masking by wall polysaccharides [22]. Recent reassessment, however, revealed that for several of the same strains of *C*. *albicans*, a small proportion of yeast cells (estimated to be 10^−3^ to 10^−6^) strongly bound some of these antisera. Such cells were observed to be especially abundant in a current laboratory culture of strain BWP17. When 120 subclones of BWP17 were isolated and screened by immunofluorescence microscopy, four were found to have a stable phenotype of strong binding to anti-Ywp1. These four subclones were designated BWP17x; two of the non-binding subclones were saved as controls and designated BWP17c, representing parental strain BWP17. Within each of these two groups, no difference was found in any subsequent assay, so each group’s members were regarded as representative of that strain.

**Fig 1:**
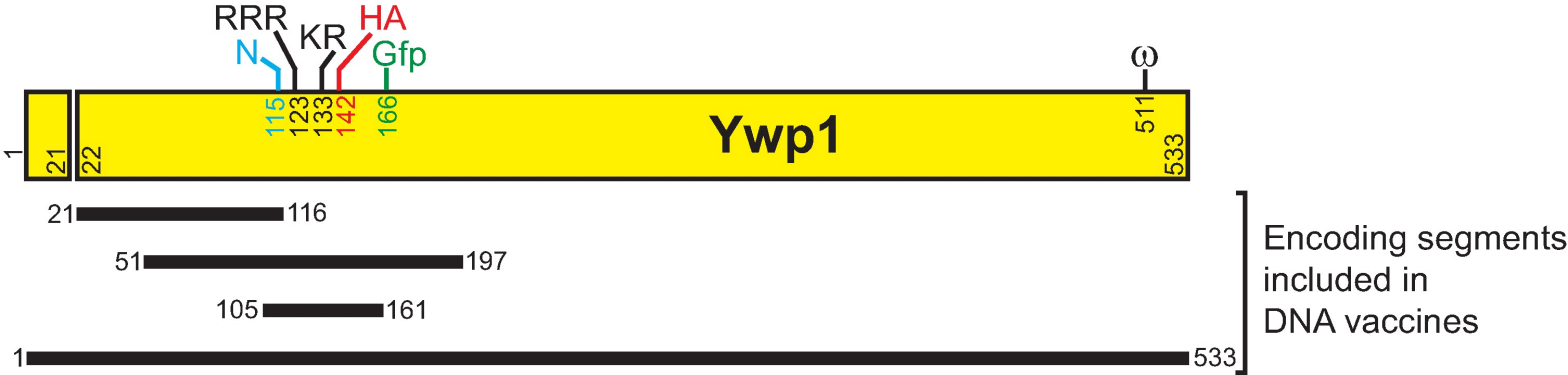
Schematic diagram of Ywp1 and its encoding segments that were included in DNA vaccines. Four segments of *YWP1*, encoding all 533 amino acids of Ywp1 or just amino acids 21–116, 51–197 or 105–161, were included in DNA vaccines for the elicitation of specific antibodies in mice [22]. Features of Ywp1 are noted here for their locations relative to these segments: The cleaved signal peptide (aa 1–21), the sole site for N-glycan addition (N115), the tribasic and dibasic propeptide cleavage sites (RRR and KR), the sites for insertion of a fluorescent tag (Gfp) and an epitope tag (HA) (described in subsequent sections), and the site of GPI anchorage (ω511), a remnant of which mediates covalent linkage of Ywp1 to the cell wall matrix.

The tested anti-Ywp1 antisera were derived from mice that had been DNA-vaccinated with plasmids encoding the full-length Ywp1 polypeptide (aa 1–533) or shorter segments of Ywp1 (amino acids 21–116, 51–197 or 105–161) [22] (Fig 1). Only antibodies against the full-length Ywp1 (from each of three different mice) were found to bind strongly to BWP17x yeast cells; antibodies against the shorter segments of Ywp1 bound poorly if at all, as did DNA vaccine-elicited antibodies specific for three other *C*. *albicans* antigens (Pho100 aa 18–323, MP65 aa 17–378, and orf19.3621 aa 1–83, as detailed below).

**Fig 2:**
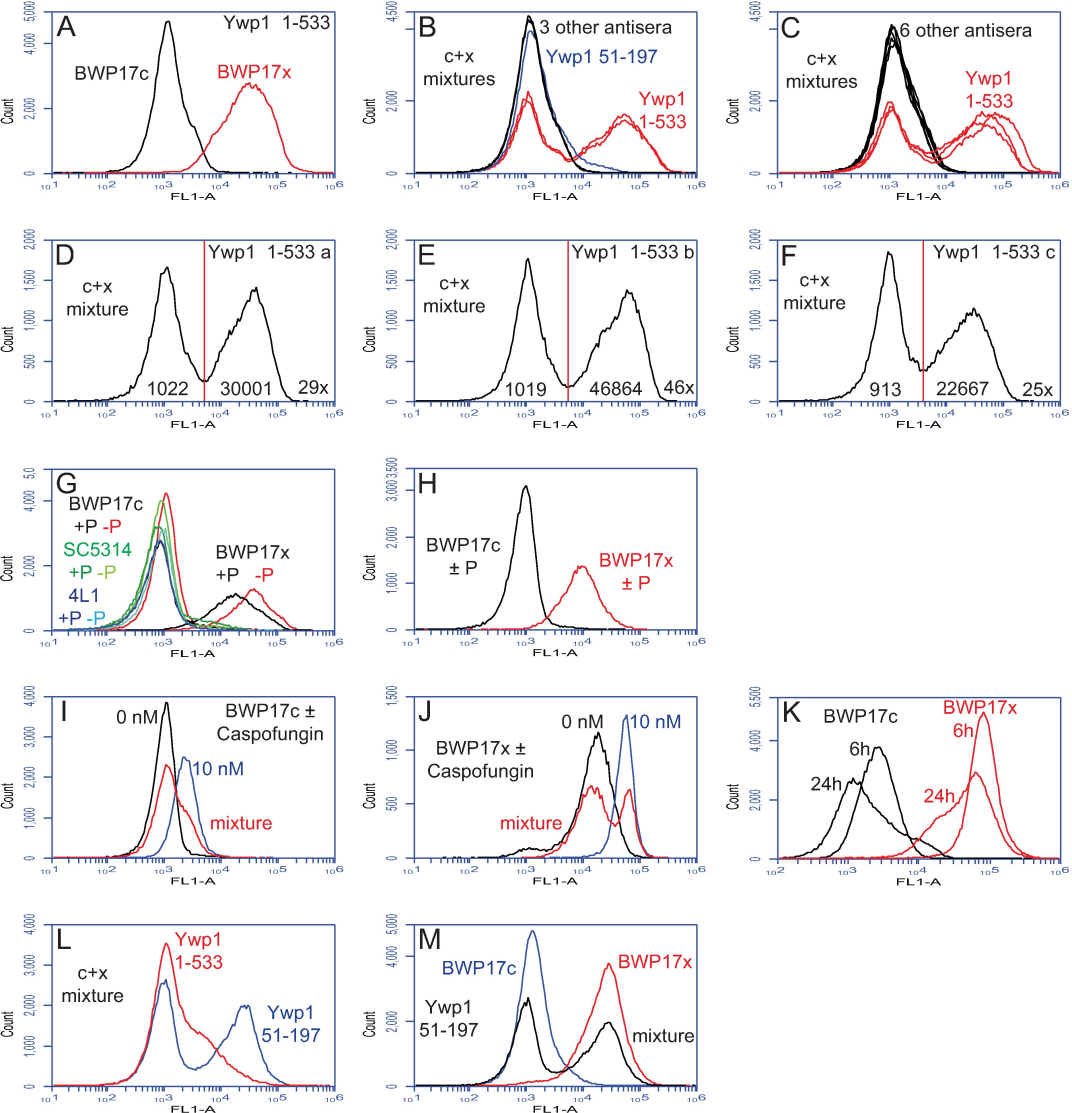
Enhanced binding of anti-Ywp1 to a strain variant. Parental (BWP17c) and variant (BWP17x) strains were compared by flow cytometry. Yeast cells were grown at 30°C in rich medium (YPD) or buffered minimal medium 13 (BMM13) [22] under a variety of conditions, as indicated. BMM13 had an initial phosphate concentration that was either limiting (0.0–0.3 mM; “-P”; all panels, except as indicated) or in surplus (2.5–5.0 mM; “+P”). Except for panels L and M, cells were fixed with formaldehyde prior to labeling with sera from DNA vaccinated mice and fluorescein-conjugated goat anti-mouse IgG. In some cases, for more accurate quantitation, the two strains (“c + x”) were mixed together prior to antibody application. (A-F) Compared to strain BWP17c, BWP17x bound far more anti-Ywp1 (aa 1–533; from each of three different mice), but only slightly more anti-Ywp1 (aa 51–197), and no more anti-MP65 (2 mice), anti-Pho100, anti-orf19.3621 (from a Ywp1 pseudogene), anti-Ywp1 (aa 21–116; 3 mice), or anti-Ywp1 (aa 105–161). (G,H) Phosphate limitation (which increases *YWP1* expression) had a comparatively modest effect on the amount of anti-Ywp1 (aa 1–533) bound; included in this experiment for comparison were wild type strain SC5314 and Ywp1-negative strain 4L1. In (H), fixed +P and–P samples were mixed together prior to antibody labeling. (I,J) Growth in the presence of Caspofungin resulted in more anti-Ywp1 (aa 1–533) binding. (K) Binding of this antibody declined overall as the cultures progressed from exponential growth to stationary phase. (L,M) Extraction of cells with SDS at 70°C (without prior or subsequent fixation) resulted in diminished binding of anti-Ywp1 (aa 1–533) to strain BWP17x, but greatly enhanced binding of anti-Ywp1 (aa 51–197); peak identities were confirmed by analyzing strains separately as well as in mixtures. The populations shown in these panels include all events (A,B,C,K,L,M; 100,000 per sample) or have been gated to exclude aggregates or multiplets (D,E,F,G,H,I,J).

Flow cytometry was used to quantify the difference in anti-Ywp1 binding between BWP17c and BWP17x. When the two strains were assayed separately, the difference was striking (Fig 2A). This difference was semi-quantitative because relevant molecular parameters (the concentration of anti-Ywp1 in the serum, concentration of accessible Ywp1 epitopes in the cell sample, kinetics of binding, and degree of saturation of binding sites) were unknown, and not feasibly discerned due to the limited amount of each antiserum available. Therefore, the two BWP17 strains were mixed together after fixation but before antibody application so that all cells would experience exactly the same concentration of unbound antibody for the same period of time, and each cell’s signal would be proportional to its number of available epitopes. Several antisera were analyzed in parallel each time, as indicated (Fig 2B and 2C). The wide separation of the peaks allowed an estimation of 48 and 45 times more binding of anti-Ywp1 (aa 1–533) to BWP17x than to BWP17c (mean and median of 8 experiments with the three antisera on 5 different days; range 33–65). Ignoring events that appeared to be aggregates of cells (i.e., including only singlet cells and unseparated mother/daughter doublets by appropriate gating on plots of FSC-A *vs* SSC-A or FSC-A *vs* FSC-H) resulted in means and medians of 29 and 26 times more binding of anti-Ywp1 to BWP17x (range 16–46; Fig 2D–2F).

The above results describe cells that were grown as yeast forms in 30°C buffered (pH~6) synthetic minimal medium and fixed with formaldehyde prior to antibody labeling. Similar results were obtained after fixation with just heat (60°C) or ethanol (40% v/v), except only about half as much antibody bound to BWP17x. Similar results were also obtained after growth of the cells in a range of media, including unbuffered (pH~3) minimal medium, rich medium (YPD), and medium in which lactate replaced glucose as the sole carbon source, with 11–23 times more anti-Ywp1 (aa 1–533) binding to BWP17x than to BWP17c (range of means for singlets). In some experiments, there were subtle differences in cell size and granularity between BWP17c and BWP17x (as measured by light scattering), but these differences were quantitatively less than a multiple of 2, and had little effect on the calculated fluorescence ratios (see S1 Fig). Thus, the observed differences in antibody binding appear to be robust over a wide range of experimental conditions.

Phosphate starvation has been shown to increase *YWP1* expression by a factor of 2–3 [22, 38]; it also doubled the binding of anti-Ywp1 binding to BWP17x, with a more modest effect on BWP17c (Fig 2G). More accurate quantitation using mixtures of cells was not possible because phosphate-limited and phosphate-replete cultures did not generate distinguishable peaks for either subclone (Fig 2H). When grown in the presence of a subinhibitory dose of Caspofungin (10 nM), which subtly modifies the cell wall [14], both BWP17c and BWP17x bound more anti-Ywp1 (2–4 times more in stationary phase) (Fig 2I and 2J). In shaking liquid cultures, the growth phase was found to influence anti-Ywp1 binding to both BWP17c and BWP17x; nearly twice as much anti-Ywp1 bound to cells from young (exponentially growing) cultures than to cells from older (at or near stationary phase) cultures (Fig 2K), thus revealing a significant effect of cell age on Ywp1 accessibility.

Interestingly, one treatment was found that altered the binding of two of the anti-Ywp1 sera. Heating of unfixed cells to 70°C in the presence of SDS has previously been shown to dissociate and liberate the 99–101 aa propeptide of Ywp1, which is noncovalently associated with the wall-anchored core of Ywp1 [21]; this treatment was also found to diminish binding of anti-Ywp1 (aa 1–533), but enhance binding of anti-Ywp1 (aa 51–197) (Fig 2L and 2M). Since the Ywp1 core that remains anchored in the cell wall after this treatment consists of aa 134–511, this treatment evidently exposed epitopes (aa 135–197) recognized by one antiserum while eliminating most of the epitopes or epitope conformations recognized by the other antiserum.

**Fig 3:**
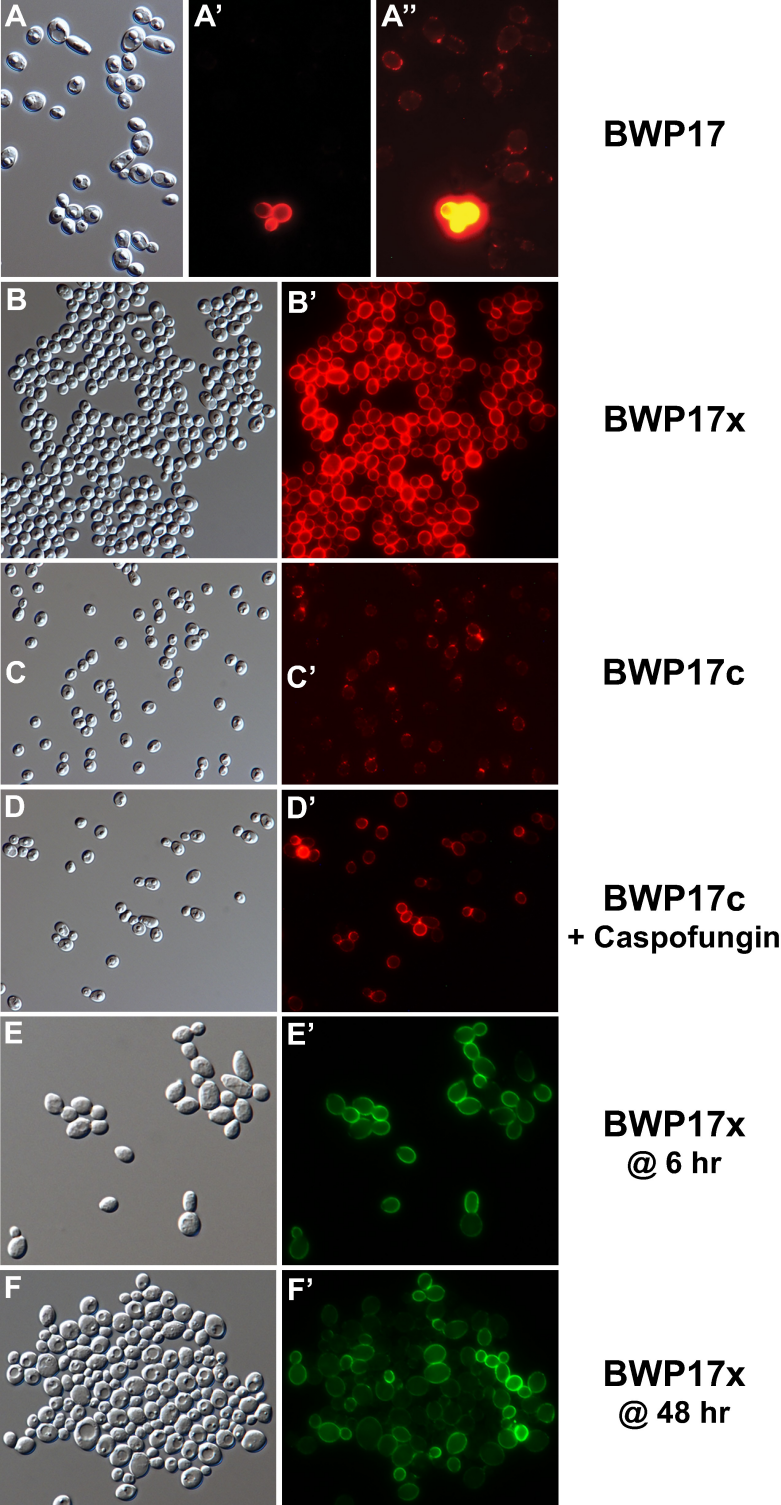
Micrographs of BWP17c and BWP17x labeled with anti-Ywp1. Differential interference contrast (DIC) light micrographs (A-F) and corresponding epifluorescence micrographs (A’-F’) of yeast forms grown to stationary phase in BMM13 (A-D, A’-D’), or in YPD for 6 hr (E,E’) or 48 hr (F,F’). Cells were fixed with formaldehyde and labeled with mouse anti-Ywp1 (aa 1–533) serum followed by goat anti-mouse IgG conjugated to AlexaFluor 568 (A’,A”), AlexaFluor 594 (B’,C’,D’), or fluorescein (E’,F’). (A-A”) BWP17 field showing rare cells intensely labeled with anti-Ywp1; A” was sampled 20× longer than A’ to show faint signals from the majority cells. Subclones BWP17x (B,B’) and BWP17c (C,C’) show contrasting differences in amount of anti-Ywp1 binding; C’ was sampled 30× longer than B’. A parallel culture of BWP17c (C,C’) was given 10 nM Caspofungin for its final 24 hr of growth (D,D’), which resulted in greater anti-Ywp1 binding. D’ was sampled for 1/5 the time of C’. BWP17x cells grown 6 hr (E,E’) or 48 hr (F,F’) show labeling in accordance with culture age. Actual width of each image (μm): A: 55; B: 104; C: 127; D: 127; E: 58; F: 73.

Epifluorescence microscopy of individual cells detailed spatial aspects of the binding of anti-Ywp1 to yeast cells (Fig 3). Fig 3A–3A” show the rare cells of strain BWP17 that bind larger quantities of anti-Ywp1; only upon extreme photographic overexposure of these cells do the surrounding majority cells show fluorescence, but at a level that is difficult to distinguish from nonspecific antibody binding. The BWP17x subclone (Fig 3B and 3B’) shows strong labeling of the cell wall with anti-Ywp1, while a 30× longer exposure shows patchy labeling of subclone BWP17c (Fig 3C and 3C’). Much of that low signal tended to be concentrated at bud necks and presumptive bud or birth scars. As demonstrated above by flow cytometry, growth in the presence of Caspofungin resulted in more anti-Ywp1 binding to BWP17c (Fig 3D and 3D’), but still at a level below that of BWP17x (6× longer exposure for Fig 3D’ than Fig 3B’). The increased anti-Ywp1 binding to young cells relative to older cells (flow cytometry Fig 2K) appears to be due in part to greater epitope presence or accessibility on nascent daughter cells (Fig 3E and 3E’), followed by an overall loss of signal as the cultures transition into stationary phase (Fig 3F and 3F’).

BWP17c and BWP17x were further examined to determine if some change in Ywp1 itself were responsible for the difference in anti-Ywp1 binding. As a proxy for the full Ywp1, the cleaved propeptide of Ywp1 showed no significant difference between the two strains: As observed previously [21, 22], there was little shed into buffered cultures, and SDS dissociated most of the propeptide from intact cells at 70°C but not at 50°C (S2 Fig). There was little difference in the quantity of extracted Ywp1 propeptide between the two strains, but for BWP17x there was an increase in the amount of total protein extracted by the SDS treatments. Thus, the Ywp1 epitope accessibility difference seems likely to be a result of a structural difference in the cell wall that is independent of Ywp1, and not because BWP17x has more Ywp1 in its wall than does BWP17c. For additional confirmation of this, the *YWP1* alleles were amplified from purified genomic DNA by PCR and sequenced for both BWP17c subclones and two of the four BWP17x subclones. Both *YWP1* alleles in all four of these subclones exactly matched the sequence of *YWP1* on chromosome 2A of strain SC5314 in Assembly 22 of the Candida Genome Database [39]. (*YWP1* on SC5314 chromosome 2B has three silent, single-nucleotide differences from *YWP1* on chromosome 2A; a loss-of-heterozygosity event [40] is known to have left strain BWP17 with just one version, the 2A version.) This, along with its Arg/His/Ura triple auxotrophy, lends further support to the identification of BWP17x as a spontaneous variant of BWP17 rather than simply a contaminant.

### Other correlations of Ywp1 accessibility

**Fig 4:**
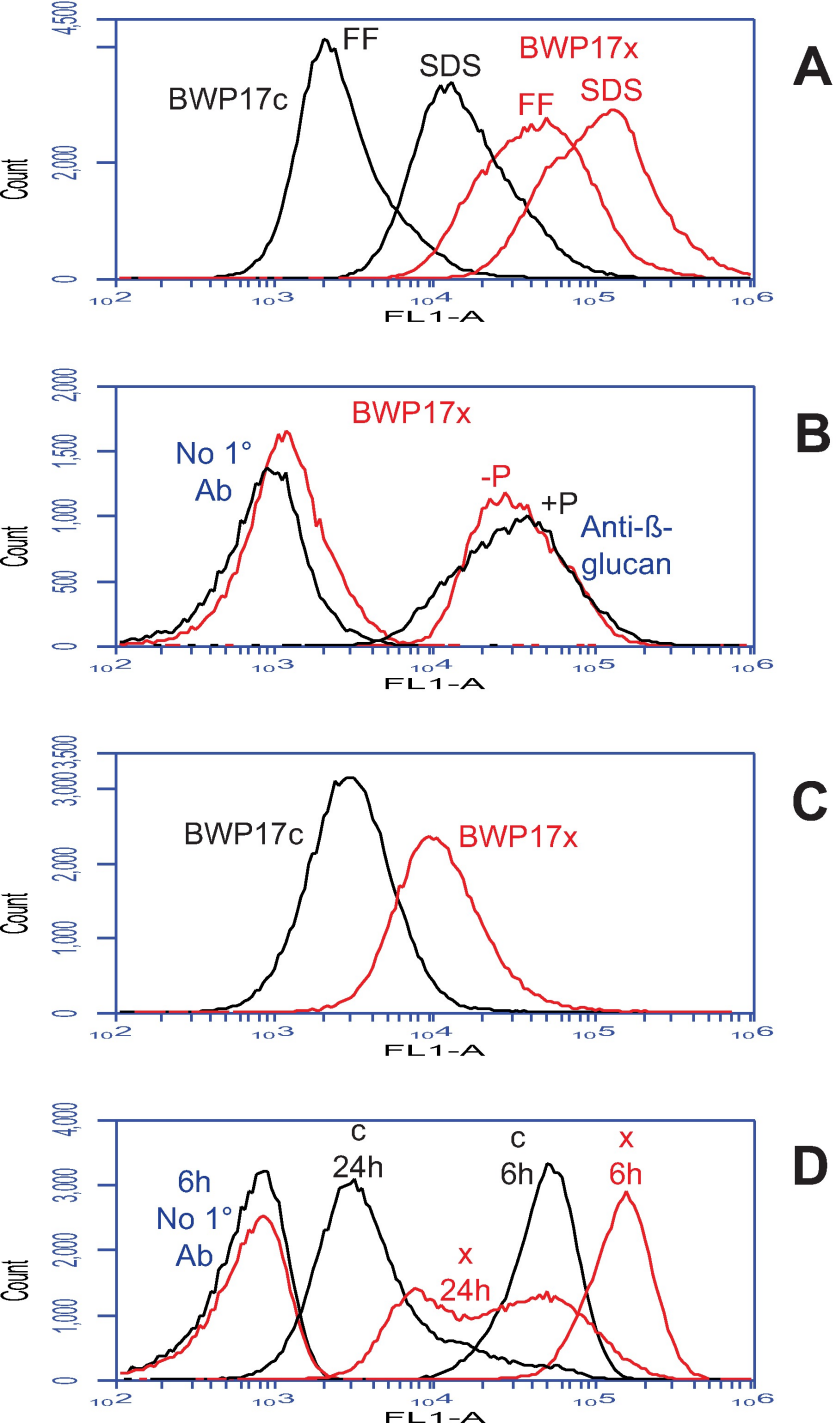
Strain BWP17x also binds more anti-β-1,3-glucan. Flow cytometric analysis of parental (BWP17c) and mutant (BWP17x) yeast forms after labeling with a monoclonal mouse anti-β-1,3-glucan IgG followed by fluorescein-conjugated goat anti-mouse IgG. Cells were cultured as in Fig 2. (A) Relative to formaldehyde fixation (FF), extraction with SDS at 70°C enhanced anti-β-1,3-glucan binding to both BWP17c and BWP17x, but BWP17x showed far more binding than BWP17c after both treatments. (B) Phosphate limitation had little effect on anti-β-1,3-glucan binding; also shown in this panel are controls that omitted the primary antibody (1° Ab) but included the secondary (fluorescein-conjugated) antibody. (C) Replacement of glucose with lactate as the sole carbon source in the growth medium diminished anti-β-1,3-glucan binding to BWP17x, but to a level that was still almost 4× that of BWP17c. (D) Cells in early phase cultures (6 hr in YPD) showed more binding of anti-β-1,3-glucan than cells at later phases (shown for 24 hr); the later phases showed much more variability, however, based on the width of the profiles. Populations in panels B, C and D were gated as in Fig 2 to exclude aggregates and multiplets.

A stable difference in the cell wall that differentially exposes Ywp1 epitopes may have other measurable manifestations, and this was found to be the case for β-glucan exposure, environmental resistance, and cell adhesion. The molecular basis for these differences is not yet known, but the phenomena are documented here as a foundation for further exploration of the roles of β-glucan and Ywp1 in the structure of the cell wall. A monoclonal antibody specific for β-1,3-glucan [41] was found to have much greater binding to BWP17x than to BWP17c; this was observed for stationary phase yeast cells after formaldehyde fixation as well as after extraction of unfixed cells with SDS at 70°C (Fig 4A). In this semi-quantitative experiment where cell populations were assayed individually rather than as mixtures, the 70°C SDS treatment resulted in 5–6× more anti-β-glucan binding to BWP17c, and 2–3× more binding to BWP17x (relative to formaldehyde fixation), but after each treatment there was still much more binding to BWP17x than to BWP17c (13× for formaldehyde fixation and 5× for SDS treatment). Phosphate limitation was found to have little effect on anti-β-glucan binding (Fig 4B). When glucose was replaced with lactate as the sole source of carbon for growth, affecting cell wall architecture [42], a factor of 4 difference between BWP17c and BWP17x persisted (Fig 4C). Even more so than for anti-Ywp1 binding, anti-β-glucan binding was influenced by the growth phase of the cells in liquid culture, with more anti-β-glucan binding to early phase cells than to late phase cells (Fig 4D; these are the same fixed cell samples as in Fig 2K).

When grown on solid agar containing rich (YPD) medium, BWP17x was observed to be much more sensitive to Calcofluour White, slightly more sensitive to Congo Red and Caspofungin, and slightly less sensitive to SDS than was BWP17c (S3A Fig), again indicating differences in cell wall structure. A yeast biofilm spot adhesion assay has been used previously to demonstrate enhanced adhesion of strains that lack Ywp1 [21, 22]; the same assay revealed that strain BWP17x also has enhanced adhesion, despite having normal amounts of wild type Ywp1 (S3B Fig).

**Fig 5:**
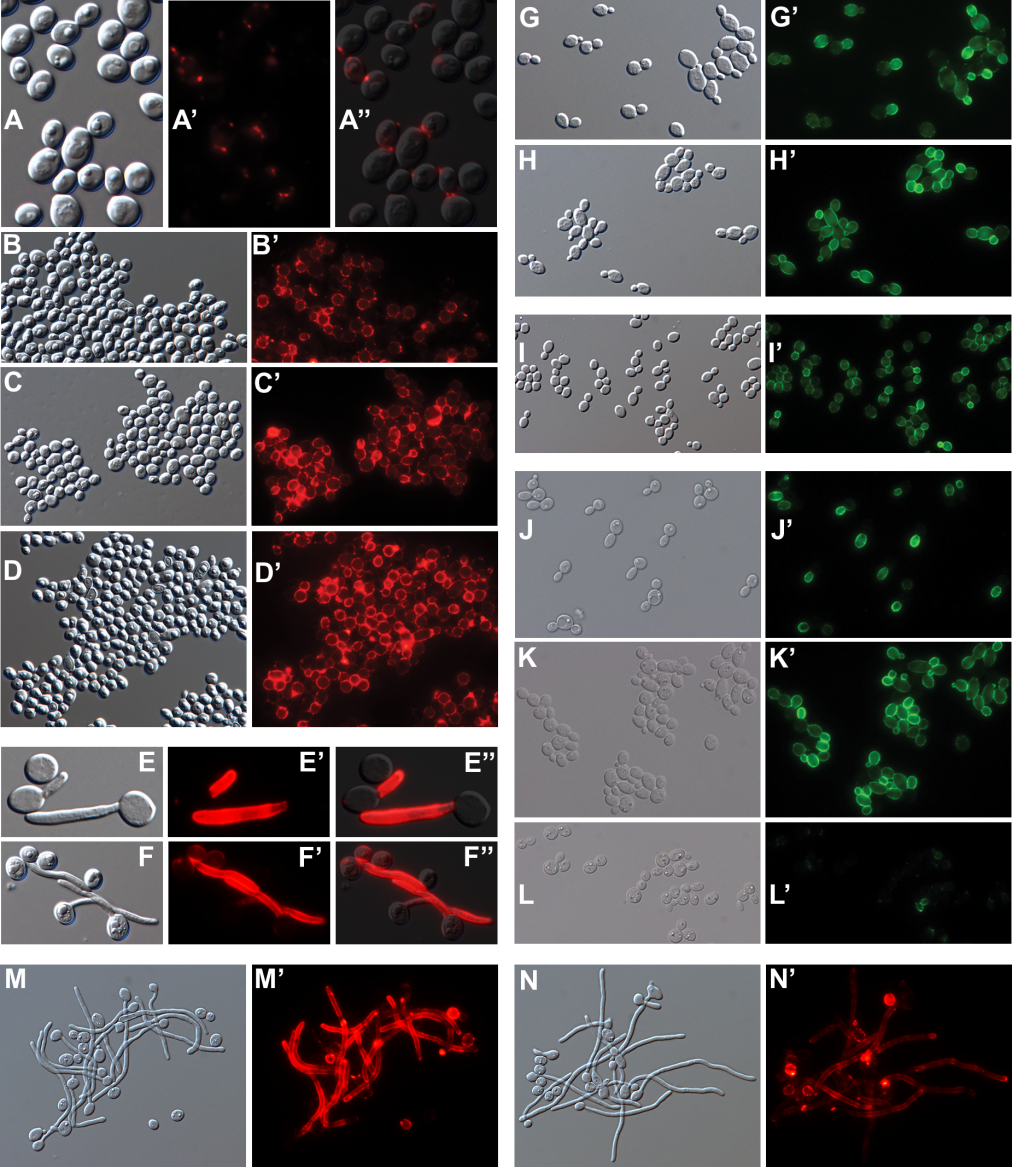
Micrographs of cells labeled with anti-β-1,3-glucan. DIC (A-N) and corresponding fluorescence (A’-N’) micrographs. The secondary antibody was goat anti-mouse IgG conjugated to either AlexaFluor 594 (A’-D’, M’, N’) or fluorescein (G’-L’). (A”, E”, F”) Composite overlay of DIC and fluorescence images. BWP17c (A,B) and BWP17x (C,D) grown as in Fig 3C and 3D without (A,C) or with (B,D) Caspofungin. BWP17c (E) and BWP17x (F) after 3 hr in 37°C RPMI filamentation medium. BWP17c (G) and BWP17x (H) after 6h in 30°C YPD medium. (I) BWP17x after extraction with SDS at 70°C. Ywp1-knockout strain 4L1 after growth in BMM13 for 4h (J), 8h (K) or 14h (L). Strains devoid of Ywp1 (M) or ectopically producing Ywp1 in germ tubes and hyphae (N) after 3.5 hr in 37°C RPMI filamentation medium. Actual width of each image (μm): A: 27; B: 93; C: 97; D: 101; E: 30; F: 43; G: 83; H: 94; I: 132; J: 107; K: 91; L: 118; M, N: 102.

By epifluorescence microscopy, binding of anti-β-glucan to BWP17c was barely detectable, with most of the signal localized to bud necks (Fig 5A–5A”). Growth in the presence of Caspofungin increased the antibody binding, but it remained punctate and patchy, rather than uniform (Fig 5B and 5B’). Comparatively, binding of anti-β-glucan to BWP17x was visibly greater (Fig 5C and 5C’); this signal was increased further by growth in the presence of Caspofungin (Fig 5D and 5D’), but the labeling remained somewhat patchy and nonuniform. Notably, the binding of anti-β-glucan to yeast forms was far less than to nascent germ tubes of both BWP17c and BWP17x (Figs 5E–5E” and 8F–8F”). Young yeast cultures of both BWP17c and BWP17x showed enhanced binding of anti-β-glucan to nascent daughter cells (Fig 5G and 5G’; 5H and 5H’). Extraction of BWP17x with SDS at 70°C resulted in more overall binding of anti-β-glucan (flow cytometry Fig 4A), but did not eliminate intercell or intracell nonuniformities (Fig 5I and 5I’). These micrographs are thus consistent with the quantitative flow cytometric data and additionally reveal the sites of β-glucan accessibility.

### Ywp1 contributes to glucan masking

**Fig 6:**
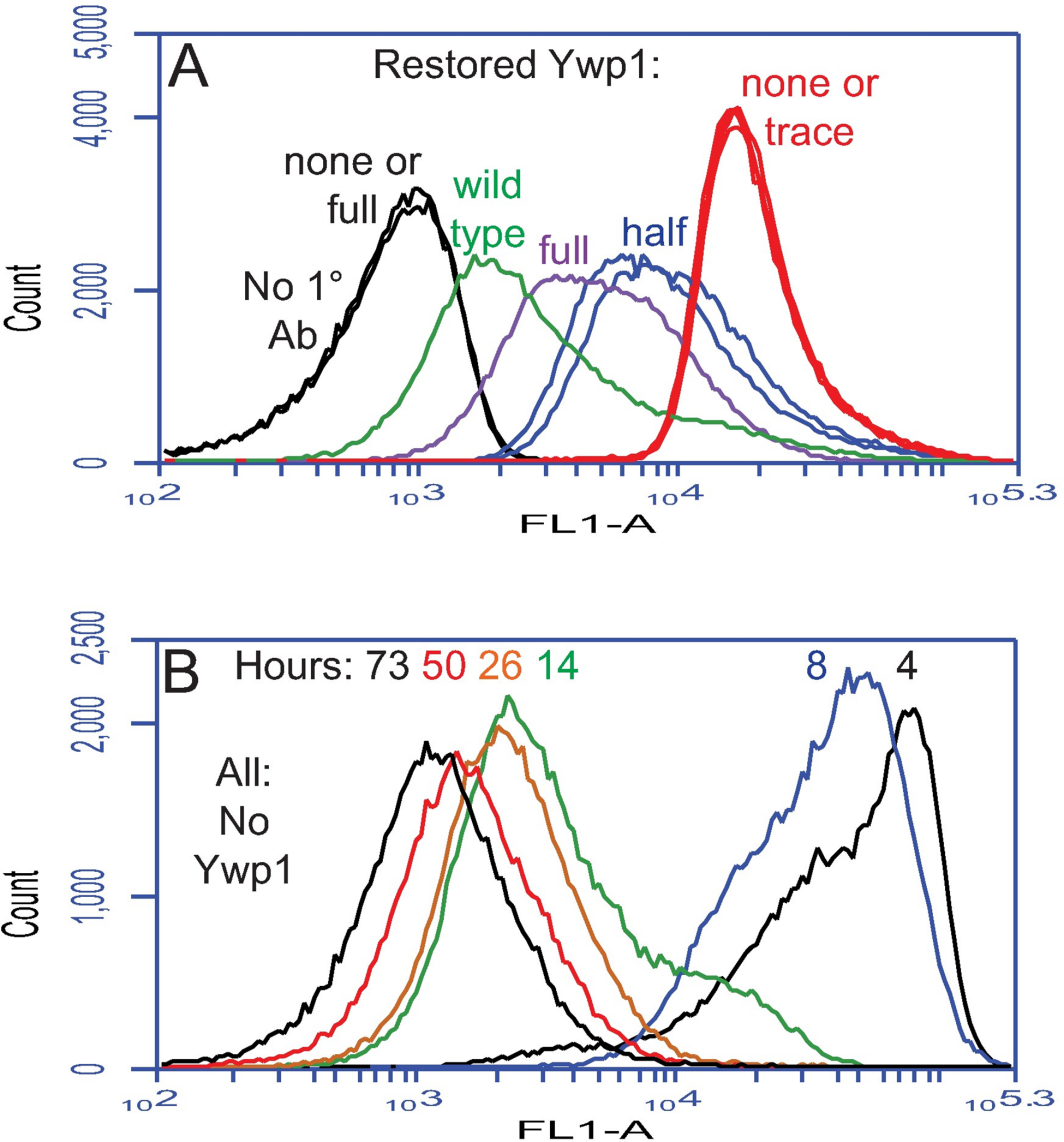
Loss of Ywp1 from the cell wall enhances binding of anti-β-1,3-glucan. (A) A set of Ywp1 knockout and restored strains [22] was grown for 18 hr in YPD, fixed with formaldehyde, and labeled with anti-β-1,3-glucan as in Fig 4. A composite of ten flow cytometric profiles is shown. The quantity of Ywp1 was zero in knockout strains 3L1 and 4L1; attempted restoration of *YWP1* generated a set of strains with no Ywp1 (16r2), a trace of Ywp1 (7r1), approximately half the normal amount (16s1 and 16s2), and close to the full amount (16r1), as indicated. Wild type strain DAY185 is strain BWP17 that was restored to prototrophy [33]. For further comparison, strains 16r1 and 16r2 were also labeled with just the secondary antibody to quantify background levels of fluorescence (black lines). (B) Ywp1 knockout strain 4L1 was grown at 30°C in liquid BMM13 culture; aliquots were taken at 4–73 hr after 1/20 dilution of a stationary phase culture into fresh BMM13; cells were fixed with formaldehyde and labeled with anti-β-1,3-glucan as above.

The masking of cell wall β-1,3-glucans is thought to arise from the overlying mannoproteins. Since Ywp1 is an abundant mannoprotein of the yeast cell wall, the binding of anti-β-glucan to strains with varying quantities of wall Ywp1 was assessed. Examination of a previously described set of Ywp1 knockout and restored strains [22] revealed that partial or complete loss of Ywp1 indeed allowed proportionally more binding of anti-β-glucan to stationary phase yeast (Fig 6A). Numerous additional strains and growth conditions confirmed this dependence, with 2–4× more anti-β-glucan binding in the absence of Ywp1; as expected, this required anchorage of Ywp1 to the cell wall, as anchor-negative mutants of Ywp1 did not contribute to this β-glucan masking. Growth phase also influenced anti-β-glucan binding to Ywp1-negative cells, with a large reduction in binding from early to late cultures (Fig 6B); much of that signal was found on nascent daughter cells in exponentially growing cultures (Fig 5J and 5J’; 5K and 5K’), but declined on most cells thereafter (Fig 5L and 5L’).

Ywp1 thus contributes to β-1,3-glucan masking in yeast cells. As shown in Fig 5E and 5F, germ tubes (which have little or no Ywp1) exhibit greater β-1,3-glucan exposure than the yeast forms. Strains previously engineered to ectopically produce Ywp1 in germ tubes and hyphae [21] were therefore examined for relative β-1,3-glucan exposure. These strains were devoid of yeast-expressed Ywp1, but had *YWP1* under the control of the hyphal wall protein 1 (*HWP1*) promoter, which is highly upregulated and highly expressed upon filamentation [24, 43, 44]. Fig 5M and 5N’ show a pair of these strains labeled with anti-β-1,3-glucan after 3.5 hr of filamentation (and are similar to an independently engineered pair of such strains that was allowed to filament for an additional 1.5 hr); one strain of each pair had hyphally-expressed *YWP1*, while its negative control had the ectopic *YWP1* disrupted. Hyphal Ywp1 resulted in a decrease in anti-β-1,3-glucan binding, visible by eye as well as in these identically-exposed micrographs. This difference was difficult to quantify because of the variability in signal intensity along the lengths of the filaments, and because these cells were not morphologically amenable to quantitation by flow cytometry. Qualitatively, however, Ywp1 appears to contribute to β-1,3-glucan masking when ectopically present in hyphae, as it does when naturally present in yeast.

To summarize, Ywp1 and β-1,3-glucan of the yeast cell wall normally show little accessibility to antibodies; in strain variant BWP17x, both Ywp1 and β-1,3-glucan show increased accessibility to antibodies through unknown mechanisms that appear to affect the wall structure of this variant. Additional strains have revealed that wall-anchored Ywp1 contributes to the masking of β-1,3-glucan. This adds to the known phenotypic effects of Ywp1 in the cell wall, and highlights the need for improved ways to detect and monitor Ywp1 in the walls of normal cells.

### Gfp as a tag for wall-anchored Ywp1: Utility and limitations

**Fig 7:**
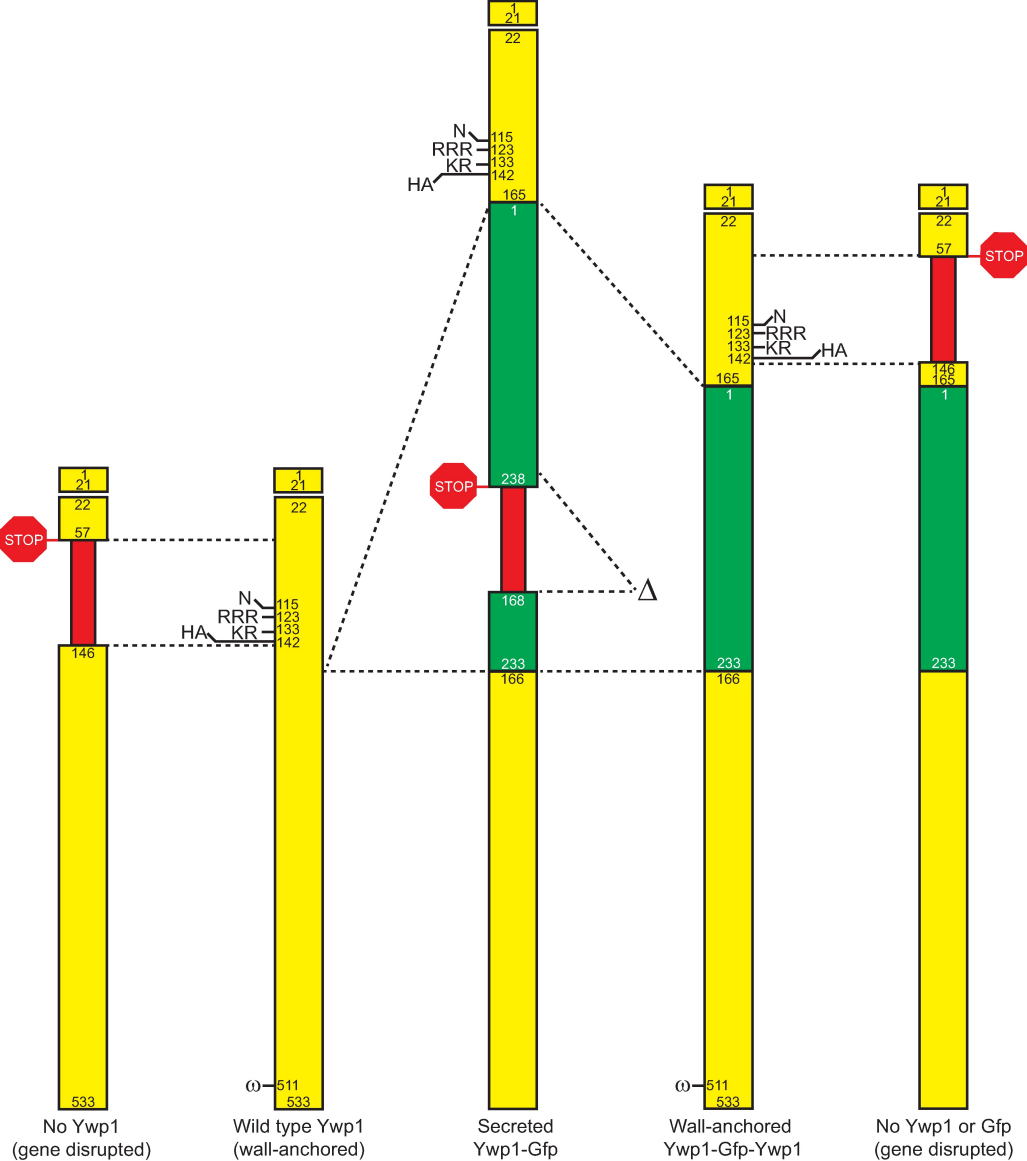
Schematic diagram of Ywp1 and Ywp1-Gfp chimeras. Yellow segments represent Ywp1; green segments represent Gfp; numbers correspond to amino acid positions in the wild type proteins (1–533 for Ywp1 and 1–238 for Gfp). Thinner red segments mark the positions of the selectable *URA3* gene that was inserted into *YWP1* or *GFP* to terminate downstream transcription and/or translation (the latter signified by STOP signs); these segments are not to scale. The Ywp1 signal peptide (aa 1–21) directs the nascent polypeptide into the secretory pathway prior to its cleavage and loss from the mature protein. “N” represents the attachment site of the sole N-glycan of Ywp1. “RRR” and “KR” are the tribasic and dibasic cleavage sites of the Ywp1 propeptide. “HA” shows the position of the hemagglutinin epitope that was present in some constructs (described in subsequent sections). “Δ” shows the *URA3* segment that was looped out and lost by homologous recombination to join upstream and downstream coding regions and delete the C-terminal 5 aa of Gfp. “ω” is the site of cleavage and GPI addition that allows anchorage to the cell wall.

To overcome the hindered accessibility of Ywp1 to antibody probes in typical strains of *C*. *albicans*, Ywp1 was made inherently detectable through the genetic insertion of green fluorescent protein (Gfp). The insertion point was based on previous work [21] that identified a location between domains that did not block secretion or known post-translational modifications of Ywp1. A bifunctional genetic tagging cassette was constructed (S1 File) to allow generation of transformants that would secrete anchor-negative Ywp1-Gfp, and then spontaneously rearrange to give rise to strains that would secrete full-length Ywp1 with Gfp inserted between amino acids 165 and 166 (Fig 7). This created heterozygotes with one allele of *YWP1* encoding wild type Ywp1 and the other allele encoding Ywp1-Gfp-Ywp1 (both of which became wall-anchored after synthesis). Additional experimental and control strains were created by disrupting each of these two alleles with *URA3* (replacing codons 58–145 of *YWP1* with *URA3*), giving a strain with just the wild type Ywp1 as well as a strain with just Ywp1-Gfp-Ywp1 (and no wild type Ywp1) (Fig 7).

Strain BWP17 was transformed in this way, and of the initial 43 transformants, 32 were further analyzed; 23 of those were found to secrete fluorescent Ywp1-Gfp into the culture medium. Growth of four of those transformants in the presence of 5-fluoroorotic acid (5-FOA) selected for loss of *URA3* and homologous joining of the upstream *YWP1-GFP* with the downstream *GFP-YWP1* to generate Ywp1(aa 1–165)-Gfp(aa 1–233)-Ywp1(aa 166–533); three of the four secreters gave rise to strains with cell wall associated Gfp fluorescence. All were confirmed by PCR analysis of their genomic DNA, and one (here termed strain YGY) was used for most of the subsequent characterizations.

**Fig 8:**
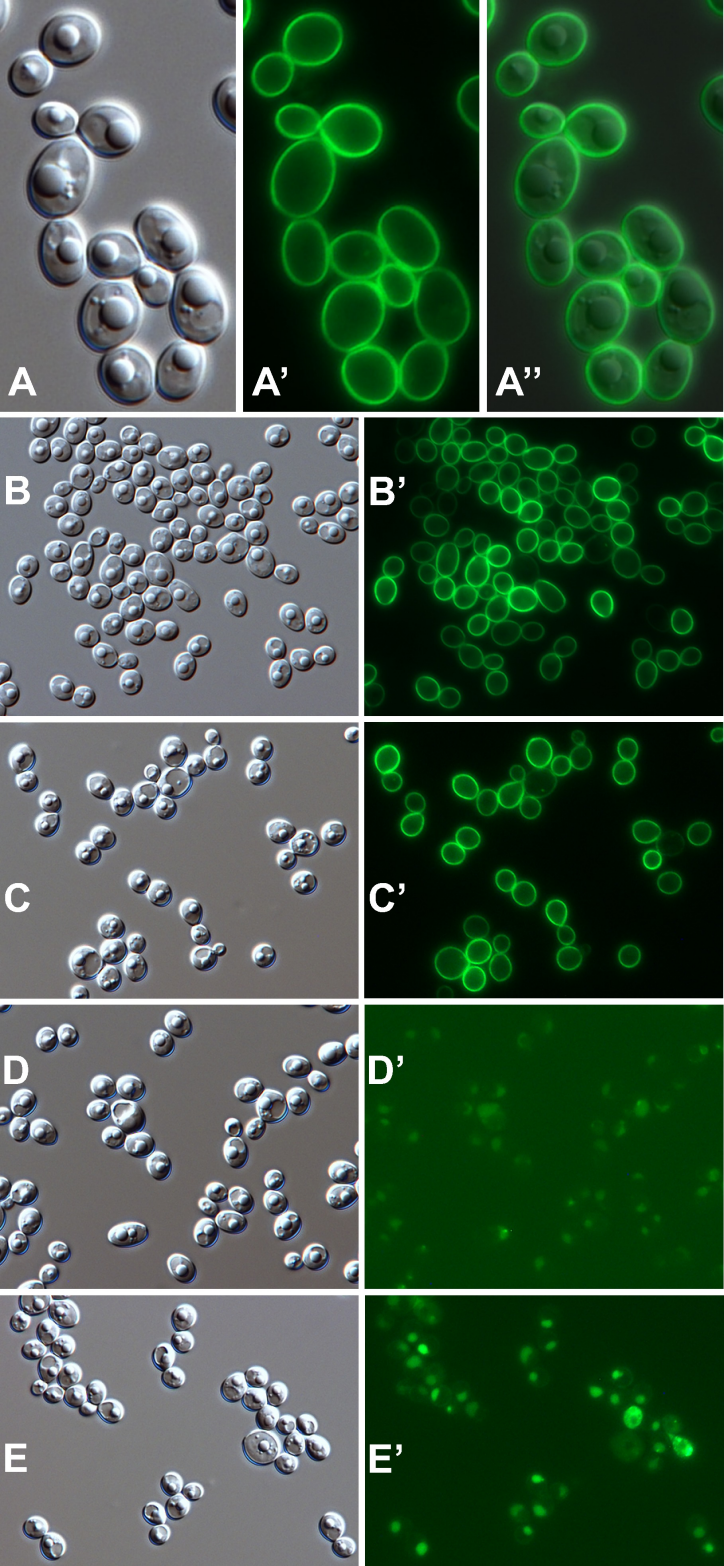
Micrographs of strain YGY and derivatives. DIC images (A-E) and corresponding Gfp fluorescence images (A’-E’). Except as indicated, yeast cells were grown to stationary phase in phosphate-limited BMM13 and dispersed in pH 8 buffer for photomicrography. (A-A”) Overlay shows coincident localization of Gfp fluorescence and the yeast cell wall. (B,B’) Strain YGY; (C,C’) strain YGY derivative with its wild type *YWP1* allele knocked out; (D,D’) strain YGY with its *YWP1-GFP-YWP1* allele knocked out (and 5× greater photographic exposure of fluorescence); (E,E’) same strain as in panels C and C’ but grown in unbuffered MM13 (4× greater photographic exposure). Actual width of each image (μm): A: 24; B-E: 76.

Epifluorescence microscopy showed that the Gfp fluorescence in strain YGY yeast forms was coincident with the cell wall and fairly uniformly distributed (Fig 8A–8A”). There was no indication that the fluorescence extended beyond the visible wall (in fibrils, for example), but the exact position of Gfp within the thickness of the wall could not be resolved. Strain YGY is a heterozygote with one wild type allele of *YWP1*; as expected, disruption of that wild type allele (through insertion of *URA3* near the 5’ end) did not alter the wall fluorescence (Fig 8C and 8C’) relative to the parent strain YGY (Fig 8B and 8B’), whereas disruption of the YGY allele abolished the wall fluorescence (leaving only autofluorescence visible upon 5× greater photographic exposure; Fig 8D and 8D’).

**Fig 9:**
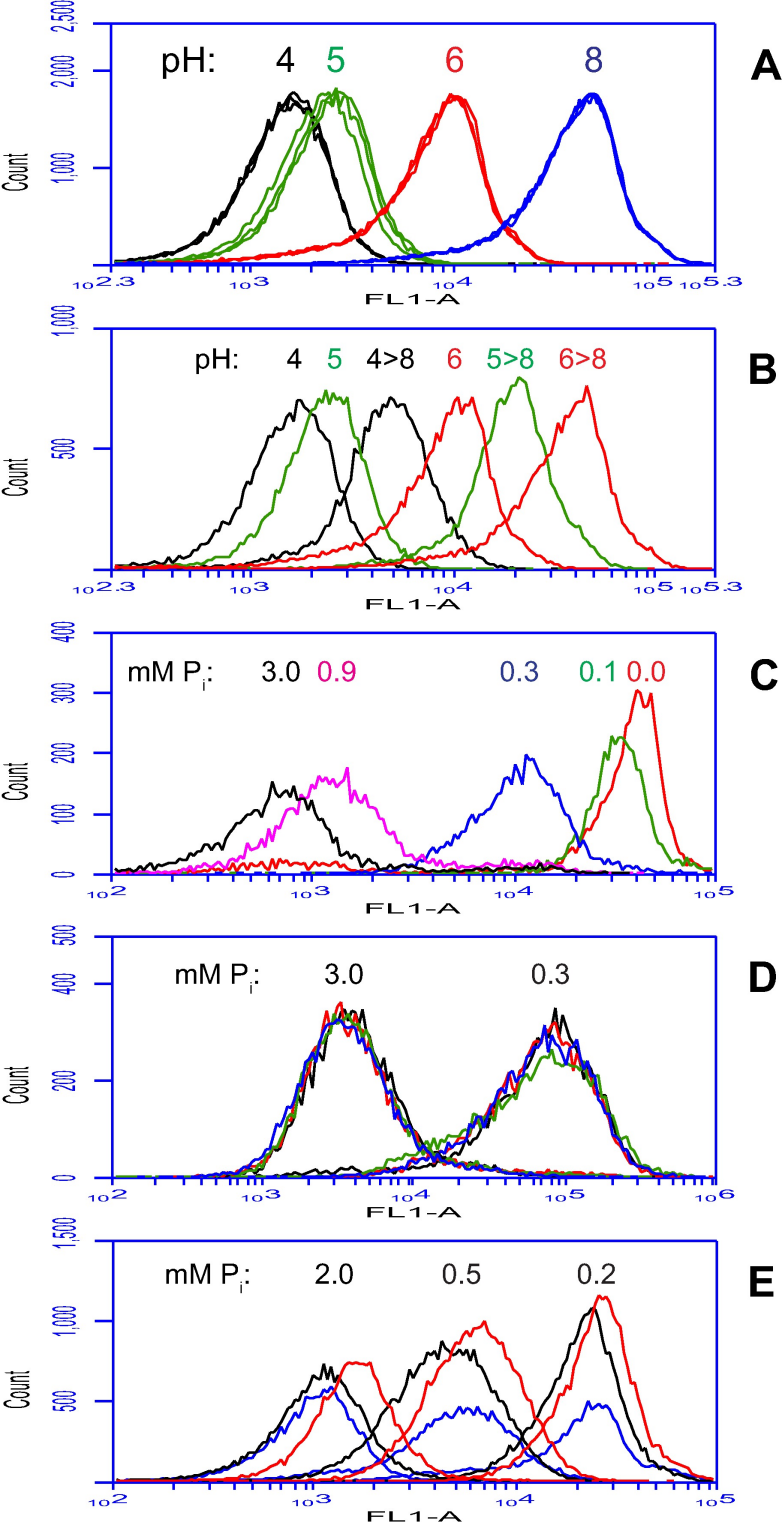
Flow cytometric analysis of strain YGY: Environmental effects on Gfp fluorescence. (A) Yeast forms were grown on low phosphate BMM13 agarose for 4 days and then dispersed in organic buffers at pH 4, 5, 6 or 8 (each supplemented with 0, 20 or 100 mM NaCl) prior to flow cytometry to measure Gfp fluorescence per cell. (B) Aliquots of the above samples (with the 100 mM NaCl supplement) were alkalinized to pH 8 with Tris buffer and analyzed along with the unalkalinized samples; the pH 8 and pH 8→8 samples coincided exactly with the pH 6→8 sample, and were omitted for clarity. (C) Batch liquid BMM13 yeast cultures were started with different amounts of phosphate (3.0, 0.9, 0.3, 0.1 or 0.0 mM), grown to stationary phase at 30°C, and mixed with 50 volumes of 40% ethanol / 50 mM Tris / 10 mM EDTA (pH 8); expression of *YWP1-GFP-YWP1* was enhanced when all of the external phosphate was assimilated (which occurs in BMM13 when the starting concentration is less than 2 mM [22]), resulting in a greater proportion of the population showing induction the sooner the phosphate was depleted. (D) The 3.0 and 0.3 mM phosphate cultures shown in panel C were mixed with 50 volumes of 50 mM Tris / 10 mM EDTA (pH 8; no ethanol) and kept at 23°C, heated to 60°C for 10 min, given 0.5% SDS and kept at 23°C, or given 0.5% SDS and heated to 50°C for 10 min; there was little differential effect on the resulting fluorescence per cell. (E) Cells grown as in panel C with starting phosphate concentrations of 2.0, 0.5 or 0.2 mM were suspended in pH 8 buffer and analyzed live (blue), after heating to 60°C for 15 min (red), or after fixation in 200 mM formaldehyde at 23°C for 90 min (black); the formaldehyde resulted in a slight reduction in fluorescence. Events in panels C and E were gated to eliminate aggregates and multiplets; pre-gate counts (in thousands) were 50 for A and E, 20 for B, and 10 for C and D.

Positioning Gfp in the cell wall subjects it to environmental influences that affect its stability and fluorescence properties. The cultures shown in Fig 8A–8D were buffered at about pH 6; without buffering, the culture pH dropped to around 2, and the fluorescence was lost, even when the cells were examined in a pH 8 buffer (Fig 8E and 8E’). The effect of external pH on the fluorescence of wall-associated Ywp1-Gfp-Ywp1 was more accurately quantified by flow cytometry. Stationary phase yeast cells of strain YGY were suspended in buffer solutions at pH 4, 5, 6 and 8, then analyzed for fluorescence intensity per cell (Fig 9A); the mean and median fluorescence at pH 8 were both 4.8× higher than at pH 6. Each set of peaks in Fig 9A is a composite of 3 samples that contained NaCl at a concentration of 0 mM, 20 mM or 100 mM (in addition to the buffer), which was found to have a minor effect on each profile. These same samples (containing 100 mM NaCl) were then alkalinized to pH 8 with Tris buffer and passed again through the flow cytometer (Fig 9B). The pH 6 sample showed complete restoration of its fluorescence at pH 8; its profile coincided exactly with the pH 8 and pH 8→8 samples (which were omitted from this graph for clarity). The pH 4 and pH 5 samples showed some restoration of fluorescence at pH 8, but much less for pH 4 than for pH 5, presumably because the Gfp had irreversibly denatured over time in these more acidic solutions.

The enhancement of *YWP1* expression upon phosphate limitation also enhanced the amount of Ywp1-Gfp-Ywp1 fluorescence in the cell wall (Fig 9C). When grown in batch liquid cultures of BMM13, cells require at least 2 mM phosphate (starting concentration) to reach stationary phase without assimilating all of the available phosphate [22]; if then placed in phosphate-free BMM13, those cells will divide 2 or 3 times using their stored phosphate. Intermediate starting concentrations of phosphate (0–2 mM) allow proportional amounts of growth prior to external phosphate depletion, *YWP1* induction, and eventual growth cessation. Fig 9C shows this trend with strain YGY cultures that started with 3.0, 0.9, 0.3, 0.1 or 0.0 mM phosphate, with increasing proportions of each population showing enhanced Ywp1-Gfp-Ywp1 production (enhanced Gfp fluorescence) the sooner phosphate was depleted. In this experiment, the median fluorescence per cell at 3.0 and 0.1 mM phosphate starting concentrations differed by a factor of 49. Thus, the accumulation of Ywp1-Gfp-Ywp1 in the cell wall appears to reflect the modulated expression level of *YWP1*.

As shown above, examination and quantitation of wall-anchored Gfp fluorescence was readily performed in live cells. Experimentation with *C*. *albicans* may sometimes require forced cessation of metabolic activity (to prevent cellular changes during the course of an experiment) or killing (to avoid the risk of infection with this potential pathogen), but some of these treatments can be detrimental to Gfp fluorescence. Previous investigations of the resistance of secreted Ywp1-Gfp to harsh conditions [21] were expanded here to explore and optimize fixation conditions for strains with Gfp in their cell walls. Fig 9D shows strain YGY cells that were grown in BMM13 containing excess or limited phosphate; the stationary phase yeast cells were suspended in a pH 8 buffer and kept at 23°C, heated to 60°C for 10 min, given 0.5% SDS and kept at 23°C, or given 0.5% SDS and heated to 50°C for 10 min. For both phosphate regimes, the fluorescence intensity showed little alteration by the heat or SDS treatments. Fig 9E shows a similar experiment using cells from three cultures that were again placed in pH 8 buffers and otherwise untreated, heated to 60°C for 15 min, or fixed with 200 mM formaldehyde for 90 min; again, the overall effects on the fluorescence were small, with the formaldehyde having the most detrimental effect. As for the heat and formaldehyde treatments, buffered 40% (v/v) ethanol was found to rapidly kill all of the cells while leaving most of the Gfp fluorescence intact, although prolonged storage in this solution led to less recoverable fluorescence; in fact, the cells in Fig 9C were fixed with 40% ethanol and applied to the flow cytometer directly in that solution. Thus, a variety of killing/fixation steps may have utility in cases where the preservation of Gfp fluorescence is also desirable.

**Fig 10:**
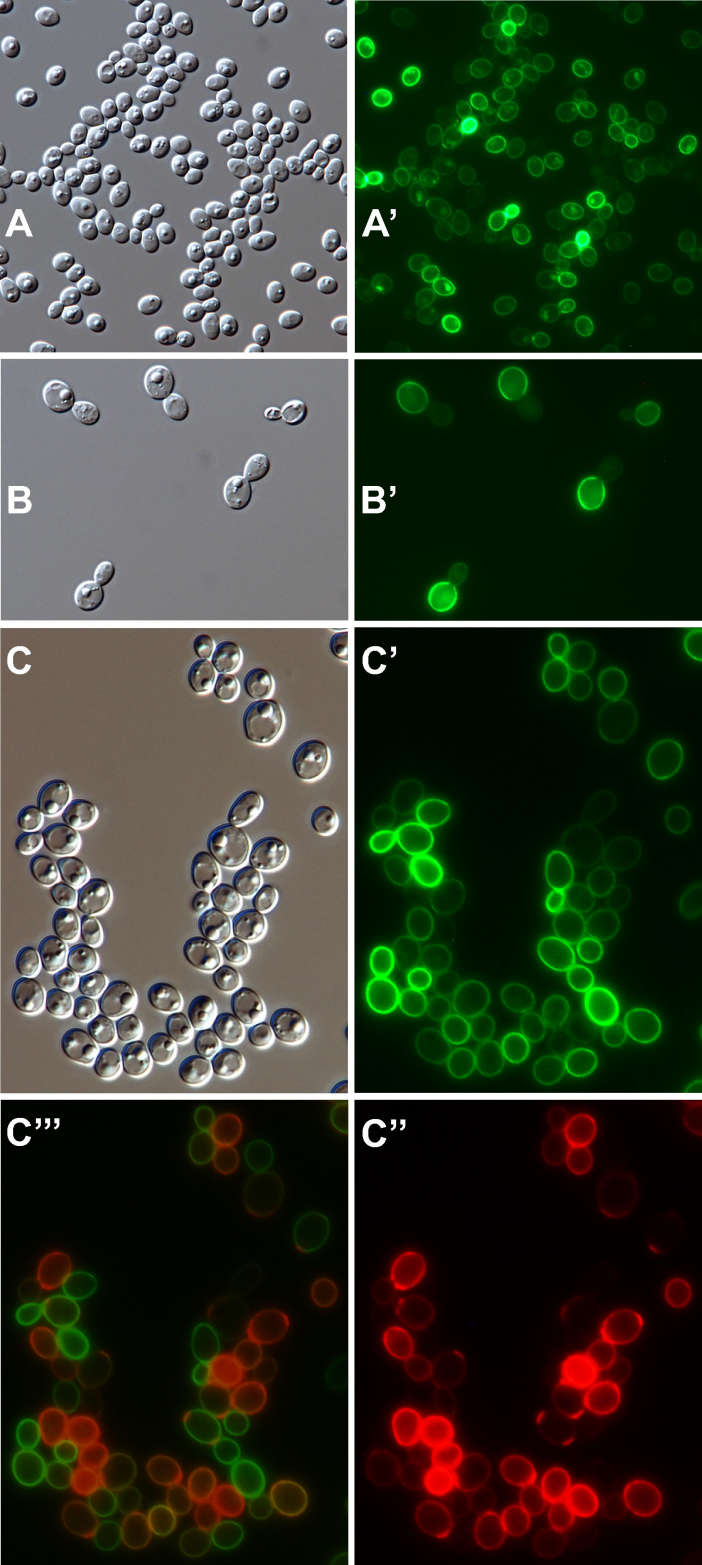
Effects of phosphate limitation on strain YGY. (A,A’) Yeast cells were grown on the surface of rich (phosphate-replete YPD) nutrient agarose; fluorescent Ywp1-Gfp-Ywp1 was often more abundant in the walls of nascent daughters than in their mother cells. When starved of phosphate by growth to stationary phase in low phosphate BMM13, yeast cells accumulated abundant Ywp1-Gfp-Ywp1 in their walls; when placed in fresh medium, these cells budded daughters with less wall fluorescence (B,B’). A range of Ywp1-Gfp-Ywp1 accumulations arose within the population as phosphate was depleted from growing cultures; phosphate depletion also resulted in reduction in the quantity of phosphodiester-linked mannotriose in the cell wall, as shown by immunolabeling with a monoclonal antibody (G11.1) specific for the mannotriose and a fluorescent red (eFluor660) secondary antibody (C-C”‘). Note the frequent complementarity of signal intensities. Note also that the red signal sometimes appears exterior to the green, possibly resulting from a combination of the mannotriose extending farther from the wall and the double-antibody bridge. Actual width of each image (μm): A: 76; B: 76; C: 54.

The cells shown in Fig 8B–8E were grown in phosphate-limited batch cultures, and therefore have relatively strong Gfp signals; the variability in signal intensity among cells presumably reflects the times at which those cells sensed phosphate scarcity and upregulated their Ywp1-Gfp-Ywp1 production, with nascent daughter cells upregulating expression more than mother cells. The Gfp fluorescence intensity may thus serve as a permanent mark of the cell’s age, with the youngest being the brightest under these conditions. Although much less Ywp1-Gfp-Ywp1 is made under phosphate-replete conditions, it is still evident in the cell wall (Fig 10A and 10A’). In these cells that were grown on the surface of rich (YPD) agarose, yeast buds were sometimes brighter than their mother cells; this may be a result of the expression of *YWP1* being greatest during rapid growth [22], consistent with a 3.7× increase in *YWP1* transcript levels in exponential *vs*. post-exponential phase yeast in YPD medium [45], or simply because local phosphate depletion may have occurred in these densely packed cells during growth. When stationary phase cells from phosphate-limited cultures were transferred to a fresh aliquot of the same medium (containing 0.3 mM phosphate), there was more Ywp1-Gfp-Ywp1 fluorescence in the mother cells than in their nascent daughters (Fig 10B and 10B’), presumably because *YWP1* expression was downregulated by the initially-sufficient phosphate concentration. Microscopic analysis thus reveals differences in wall fluorescence intensity that reflect the history of *YWP1* expression for each cell.

Another consequence of phosphate starvation in *C*. *albicans* is reduction in the synthesis of the acid-labile, phosphodiester-linked, β-1,2-mannose chains that are normally abundant components of the outer cell wall mannoproteins [46, 47]. Scarce phosphate is presumably prioritized by growing cells for essential nucleic acid and phospholipid synthesis over optional cell wall mannan elaboration. Alcian Blue is a cationic dye that is commonly used to detect these anionic phosphodiester groups in the cell wall, and phosphate starvation abrogates the binding of this dye, as does deletion of the *MNN4* gene that is necessary for phosphomannan synthesis [48, 49]. A series of monoclonal antibodies specific for β-1,2-mannotriose has been described, and these antibodies bind avidly to the cell wall [34–36]. As predicted, when strain YGY was grown in a phosphate-limited batch culture and then labeled with antibodies specific for the mannotriose (followed by a secondary antibody conjugated to a red fluorochrome), there was considerable complementarity in the red (mannotriose) and green (Gfp) signals (Fig 10C–10C”‘). These images thus confirm that as the culture grew, phosphate depletion resulted in increased Ywp1-Gfp-Ywp1 and decreased phosphodiester-linked mannotriose content in individual cells. This was demonstrated more quantitatively by flow cytometry (S4 Fig). Since reductions in cell wall mannosylphosphate are known to increase cell surface hydrophobicity and adhesivity [49], an increase in the anti-adhesive Ywp1 upon phosphate starvation conceivably counteracts this increase in adhesivity. During phosphate replete growth, Ywp1 may carry a significant fraction of the yeast cell wall phosphomannose [21], possibly enhancing the anti-adhesive effect of Ywp1; whether the upregulation of *YWP1* during phosphate starvation is related to this carriage remains to be explored.

A previous investigation utilized GFP as a reporter of *YWP1* expression by allowing the accumulation of free Gfp in the cytosol of *C*. *albicans* yeast forms [22]. Here, in strain YGY, the Gfp is embedded in Ywp1 and reports expression after traversing the endoplasmic reticulum and secretory pathway in a glycoprotein that has undergone considerable posttranslational modification, suggesting the possibility of different kinetics and fate for this reporter. To compare these two scenarios, strain YGY was compared with a cytosolic Gfp strain, each strain having one of its two *YWP1* alleles modified with *GFP*. Gfp fluorescence intensity per cell was monitored by flow cytometry in batch liquid cultures with surplus or limiting phosphate in a time course from inoculation to stationary phase (S5 Fig). Phosphate starvation resulted in large increases in accumulated Gfp signals that peaked at about 20 hr for the cytosolic Gfp and then declined, but more slowly reached a plateau for the Ywp1-embedded Gfp that was about a third of the intensity of the cytosolic Gfp peak. Fluorescence also declined for cytosolic Gfp relative to wall-anchored Gfp in later stage cultures that had surplus phosphate. Thus, even though the wall-anchored Gfp accumulated in lesser quantities than might be expected from the cytosolic Gfp reporter (perhaps because of difficulties in the folding or translocation of this chimeric construct), it may be more stable in the wall than is the free Gfp in the cytosol.

When strain YGY was grown as individual colonies or as a lawn on low phosphate BMM13 agarose, rare sectors or patches with greater fluorescence intensity were sometimes observed. Several rounds of picking and re-streaking of these brighter cells resulted in stable clonal populations with uniformly greater intensity (here collectively termed strain YGY*). When strains YGY and YGY* were grown in parallel under a variety of conditions (batch cultures of BMM13 with different amounts of phosphate for different times at 30°C) and analyzed by flow cytometry, strain YGY* generally had 3–4× more Gfp fluorescence per cell than strain YGY. PCR analysis of genomic DNA revealed that strain YGY* retained its unaltered copy of *YWP1*, indicating that the remaining wild type allele of *YWP1* was not lost in a gene conversion event; whether the *YWP1-GFP-YWP1* allele was amplified through polyploidy or some other mechanism has not been determined. Regardless, the change resulted in more Ywp1-Gfp-Ywp1 in the cell wall, which increased its ease of detection; strain YGY* otherwise appeared similar to strain YGY, with no obvious phenotypic effects of this overexpression.

**Fig 11:**
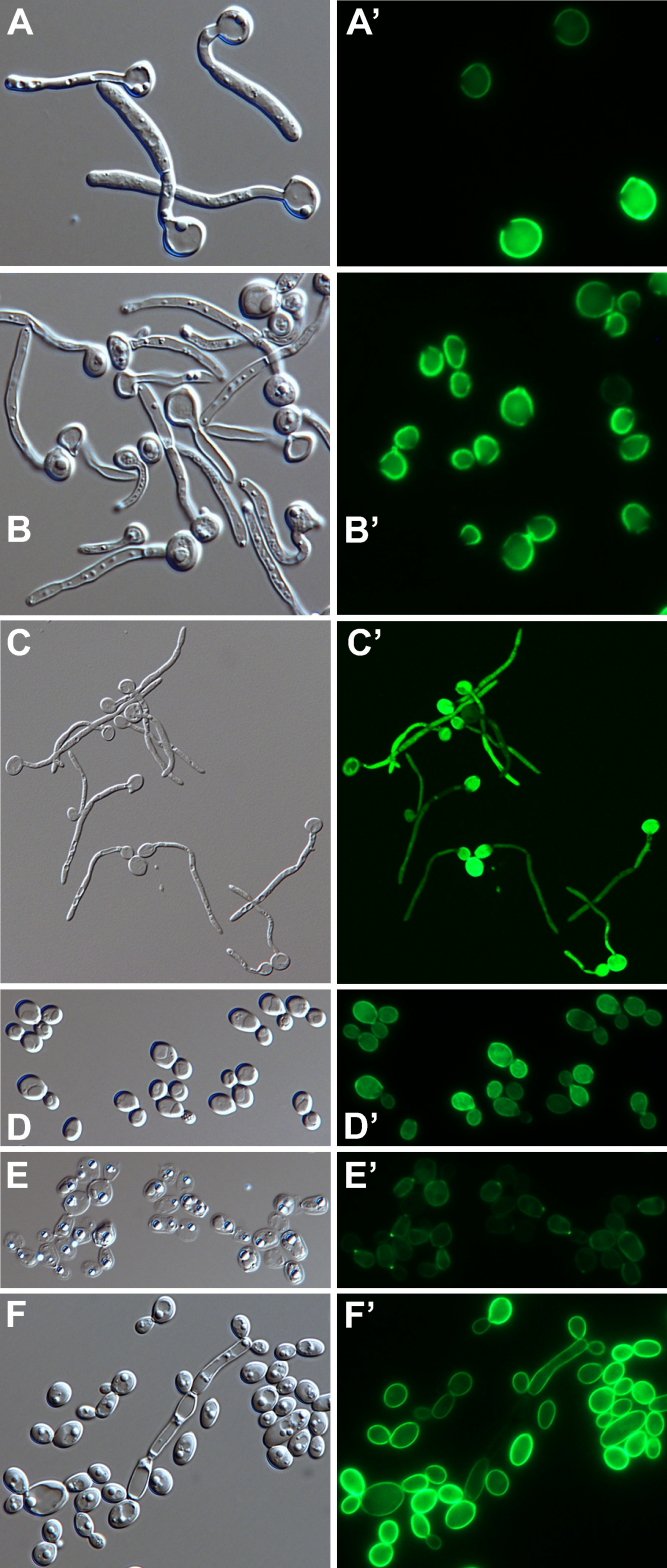
Micrographs of strain YGY*. DIC images (A-F) and corresponding Gfp fluorescence images (A’-F’). Yeast cells were grown to stationary phase in phosphate-limited BMM13. (A-C and A’-C’) Cells were then grown 5 hr in 37°C RPMI filamentation medium; (A,A’) live cells; (B,B’) cells incubated for 30 min in 50°C buffered (pH 8) 1% SDS + 100 mM DTT; (C,C’) live cells mounted in a pH 8 medium containing Tris, EDTA, NaCl, glycerol and polyvinyl alcohol for 10 days at room temperature. Yeast cells were incubated for 30 min in 60°C buffered (pH 8) 2% SDS + 100 mM DTT (D,D’) and subsequently digested with β-1,3-glucanase (8 U Quantazyme™ per 0.2 ml) for 20 hr at 22°C (E,E’). (F,F’) Yeast cells were added to 50 volumes of fresh medium and grown 9 hr at 30°C followed by 21 hr at 35°C to increase pseudohyphal incidence. Actual width of each image (μm): A: 56; B: 56; C: 115; D: 84; E: 98; F: 79.

The downregulation of *YWP1* expression upon filamentation of yeast forms has been well documented (see [21]). No Ywp1-Gfp-Ywp1 was detected in germ tubes of strain YGY* (Fig 11A and 11A’), presumably because of this downregulation, the time required for Gfp to become fluorescent after synthesis, and the requirement for accumulation of enough fluorescent Gfp to be detectable. Ywp1 may thus be actively excluded from germ tubes or simply present at undetectable levels. This result further shows that Ywp1-Gfp-Ywp1 in the yeast cell wall was not redistributed into the germ tube, consistent with its presumed covalent anchorage in the yeast wall. When these cells were heated to 50°C for 30 min in the presence of 1% SDS and 100 mM DTT, the distribution of the Ywp1-Gfp-Ywp1 did not change, consistent with its GPI-linkage to the insoluble glucans of the yeast cell wall (Fig 11B and 11B’). When further growth was stopped and cells were mounted live and unfixed under coverslips, redistribution of some of the fluorescence into the yeast bodies and germ tubes was evident after several days (Fig 11C and 11C’); this presumably resulted from autolysis of the cells and proteolytic release of Gfp from the yeast walls, followed by diffusion throughout the internal compartment. This suggests that some if not all of the Gfp in Ywp1-Gfp-Ywp1 is present at the inner side of the mannoprotein layer of the cell wall, and when proteolytically liberated from its anchor, exits through this barrier slowly if at all. Fig 11D and 11D’ show strain YGY* yeast cells that were extracted with 2% SDS and 100 mM DTT for 30 min at 60°C; flow cytometric analysis indicated that more than half of the Gfp fluorescence remained after this higher temperature treatment, and the micrographs show that it remained in the cell wall. The cells were more easily flattened by the glass coverslip, however, so the narrow focal plane encompassed more of each cell. When these cells were then incubated with β-1,3-glucanase (Quantazyme™ ylg), which dissolves the glucan matrix of the cell wall, the fluorescence was largely lost (Fig 11E and 11E’). Residual fluorescence at bud necks and scars was evident, perhaps because of greater resistance to digestion at these locations. These results thus confirm and document the expected covalent anchorage of Ywp1-Gfp-Ywp1 in the yeast cell wall, similar to the known properties of wild type Ywp1, and also suggest that Ywp1-Gfp-Ywp1 is not all at the outer surface of the wall, such that it would diffuse away from the cell if its anchor were cleaved. This latter observation suggests a deeper positioning of Ywp1 in the cell wall, which may be pertinent to the inaccessibility of Ywp1 to antibodies.

Cultures of YGY and YGY* yeast forms were occasionally found to contain rare pseudohyphal forms with elongated and unseparated daughter cells, wider in diameter than hyphae, and with constricted septae. These cells occasionally but not always had Gfp fluorescence in their cell walls, typically at much lower intensity than in the accompanying yeast walls. Fig 11F and 11F’ show YGY* cells that were grown in phosphate-limiting BMM13 at 35°C rather than 30°C to increase the incidence of pseudohyphal forms, and illustrate that Ywp1-Gfp-Ywp1 is not restricted exclusively to the yeast cell wall, but may also be present at some level in some pseudohyphal walls. The rarity of pseudohyphae in yeast cultures precluded regarding any of them as typical; a more systematic study of pseudohyphal cultures might reveal specific conditions that result in *YWP1* being expressed in morphologies other than the strict yeast form.

### Effect of Gfp insertion on Ywp1 accessibility and other properties

Insertion of Gfp into wall-anchored Ywp1 conceivably alters the relationship of Ywp1 to the cell wall and the accessibility of Ywp1’s epitopes to antibodies. To test this, cells were immunolabeled with the anti-Ywp1 antisera described earlier and a secondary antibody conjugated to a red fluorochrome distinguishable from Gfp. To maximize the quantity of Ywp1-Gfp-Ywp1 in the cell wall, strain YGY* was grown as yeast under phosphate-limited conditions, then fixed with heat or formaldehyde prior to immunolabeling. Anti-Ywp1 (aa 51–197) gave signals indistinguishable from nonspecific background labeling; anti-Ywp1 (aa 1–533) gave slightly stronger labeling, but the signals showed no spatial correspondence to the Gfp fluorescence, suggesting that the bulk of Ywp1-Gfp-Ywp1 was not being detected. Thus, Gfp insertion did not appear to increase the accessibility of Ywp1 epitopes to antibodies. The accessibility of Gfp itself was explored by using three monoclonal antibodies reportedly specific for native Gfp (DSHB-GFP-4C9, -8H11 and -12A6 [50]). In a variety of experiments, strain YGY and YGY* yeast cells were examined after phosphate starvation of cultures and sometimes after growth in 10 nM Caspofungin, after mechanical breakage of the cell walls with glass beads (so that the antibodies could access the inner side of the wall), treatments with SDS at 60°C or 8M urea at 23°C (in the presence or absence of DTT), or fixation with formaldehyde or 40% ethanol (all treatments that had minimal effect on the intrinsic fluorescence of the wall-anchored Ywp1-Gfp-Ywp1). MAb DSHB-GFP-4C9 gave the greatest signals of the three, but those signals were typically quite weak and non-uniform, and showed little colocalization or intensity correspondence with the intrinsic Gfp fluorescence, suggesting that the antibodies were not binding to fluorescent Gfp. In the characterization of these anti-Gfp antibodies [50], no evidence was presented that the antibodies actually bind to fluorescent Gfp, so it is conceivable that the often patchy or punctate signals observed here represent Gfp that had denatured, thus complicating conclusions about the normal accessibility of Ywp1-Gfp-Ywp1 in the cell walls of strains YGY and YGY*. Nevertheless, no evidence was obtained that suggested the Gfp embedded in Ywp1 was more than minimally accessible to externally-applied antibodies.

Anchor-negative Ywp1-Gfp that is secreted into the culture medium has been extensively characterized [21]. The cleaved but tightly associated propeptide of Ywp1 was found to dissociate from purified Ywp1-Gfp in the presence of SDS when the temperature was raised to 50°C; while anchored in the cell wall, however, there was little propeptide dissociation from native Ywp1 at 50°C, but nearly complete dissociation at 70°C. The effect of Gfp insertion into wall-anchored Ywp1 on this dissociation was examined here using a panel of strains (described earlier) that have either or both wild type Ywp1 and Ywp1-Gfp-Ywp1 in their cell walls. Representative results from strain YGY derivatives that have only Ywp1 or only Ywp1-Gfp-Ywp1 in their cell wall are shown in S6 Fig. (These two strains are also shown in Fig 8C and 8D) Small amounts of propeptide were found in the BMM13 culture media of both strains, presumably due to protease-driven shedding [21]. For both strains, extraction of the cells with SDS at 50°C liberated little propeptide, while more was liberated at 70°C, suggesting that Gfp insertion did not affect this property of Ywp1. The greater quantity of propeptide from the wild type Ywp1 is consistent with S5 Fig, for example, which suggests that the quantity of Ywp1-Gfp-Ywp1 that becomes stably incorporated into the cell wall is considerably less than that of wild type Ywp1. Comparison of the propeptide quantities also raises the possibility that Gfp insertion increases the likelihood that Ywp1 will be proteolytically shed into the culture medium. Nevertheless, these data do not reveal alterations to most known characteristics of Ywp1 as a consequence of insertion of Gfp between amino acids 165 and 166.

Finally, the effect of Gfp insertion on the only known phenotypic correlation of Ywp1 (its antiadhesive effect) was investigated in a yeast biofilm spot adhesion assay as described in S3B Fig. Nine strains exhibiting different quantities of wall-anchored Ywp1 and Ywp1-Gfp-Ywp1 were assessed for their yeast adhesiveness (S7 Fig). The results suggest that Ywp1-Gfp-Ywp1 retains an antiadhesive effect; insertion of Gfp into this position thus does not abolish the antiadhesive effect of Ywp1. Dose dependence is also suggested, considering that wall-anchored Ywp1-Gfp-Ywp1 is not as abundant as unmodified Ywp1 (as indicated in S5 and S6 Figs).

### HA-tagged Ywp1

Prior to the above investigations, Ywp1 was genetically tagged with an epitope of influenza hemagglutinin (HA) and found to accumulate uniformly in the yeast cell wall [28]. Specifically, six tandem copies of the HA epitope (having the sequence YPYDVPDYA) interspersed with short linkers were positioned between amino acids 142 and 143 of Ywp1 and detected with an anti-HA monoclonal antibody by immunofluorescence microscopy and Western blotting. The HA-tagged Ywp1 was used primarily to investigate the possibility of proteolytic cleavage of Ywp1 by Sap9 and Sap10.

The HA construct was engineered into *C*. *albicans* strain CAI4 as well as a Δ*sap9* Δ*sap10* derivative; two independent transformants of each were generously provided by Drs. Kasper (formerly Schild) and Hube for further investigation of HA-tagged Ywp1. These strains are here designated “YHY” (as they make wall-anchored Ywp1-HA-Ywp1). Genomic DNA sequencing of the epitope insertion locus revealed the expected 84-codon 6HA cassette sequence in each case, with the following exceptions: In all four strains, a single nucleotide substitution resulted in a cysteine rather than the expected glycine in the linker segment between Ywp1 and the first HA segment, evidently because of a correctly specified but incorrectly synthesized commercial primer used in its construction; the cassette thus encoded the underlined sequence *DQIDDFIAS*GAGAGASCSAAR●G●GS●SR●G●GS●SSTSGAGAGA*IENTEGTALEGSTLEVVDYVSGS*#, where ● represents the HA epitope YPYDVPDYA, the adjacent Ywp1 amino acids are *italicized* (and start with the N-terminus of Ywp1 that is generated by propeptide cleavage), and # represents the Gfp insertion point of the constructs described earlier in this report. Also, in one of the two Δ*sap9*,*10* transformants, 32 codons were missing from 6HA, which converted it to 3HA (…AAR●G●GS●SST…), presumably through recombinatorial loss of half of the tandem repeats. (The HA epitope is encoded by three distinct nucleotide sequences that make use of alternative synonymous codons, and 6HA is a direct repeat of the 3HA epitope assembly.)

By immunofluorescence microscopy after a variety of fixation and preparation conditions, Ywp1-6HA-Ywp1 and Ywp1-3HA-Ywp1 appeared to be localized in the yeast cell wall, but with a signal that was not as intense as anticipated, considering the relative abundance of Ywp1. A small proportion of the cells (fewer than one in a thousand), however, gave a far stronger signal with anti-HA. Starting with both wild type and Δ*sap9*,*10* strains expressing *YWP1-6HA-YWP1*, multiple rounds of subdivision and screening eventually resulted in clonal populations in which every cell gave the stronger anti-HA signal (here designated as strains YHYx). A YHYx version of the Ywp1-3HA-Ywp1 strain was not isolated. Interestingly, genetic analysis revealed that both YHYx strains had lost their wild type *YWP1* locus, presumably through gene conversion events that replaced it with the *YWP1-6HA-YWP1* version. This would be expected to double the signal for Ywp1-6HA-Ywp1 in the cell wall, but the signal appeared to be far more than doubled in the YHYx strains.

**Fig 12:**
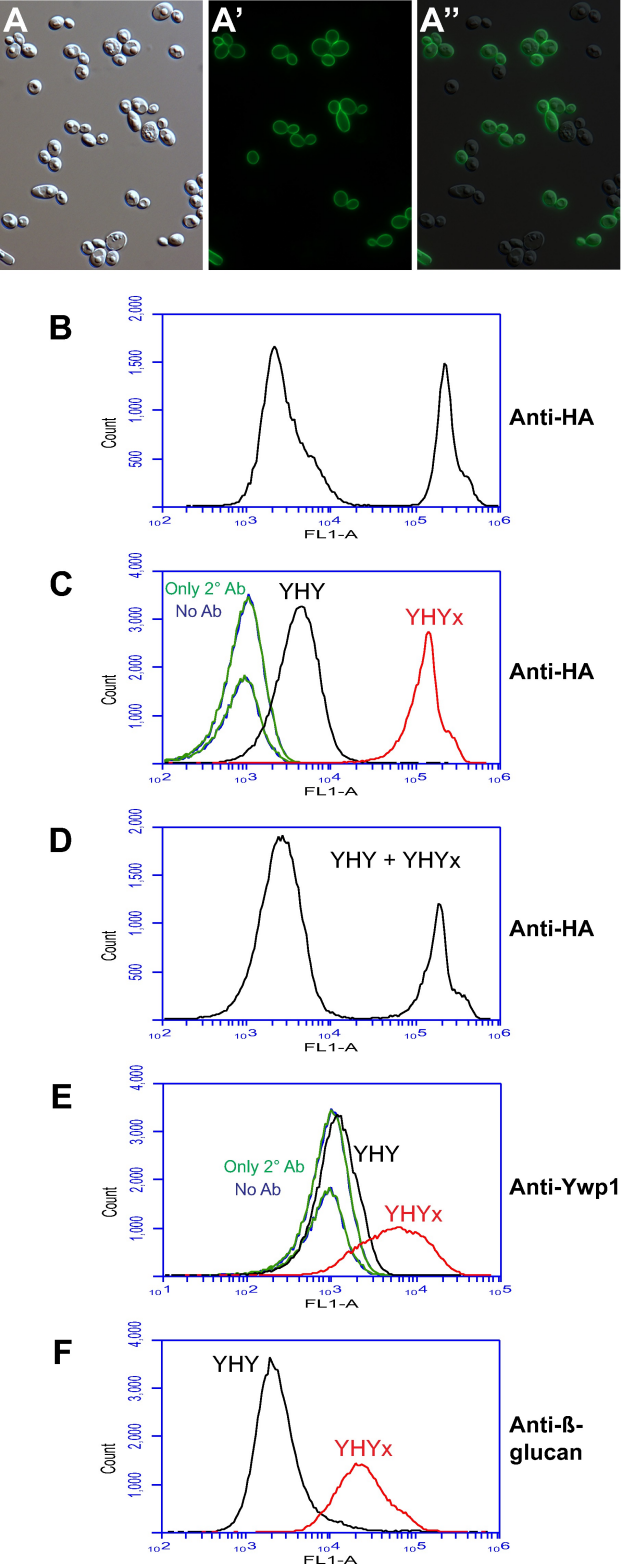
Epitope accessibility in strains YHY and YHYx, both of which have wall-anchored Ywp1-6HA-Ywp1. All cultures were grown 45 hr to stationary phase in 30°C BMM13, fixed with formaldehyde, and immunolabeled with anti-HA mAb (A’,A”,B,C,D), anti-Ywp1 (aa 1–533) antiserum (E), or anti-β-1,3-glucan mAb (F), each followed by fluorescein-conjugated secondary (2°) antibody. (A-A”) Micrographs of a pre-clonal intermediary population with similar numbers of YHY and YHYx cells; at this exposure, the anti-HA signal from the cell walls is visible only in the YHYx cells. (B) Corresponding flow cytometric analysis of the sample in (A); the peaks differ by a factor of 77 (mean) or 94 (median) in intensity. (C-F) Clonal populations of strain YHY and YHYx were immunolabeled separately or as a mixture (D only) with the indicated primary antibody. In D, the peaks differ by a factor of 71 (mean) or 76 (median) in intensity; in E and F, the difference is 8–10×. Profiles are color-matched to their labels.

Fig 12A–12A” show immunofluorescence localization of Ywp1-6HA-Ywp1 in a pre-clonal intermediary population that had similar proportions of YHY and YHYx cells; the signal localized to the cell wall of all of the cells, but at the exposure shown here is only visible in the YHYx cells. Fig 12B shows the corresponding flow cytometric analysis of this sample, with two peaks that differ in fluorescence intensity by a factor of 77 for their means and 94 for their medians. Comparisons of final clonal populations of YHY and YHYx are shown in Fig 12C–12F. The two strains were labeled with no antibodies, secondary (fluorescein-conjugated) antibody alone, or anti-HA plus secondary antibody (Fig 12C); mixing the two strains before antibody application (Fig 12D) allowed better quantitation of relative binding, and in this experiment showed 71× (mean) and 76× (median) more signal from YHYx. The same formaldehyde-fixed cell preparations showed 8–9× more binding of anti-Ywp1 (aa 1–533) to YHYx than to YHY (Fig 12E), and a similar increase in binding of anti-β-glucan (Fig 12F). Thus, the presumed doubling of the quantity of Ywp1-6HA-Ywp1 in the cell wall of strain YHYx relative to strain YHY cannot account for the much greater increases in anti-HA and anti-Ywp1 binding, nor the increase in anti-β-glucan binding. The difference between strains YHY and YHYx more resembles the difference between strains BWP17c and BWP17x described earlier, possibly reflecting other differences in their wall structures. Consistent with this possibility are the observations that strain YHYx is more sensitive than strain YHY to Calcofluor White, Congo Red and Caspofungin (S8A Fig), and strain YHYx is more adhesive than strain YHY (S8B Fig); this latter property, however, might also be a consequence of Ywp1-6HA-Ywp1 lacking the antiadhesive effect of wild type Ywp1 (a possibility explored below). The phenotypic characterization of the YHYx strain that was also *Δsap9*,*10* was not pursued further because of the difficulty of attributing changed traits to specific mutations.

At its described insertion point, the HA tag did not prevent accumulation of Ywp1-6HA-Ywp1 in the cell wall, but it did alter some molecular characteristics of Ywp1. Whereas the cleaved propeptide of wild type Ywp1 was extractable from cells by SDS at 70°C but poorly at 50°C (as in S2 Fig), all of the available cleaved propeptide from Ywp1-6HA-Ywp1 was extracted at 50°C, leaving none for the subsequent 70°C extraction (S8C Fig). In addition, more total protein was extracted by hot SDS from YHYx than from YHY cells, also reminiscent of a difference between strains BWP17c and BWP17x (see S2 Fig). Even though the total quantity of SDS-extractable propeptide from Ywp1-6HA-Ywp1 was much less than that from wild type Ywp1, there was no way to determine whether this was due to a lack of cleavage of Ywp1-6HA-Ywp1 to create the propeptide, or simply a lower quantity of accumulated Ywp1-6HA-Ywp1 in the cell wall. Therefore, to further explore the molecular consequences of the HA insertion, the YHY and YHYx strains were additionally modified by insertion of a *GFP-URA3* cassette described previously [21, 22] to generate strains that secreted Ywp1-HA-Gfp. Also, the bifunctional *GFP-URA3-GFP* cassette described above (Fig 7) was inserted to generate strains that secreted Ywp1-HA-Gfp as well as give rise to strains with doubly-tagged Ywp1-HA-Gfp-Ywp1 anchored in their cell walls.

#### Comparison of secreted versions of HA-tagged and Gfp-tagged Ywp1

Transfection of these two cassettes into *YWP1 / YWP1-HA* strains resulted in transformants with *GFP* inserted into either the *YWP1* allele or the *YWP1-HA* allele; the former strains all secreted into the culture medium Ywp1-Gfp molecules with properties the same as those characterized previously [21], while the latter strains all secreted Ywp1-HA-Gfp with unexpected properties, as detailed in the next paragraph. PCR and sequence analysis of the genomic DNA of these strains confirmed the expected changes in all cases but one: One of the several transformants of strain YHYx (*YWP1-6HA-YWP1 / YWP1-6HA-YWP1*) had deleted 3 of the 6 HA segments from the allele that had integrated the *GFP* cassette, so that it secreted Ywp1-3HA-Gfp, and had Ywp1-6HA-Ywp1 anchored in its cell wall. This *YWP1-3HA-GFP-YWP1* allele also had a point mutation that changed a proline codon to a serine codon in *YWP1* just upstream from the *GFP* insertion site (thus encoding …DYVSGS-Gfp rather than …DYVPGS-Gfp). Comparing paired transformants, fluorometric quantitation of stationary phase culture supernatants revealed the presence of only about half as much secreted Ywp1-6HA-Gfp as Ywp1-Gfp, and an intermediate amount of Ywp1-3HA-Gfp; this suggested that the inserted HA assembly interfered somewhat with the synthesis, folding, processing, transport or secretion of Ywp1-Gfp, with the 6HA assembly interfering more than the 3HA assembly. A more significant difference became evident, however, when the secreted molecules were resolved by SDS-PAGE.

**Fig 13:**
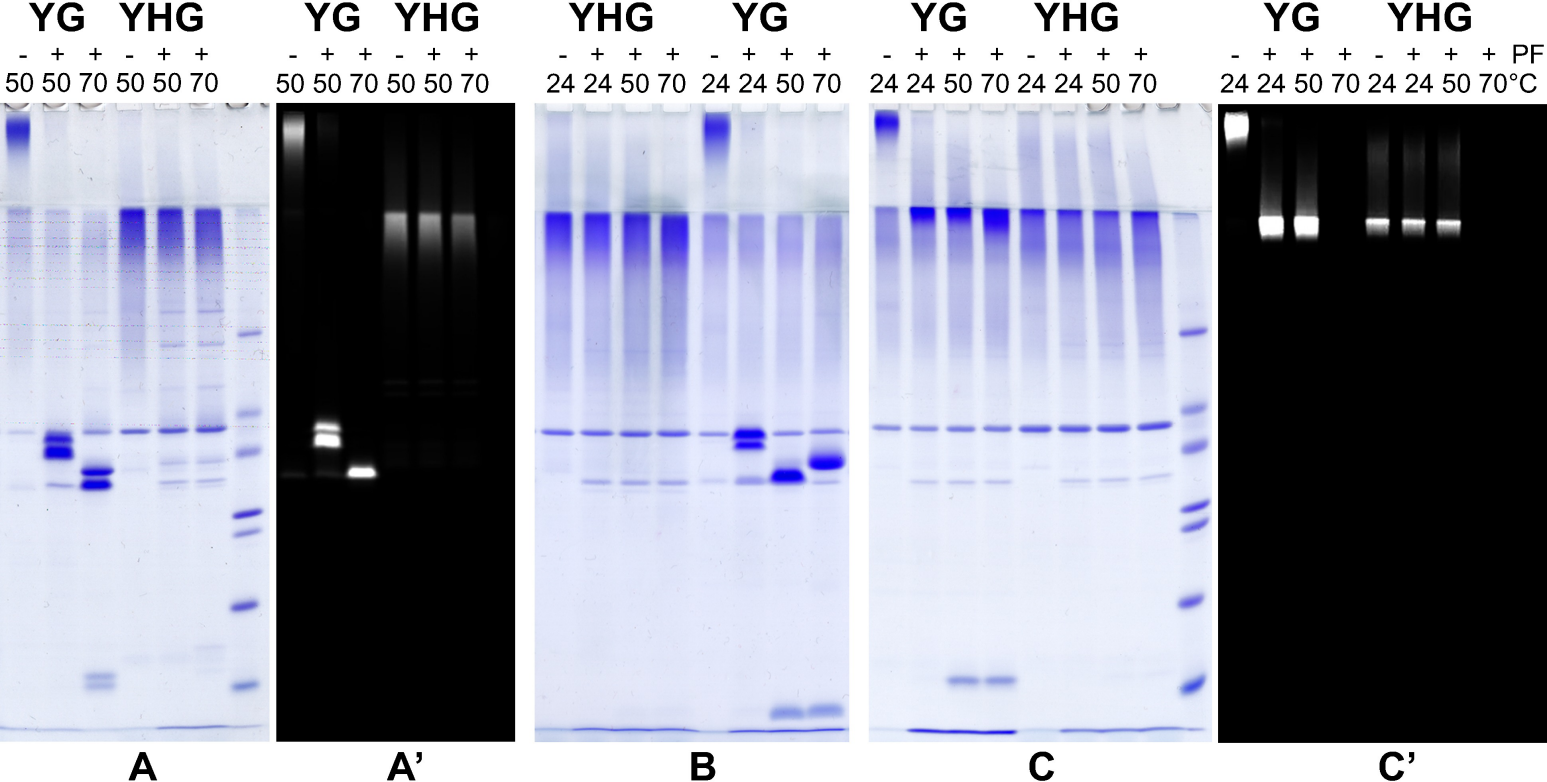
Comparison of the properties of secreted Ywp1-Gfp and Ywp1-6HA-Gfp. Strain YHY was transfected with a cassette that inserted *GFP* into either its *YWP1* allele or its *YWP1-6HA-YWP1* allele, generating comparator strains that secreted either Ywp1-Gfp (“YG”) or Ywp1-6HA-Gfp (“YHG”), as indicated. The Gfp was fused to either aa 165 (A,B) or to aa 520 (C) of Ywp1. Cultures were grown for 45–47 hr in 30°C BMM13 that started with 0.3 mM phosphate. After alkalinization of the culture supernatants to pH 8, secreted proteins were concentrated by centrifugal ultrafiltration. Aliquots were heated to the indicated temperatures (24, 50 or 70°C) before (A) or after (B,C) digestion (or mock digestion) with PNGase F (“PF”, which removes the N-glycan from Ywp1 aa 115). Digested (+) and undigested (-) samples were mixed with excess SDS (and DTT for A and C) and incubated at 24°C (A) or at the indicated temperatures (B and C) prior to SDS-PAGE, which was followed by laser scanning for Gfp fluorescence (A’,C’) and subsequent staining for protein with Coomassie Blue R-250 (A,B,C). In the presence of excess SDS (B,C), heating to 70°C denatured the Gfp (and rendered it nonfluorescent); in the absence of excess SDS (A), denaturation by 70°C was much less efficient. Markers in A and C are as in S2 Fig. The Ywp1 propeptide bands at the bottom of the gels exhibit slight degradation in an older sample (A) and greater mobility without disulfide reduction (B), as noted previously [21, 22].

Fig 13 shows soluble Ywp1-Gfp and Ywp1-6HA-Gfp molecules that were concentrated from culture supernatants by ultrafiltration, then digested (or mock digested) with PNGase F prior to resolution by SDS-PAGE. As documented previously for secreted “Ywp165-Gfp” (aa 1–165 of Ywp1 coupled to aa 1–238 of Gfp [21]), the intact molecule barely migrated into the stacking gel, but removal of its large N-glycan allowed it to migrate to the middle of the resolving gel (Fig 13A and 13B, “YG” lanes). Heating to 50°C did not dissociate the Ywp1 propeptide from the Ywp1-Gfp core (Fig 13A) unless SDS was present in excess (Fig 13B). Heating to 70°C denatured some (or all, if SDS was present in excess) of the Gfp, rendered it nonfluorescent, and altered its mobility (Fig 13A and 13B). The propeptide was visible as a sharp band when deglycosylated and dissociated from the Ywp1-Gfp core. In contrast, the intact Ywp1-6HA-Gfp molecules all migrated through the stacking gel but barely into the resolving gel (Fig 13A and 13B, “YHG” lanes). Coupled with the observation that digestion with PNGase F did not alter their mobility, this suggests that the Ywp1-6HA-Gfp molecules did not acquire the large N-glycan of Ywp1-Gfp. In addition, little if any propeptide was evident after heating in the presence of SDS, suggesting that little if any cleavage occurred in Ywp1-6HA-Gfp to create a propeptide. The lower levels of propeptide extracted from intact YHYx cells with hot SDS (S8C Fig) may thus be due in part to a lack of cleavage to create the propeptide. Most puzzling, however, was the observation that the Ywp1-6HA-Gfp molecules migrated as heterogeneous smears and remained near the top of the resolving gel under all conditions that were tested. With signal peptide removal but without propeptide cleavage or glycosylation, the Ywp1-6HA-Gfp polypeptide should have a mass of 51,566 Daltons and migrate to the middle of the resolving gel as a sharp band, yet none of the following treatments altered the unexpected gel position of Ywp1-6HA-Gfp: 1% SDS (with or without 100 mM DTT) at any temperature up to 95°C; alternative detergents CHAPS or Empigen-BB; 10 M urea; or precipitation by ethanol at 50–65%. (All of these treatments except SDS above 50°C were compatible with Gfp fluorescence, which was used in conjunction with Coomassie Blue staining to confirm the position of Ywp1-HA-Gfp in the gel.) The Ywp1-3HA-Gfp molecule behaved similarly, but migrated slightly further into the resolving gel. These molecules thus appeared to aggregate or acquire an anomalous conformation that did not allow their resolution by SDS-PAGE, and this appeared to be attributable to the HA inserts.

To determine whether the close proximity of the HA and Gfp tags to each other (separated by only 22 aa of Ywp1) was problematic, Gfp was alternatively inserted near the C-terminus of Ywp1 (at aa 520). As described previously [21], the resulting “Ywp520-Gfp” (Ywp1 aa 1–520 coupled to Gfp aa 2–238) barely migrated into the stacking gel unless the N-glycan was removed; the large, O-glycosylated core of Ywp1, however, allowed only slight penetration into the resolving gel even after N-glycan removal (Fig 13C), so this molecule was less useful for delineating HA-induced mobility shifts. Nevertheless, the lack of a mobility shift by PNGase F and the absence of a detectable deglycosylated propeptide again indicated deficient N-glycosylation and propeptide cleavage in this version of Ywp1-6HA-Gfp that physically separated the two tags by an additional 355 aa.

The amino acid sequence and composition of the HA inserts themselves were further explored as possible causes of the anomalous mobility of Ywp1-HA-Gfp. With two arginines and 12 aspartates, the 6HA cassette added a net charge of minus 10 (minus 5 for 3HA), which might have affected the electrophoretic mobility of the polypeptides in both the presence and absence of SDS, but this would not be expected to produce low-mobility smears. Despite an abundance of both nonpolar and charged amino acids, the HA assembly seems unlikely to have a tendency to strongly aggregate with itself through hydrophobic or ionic interactions, but this possibility has not been explored experimentally. The unplanned, unpaired cysteines in each insert might have resulted in dimerization through disulfide bonding, but only if those disulfides were not reducible by DTT, even under strongly denaturing conditions. The inserted 6HA and 3HA assemblies may have enhanced susceptibility to digestion by secreted acid proteases (S9 and S10 Figs), but this would also not be expected to have produced low-mobility smears during SDS-PAGE. The linkers in 6HA added 8 serines and one threonine, and these are potential O-glycosylation sites. The corresponding Ywp1-Gfp without any HA insert (“Ywp165-Gfp”) has 3 serines and 3 threonines in the core Ywp1 portion, but there is no evidence that these or any other serines or threonines in this chimera are glycosylated [21]; the 3HA and 6HA cassettes, however, both added an -SSTS- segment that some predictive algorithms suggest is much more likely to be glycosylated than shorter S/T segments [51]. Fungal O-glycans on secreted proteins tend to be short, linear, α-linked mannose chains, and as such would not be expected to drastically alter the mobility of the underlying polypeptide during SDS-PAGE, even though large numbers of these chains presumably collectively reduce the mobility of “Ywp520-Gfp” [21]. To investigate this further, enzymatic removal of possible O-glycans from Ywp1-HA-Gfp with jack bean α-(1–2,3,6)-mannosidase was attempted. This enzyme was recently shown to be superior for removal of yeast O-glycans [52]. Testing many different treatments and digestion conditions, there has been no obvious effect of the mannosidase on the mobility of the Ywp1-HA-Gfp molecules (S10 Fig), suggesting that O-glycans, if present, are not responsible for the diminished, smeared mobility of Ywp1-HA-Gfp. Thus, the mobility anomaly of Ywp1-HA-Ywp1 may not be attributable to the HA insert directly, but may be a secondary effect of the lack of N-glycosylation or propeptide cleavage of Ywp1. Either way, it is not yet known whether any of these features are relevant to the biosynthesis or properties of wall-anchored Ywp1-HA-Ywp1; the wall-anchored distribution of Ywp1-6HA-Ywp1, however, appears to be the same as that of Ywp1-Gfp-Ywp1, as described below.

#### Wall-anchored versions of Ywp1 with dual HA and Gfp tags

For the YHY and YHYx strains that were transformed with the bifunctional *GFP-URA3-GFP* cassette, recombinative loss of the *URA3* and joining of the flanking *GFP* segments allowed production of wall-anchored Ywp1-HA-GFP-Ywp1. No Ywp1 / Ywp1-6HA-Gfp-Ywp1 strain emerged from this process, but the following strains were successfully produced and studied: Ywp1-Gfp-Ywp1 / Ywp1-6HA-Ywp1 (from strain YHY) and Ywp1-6HA-Gfp-Ywp1 / Ywp1-6HA-Ywp1 (from strain YHYx), as well as these two alternatives from a YHY strain that was also *Δsap9*,*10*: Ywp1-Gfp-Ywp1 / Ywp1-3HA-Ywp1 and Ywp1 / Ywp1-3HA-Gfp-Ywp1. The latter is the only strain that had HA exclusively in the same Ywp1 molecule as Gfp, and was thus useful for having the entirety of each tag signal attributable to the same molecule.

**Fig 14:**
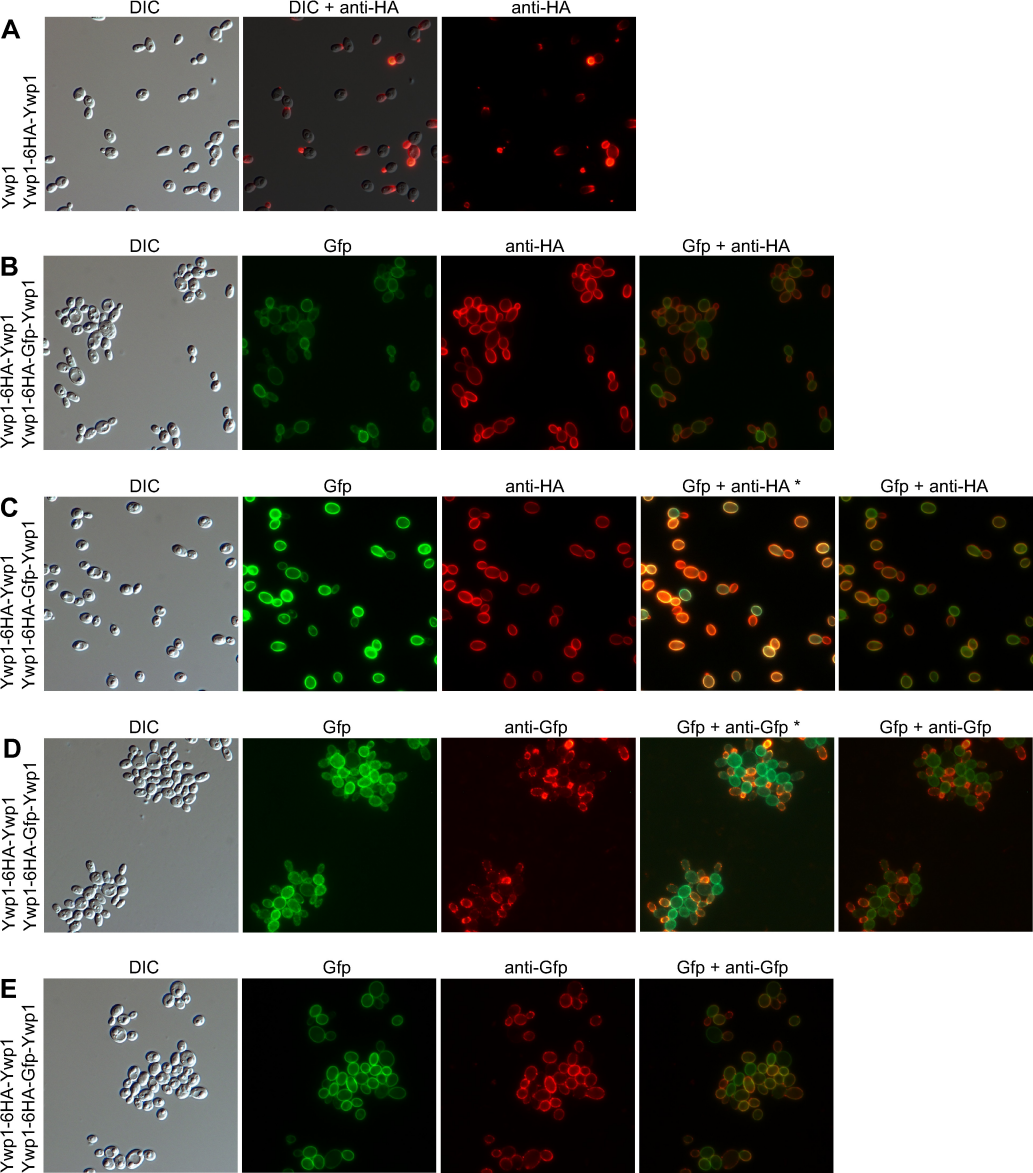
Detection and localization of Gfp and HA tags in Ywp1 (part 1). Micrographs in each row show corresponding DIC images, intrinsic Gfp fluorescence (Gfp), antibody-amplified epitope fluorescence (red anti-HA and anti-Gfp), and digital overlays of the green and red images; in some cases (*), both the red and green fluorescence were photographically captured in the same image. The actual width of each image is 88 μm. Cells were grown in 30°C BMM13 for 20 hr, then transferred to 30°C BMM13 lacking phosphate for 5 hr (A,B,D) or 23 hr (C,E) prior to formaldehyde fixation and labeling with monoclonal primary antibody (anti-HA or anti-Gfp) and AlexaFluor 568 conjugated secondary antibody. The versions of Ywp1 present in each strain are shown at the left of each row; row A is strain YHY and rows B-E are a derivative of strain YHYx.

**Fig 15:**
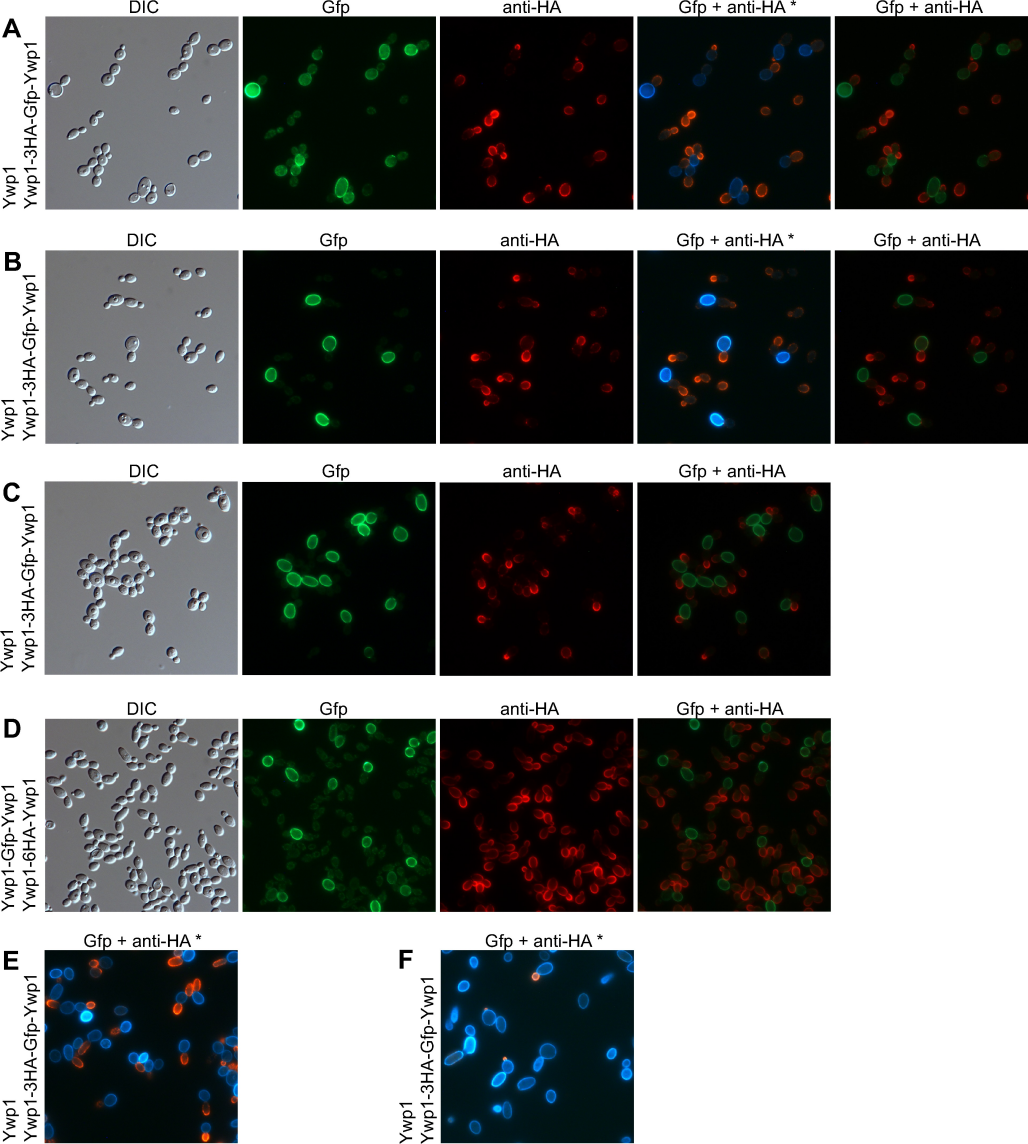
Detection and localization of Gfp and HA tags in Ywp1 (part 2). See Fig 14 legend for an explanation of the labels. The actual width of each image is 88 μm. (A-D) Cells were grown to stationary phase in 30°C BMM13 with (A) or without (B-D) phosphate and then grown for 5 hr in 30°C YPD prior to fixation and antibody labeling. (E,F) Cells were grown for 4 hr (E) or 22 hr (F) in 30°C BMM13 with (F) or without (E) phosphate prior to fixation and antibody labeling as in Fig 14. All cells were fixed with formaldehyde except those in row C, which were fixed with heat (60°C for 15 min). Two strains are shown, both derived from YHY strains; cells in all rows but D were also *Δsap9*,*10*.

When Gfp-tagged Ywp1 and HA-tagged Ywp1 were present as separate molecules in the same cell, they both accumulated in the yeast cell wall, as shown above for cells having just one of the two tagged versions of Ywp1; similarly, doubly-tagged versions (with both Gfp and HA present in the same Ywp1 molecule) also accumulated in the yeast cell wall (Figs 14 and 15), indicating that the double tag did not prevent synthesis or transport to this final destination. Strikingly, however, in conjunction with the previously described changes in epitope accessibility during budding and growth, these strains all clearly illustrated two important aspects of these tags: The difference between *presence* and *accessibility*, and the inherent quantitative differences in detectability between fluorescent protein tags and epitope tags. Whereas detection of Gfp through its intrinsic fluorescence is limited to the signal emitted by the molecule itself, detection of epitopes with fluorochrome-conjugated antibodies can benefit greatly from signal amplification. In the present cases, a monoclonal antibody bound univalently to a Gfp epitope or HA epitope, and that primary antibody was then bound by multiple secondary antibodies, each multiply labeled with fluorescent molecules; in addition, the HA tag had 3 or 6 copies of the HA epitope, further amplifying the signal from each tagged Ywp1 molecule. This large amplification in epitope signals helped to illustrate the difference between presence and accessibility, as documented in the following paragraphs.

In Fig 14, cells were switched from phosphate-replete to phosphate-free growth medium (to enhance *YWP1* expression) and grown for 5 hr or 23 hr prior to formaldehyde fixation and immunolabeling. Anti-HA labeling of strain YHY (containing just Ywp1-6HA-Ywp1) showed most of the signal in nascent buds and necks, with little detectable signal in the more mature cells (Fig 14A). Anti-HA labeling of a derivative of strain YHYx showed more signal in the mature cells (Fig 14B and 14C), as documented previously for strain YHYx (Fig 12), presumably because of greater accessibility of the HA epitope in this strain. This derivative also had *GFP* inserted into one of the two alleles of *YWP1-6HA-YWP1*, so nominally half of the Ywp1 molecules that were produced were doubly-tagged (with HA and Gfp). The accumulated Gfp in the cell wall was greater at 23 hr than at 5 hr because of the induction of *YWP1* by phosphate starvation. Complementarity in Gfp *vs* anti-HA signal intensity between mother and daughter cells was often evident, although for this strain there were more HA tags than Gfp tags in each cell because of the additional presence of the singly-tagged HA-Ywp1. Anti-Gfp provided a more direct demonstration of the difference between presence and accessibility, however, since the mother-daughter complementarity in Gfp *vs* anti-Gfp signal intensities was still evident when both signals were reporting the same Gfp molecules (Fig 14D and 14E). In some preparations, the intensity of the Gfp fluorescence was similar to the intensity of the Gfp epitope or HA epitope (as amplified by the fluorescent antibodies), allowing documentation of both the green (Gfp) and red (antibody) fluorescence in a single photographic image (which closely matched the digital overlays of the separate photographs). More often, disparate exposure times allowed only the digital overlays. These images thus illustrate the presence of Gfp and HA throughout the yeast cell wall, but the limited accessibility of their epitopes to externally-applied antibodies, and a decline in that accessibility as the cells grew and matured.

As documented by flow cytometry for strain YHYx relative to strain YHY (Fig 12E), enhanced binding of anti-Ywp1 was also observed for the Gfp-tagged YHYx derivative, especially to buds. Interestingly, the binding of the anti-Ywp1 (aa 51–197) was enhanced as well as that of the anti-Ywp1 (aa 1–533), with or without the additional Gfp tag, raising the possibility that the inserted HA tag may have increased the exposure or accessibility of some Ywp1 epitopes. Further exploration and documentation of this possibility awaits development of monoclonal antibodies against Ywp1.

Fig 15 shows anti-HA labeling of a strain in which the Gfp and HA tags are exclusively in the same Ywp1 molecules (Fig 15A, 15B, 15C, 15E and 15F), as well as, for comparison, a strain in which they are in separate Ywp1 molecules (Fig 15D). Both strains were derived from strain YHY, and therefore have relatively poor accessibility of the HA epitope in mature cells. The cells were pre-grown in phosphate-replete (Fig 15A and 15F) or phosphate-free (Fig 15B, 15C, 15D and 15E) medium prior to final growth in rich (YPD) medium for 4–5 hr (Fig 15A–15D) or 22 hr (Fig 15F); those pre-grown in phosphate-free medium exhibited stronger Gfp fluorescence in the inoculating cells, distinguishing them from daughter cells that had downregulated their *YWP1* expression in the YPD medium. Again, the greatest anti-HA signal was evident in the walls of the nascent daughter cells, with little in the walls of the mature cells, regardless of whether the Ywp1-HA-Ywp1 molecules also contained Gfp. (In this figure, the single micrographs that captured both the Gfp fluorescence and the antibody fluorescence show the Gfp fluorescence false-colored as blue.) Those cells with HA and Gfp in the same Ywp1 molecules (Fig 15A, 15B, 15C, 15E and 15F) demonstrated the *presence* of those molecules wherever there was Gfp fluorescence, but showed their *accessibility* to external antibodies only where there was red fluorescence. The ratio of red to green fluorescence was thus greatest where the HA was most accessible, and least where it was relatively inaccessible. Since the red fluorescence represented a greatly amplified HA signal relative to the intensity of the intrinsic green Gfp signal, that ratio was magnified in each case relative to the actual number of tagged Ywp1 molecules being detected. (Equal intensities would thus imply that relatively few of the molecules were accessible to antibodies.) As a fixation control, the cells in Fig 15C were processed exactly as for the other panels, except they were fixed with heat (60°C) rather than formaldehyde; the observed patterns appeared to be unaffected. (Unfixed and 40% ethanol-fixed cells also gave the same pattern, although there were some indications that storage in 40% ethanol might increase HA epitope exposure over time, an observation that merits further investigation.) In some preparations, anti-Gfp gave weak but positive labeling of some buds, but it was not apparent that the presence of the HA epitope might have affected the accessibility of the Gfp epitope in those cells. Similarly, weak signals and uncertainties about the accumulated number of tagged Ywp1 molecules precluded any conclusions about whether insertion of Gfp might have affected the accessibility of the HA epitope in any strain. Nevertheless, these results directly demonstrated the presence of tagged Ywp1 molecules and the reduction of their accessibility to external antibodies as the yeast cells grew and matured.

## Discussion

Ywp1 is an abundant glycoprotein of the yeast cell wall of *Candida albicans*, but is largely inaccessible to antibodies that might otherwise be useful for immune protection of the host and for probing of the spatial distribution of Ywp1 by the researcher. As shown here, insertion of Gfp between amino acids 165 and 166 had little effect on the known characteristics of Ywp1, and allowed its detection and localization throughout the wall, independent of external probes. Previous insertion of a probe into Ywp1 by others [28] took advantage of a multivalent HA epitope and well-characterized antibodies, and despite unanticipated changes in the structure of Ywp1 caused by this insertion, showed that it still accumulated in the yeast cell wall. Doubly-tagged versions of Ywp1 possessing both Gfp and HA have highlighted the dynamics of wall assembly by directly contrasting the presence of Ywp1 (through intrinsic Gfp fluorescence) with its accessibility to externally-applied anti-HA antibodies, and have demonstrated its diminished accessibility to those antibodies as the cells matured. Similar results were obtained with singly-tagged Ywp1-Gfp-Ywp1 using antibodies against Gfp and Ywp1, albeit with lesser signal strengths.

Rare cells in laboratory-grown cultures were observed to have anomalously high exposures of both native and tagged versions of Ywp1, and were isolated and further studied as stable strains. Not only were the Ywp1 and tag epitopes more accessible to antibodies in these strains, but the underlying β-1,3-glucan of the cell wall was also more exposed. Exposure of cell wall β-1,3-glucan is especially relevant to infections, as this pathogen-associated molecular pattern (PAMP) is recognized by the innate immune system of mammalian hosts, and is specifically engaged by the dectin-1 receptor of human macrophages, dendritic cells and neutrophils [5, 53, 54]. The molecular basis for this greater exposure of Ywp1 and glucan has not been determined for any of the strains described here, and is not necessarily the same for each of these strains, but appears to be due to changes in the cell wall rather than to changes in Ywp1 itself. The results described here thus provide new tools and shed new light on the structure, properties and dynamics of the cell wall of *C*. *albicans*, all of which are important for ultimately understanding how this opportunistic pathogen persists in its human host.

### Masking of cell wall glucans: Molecular mechanisms

The exposure of glucan that is normally masked or cloaked in the cell wall has been well studied in *C*. *albicans*, and has recently been computationally modelled [55], but is still poorly understood. Experimentally, exposure of wall glucans by *C*. *albicans* has typically been assessed and quantified by immune cell activation [14, 56–63], binding of soluble versions of dectin-1 [14, 56–58, 60, 64–69], or binding of mouse monoclonal antibodies, including an IgG_2b_ (2G8) [70] and an IgM (BFDiv) [59, 71, 72] that both recognize both β-1,3-glucan and β-1,6-glucan, and an IgG_1_ (#400–2) that is specific for just β-1,3-glucan [14, 57, 60–68, 73–77]. Screening of a *Saccharomyces cerevisiae* gene knockout library identified 79 mutants with greater binding of the anti-β-1,3-glucan IgG_1_ MAb, and all of those also bound more dectin-1 [14]; the deleted genes tended to be associated with polarized wall synthesis and remodeling, as well as protein mannosylation, consistent with potential masking sequelae. Deletion of some of the corresponding genes in *C*. *albicans* was similarly found to increase glucan exposure (18× for *kre5*, which is important for β-1,6-glucan synthesis, 11× for the β-glucan transglycosylase *phr2*, and 4× for the regulator *ssn8*) [14]. Other *C*. *albicans* genes found to increase glucan accessibility by a factor of 4–30 when deleted include the histidine kinase gene *chk1* [73], *gpi7*, involved in GPI anchor synthesis [61], the α-mannosyltransferase *mnn10* [63], and *cek1* and *hst7*, components of the MAPK signal transduction pathway involved in growth and cell wall biogenesis [64]; more modest increases were seen upon deletion of phosphatidylserine synthase *cho1* [57]. Gepinacin inhibits the Gwt1 GPI anchor biosynthetic enzyme and increased anti β-1,3-glucan binding 4–5× [74], while tunicamycin, an inhibitor of N-glycan addition, increased binding ~15× [76]. Low doses of Caspofungin reduce glucan accumulation [78], but also interfere with normal wall assembly [79], and resulted in greater accessibility of the glucan by a factor of 10–30 in one study [14] and 4–7 in another [67]; similar results were seen in the current study, with Ywp1 epitopes also becoming more accessible upon Caspofungin treatment. Glucan exposure was also observed to be increased by hypoxia [60], deletion of the *MNN2* gene family that increases outer fibril length [69], and upon growth in acidic (pH 4) media through signaling rather than increased quantities of glucan or hydrolytic loss of the potentially-masking acid-labile phosphmannans [62]. Perturbations to the normal synthesis and organization of the cell wall thus seem most likely to result in unmasking of the glucan layer, which would expose the cells to increased recognition and attack by innate immune cells. In the current study, deletion of Ywp1 was also found to result in increased β-1,3-glucan accessibility to the IgG_1_ MAb, consistent with the presence of Ywp1 in the yeast cell wall as an abundant mannoprotein that may itself contribute to molecular masking. Also, ectopic expression of Ywp1 in germ tubes and hyphae diminished the glucan accessibility of those structures. A multitude of mechanistic possibilities thus remain to be explored to explain the spontaneously-arising strains in the current study that were found to have normal amounts of Ywp1, but increased glucan accessibility, as well as increased accessibility of the epitopes of natural and tagged versions of Ywp1.

### Structure and processing of GPI proteins that may contribute to masking

For GPI proteins, which make up the bulk of the outer mannoprotein layer, little is known about spatial aspects of the anchorage of their C-terminal GPI remnants to the glucans of the cell wall [80], which presumably affects their ultimate accessibility. The layered arrangement typical of the *C*. *albicans* wall, with layers rich in chitin and β-1,3-glucan nearest the plasma membrane, overlain with a β-1,6-glucan-rich layer to which the GPI remnants are covalently linked, thereby anchoring the outermost mannoprotein layer [16, 17], raises many questions about spatial and temporal aspects of this assembly. Initial cleavage of GPI moieties from their proteins presumably occurs at the plasma membrane (into which the GPI acyl groups are embedded), leaving the freed ends of the proteins to be subsequently attached to β-1,6-glucan [80, 81]. Does this initial cleavage depend on a permissible conformation or interaction of the protein with the existing wall? Is the freed C-terminus able to explore or probe for potential glucan-linkage sites, such that the often N-glycosylated and bulky N-terminal end might only later find its way to the outer mannoprotein layer? Does the subsequent anchorage to glucan effectively lock the protein into position, or is there considerable rearrangement of the glucan and/or polypeptide chains afterwards? If the protein anchorage points are at the base of the outer mannoprotein layer, do the polypeptides necessarily extend outwardly from there, or can anchored polypeptides span the β-1,3-glucan-rich layer, with their N-terminal domains remaining in or migrating to the periplasm? How are these considerations and possibilities dependent on growth conditions? The positions of covalent anchorage points within the cell wall presumably influence the ability of the N-terminal ends of the proteins (where the adhesive and otherwise interacting domains tend to be localized [82, 83]) to be exposed or accessible on the outer surface of the wall. Correlations have been postulated and found between the overall length of certain GPI proteins and the surface accessibility of their N-terminal domains [84–87], but many other factors may also be important, including alternative forms of covalent linkage, such as disulfide bonds and alkali-labile bonds [84, 88]. Indeed, in one study, surface accessibility of *C*. *glabrata* Epa1 was neither linearly nor precipitously related to polypeptide length, and an N-terminal HA epitope was apparently present throughout the depth of the wall, irrespective of polypeptide length [85]. The Ywp1 polypeptide core is not as long as that of most adhesins, the N-terminal domains of which presumably require surface exposure for optimal functioning, suggesting that a less exposed orientation is compatible with or perhaps even required for the observed anti-adhesive effect of Ywp1. The relative position of the large N-glycan of Ywp1 may also be relevant, but is similarly unknown. Epitope (or tag) accessibility may thus be only partly related to the length of the polypeptide chain between the anchor and the epitope (or tag), necessitating empirical determination in all cases.

The poor accessibility of native and engineered epitopes of Ywp1 in normal yeast cells observed in the current study is not unprecedented for GPI proteins of *C*. *albicans*. When constitutively expressed, V5-tagged Iff8 and Rbt1 are accessible to anti-V5 antibodies in hyphae, but not in yeast; mild digestion with glucanase, however, exposes the epitopes in yeast [84, 89]. In contrast to Ywp1, which is detectable by Western blotting [21, 22], V5-tagged Pga13 is not detectable by Western blotting unless the protein is first chemically deglycosylated [90]. Epitope masking may thus result from a variety of factors. The epitopes of wild type Ywp1 are not well defined, since monoclonal antibodies have not yet been developed against this polypeptide, but some of the antisera used in the current study are spatially restricted to the N-terminal third of Ywp1; interestingly, those antibodies showed little binding to Ywp1 in the intact wall of the BWP17x strain, whereas antibodies elicited against the full polypeptide bound well. The orientation of Ywp1 in the cell wall and identity of components that normally shield Ywp1 thus remain unresolved, with possible contributions from the outermost, polysaccharide-rich, fibrillar layer as well as deeper components of the wall.

### Experimental distinction between absence and masking of cell wall epitopes

Apparent lateral inhomogeneities of epitopes in the walls of single cells can also be difficult to interpret, for the same reasons. For example, GPI protein Csa1 binds the most antibody at sites of cell surface elongation; subsequent reduction in that binding may be due to Csa1 downregulation, shedding, or epitope remodeling, but may also be due simply to masking as the wall matures [91, 92]. Immunolabeling of Als protein N-terminal domains with specific monoclonal antibodies is generally thought to quantitatively reflect the distribution of each of those proteins, consistent with the overall signal intensities that correlate with measured transcript levels for the population as a whole [93–95]; observed lateral differences in epitope signal intensity within the wall of a cell forming a germ tube, for example, may thus indicate temporal differences in synthesis or processing during wall biogenesis or remodeling, and differences between hyphae grown *in vitro* and *in vivo* may reflect expression differences. Considering the difficulty of accurately quantifying such molecules, however, there may also be a small or large component of epitope masking that generates the observed lateral nonuniformities in the cell wall signals, as appears to be the case for Ywp1. Potential differences between the presence of epitopes and their accessibility to probes may thus create significant uncertainty in protein localization and quantitation studies.

### Successes in detecting and tagging wall-anchored GPI proteins of *C*. *albicans*

More than one hundred different GPI proteins are predicted to be anchored in the cell wall of *C*. *albicans* [96]. As mentioned above, some of these polypeptides have epitopes that are accessible to antibodies applied to intact cells, revealing important spatial, temporal and conditional information about those proteins, subject to the listed caveats. Most notably, polyclonal antibodies specific for Hwp1 [44] and the Als family [97], as well as monoclonal antibodies specific for Csa1 [91, 92], Als1 [94, 98], Als3 [99, 100] and Als4 [93], have yielded valuable insights about their respective antigens. In addition, epitope tagging with short HA (YPYDVPDYA) and V5 (GKPIPNPLLGLDST) peptides has facilitated the detection and distinction of additional GPI proteins using widely-available anti-HA and anti-V5 antibodies [28, 84, 89, 101, 102]. Initial successes in the tagging of GPI proteins of *C*. *albicans* with intrinsically fluorescent polypeptides, detectable even if buried in the wall, involved replacement of the central regions of Hwp1, Als3 and Rbt5 with Gfp, leaving essential targeting signals at both ends, but losing the central majority of each polypeptide [103]. The same was done for Eap1 [86]. Subsequent constructs included all of the Hwp1 polypeptide in addition to the internal Gfp, and further confirmed the utility of such constructs for investigating wall-anchored GPI proteins, even though in these cases their expression was not driven by their native promoters [104]. Lossless insertion of Gfp into Pga59 and Pga62 shuttle plasmids, followed by targeted integration of those plasmids into the *C*. *albicans* genome, allowed expression of tagged versions of those two GPI proteins from their native loci and localization to the cell wall [105]. Despite the current availability of excellent tools for the direct insertion of fluorescent proteins into open reading frames of *C*. *albicans*, leaving no extraneous DNA in the genomes of the transformants [106, 107], and despite demonstration of efficacy for wall-anchored Hwp1 [106], such transformants remain rare, with the Ywp1-Gfp-Ywp1 of the present study perhaps the only example so far of a lossless, direct insertion of a fluorescent protein into a wall-anchored GPI protein of *C*. *albicans*. The localization and quantitation of cell wall proteins has tended to focus on those that are highly expressed, but the remaining multitude should also be amenable to current tagging technologies.

### Epitope accessibility is influenced by experimental manipulations and growth conditions

The accessibility of cell wall epitopes and ligands to relatively large probes such as antibodies and receptors can be modulated (usually increased) by various fixation and processing protocols, as has been observed for *C*. *albicans* wall glucans [14, 57, 58, 67, 69]. Such effects were found in the current study as well, but conditions that also preserved Gfp fluorescence tended to minimize those effects. Fixation can be avoided entirely, but unfixed cells may metabolically modulate their own walls as conditions change during experimental processing, especially with increasing time at physiologic temperatures, which will add uncertainty to any sort of quantitation. The morphology of *C*. *albicans* has also been found to affect glucan accessibility, with more [59, 67, 70, 71] or less [58] exposure of glucan in germ tubes and hyphae than in yeast forms, although preparation conditions and probe characteristics may account for the differences. In the current study, antibody binding to β-1,3-glucan was found to be much greater in germ tubes than in yeast forms in a routine set of experimental conditions, and ectopic production of Ywp1 by germ tubes and hyphae was found to diminish glucan exposure. Greater glucan exposure has been seen *in vivo* (in infected tissue) than *in vitro* (in cultured cells) [60, 77], highlighting the operational nature of most assessments. In budding yeast forms of *C*. *albicans*, glucan has been observed to be more exposed at bud necks and bud/birth scars [14, 58, 59, 64, 70], and similar results were seen here for glucan as well as for Ywp1. A less-appreciated factor has been the growth phase of the cells, highlighted in the current study, with much greater exposure of β-1,3-glucan, native Ywp1, and tagged Ywp1 in rapidly growing walls of nascent daughter cells. Even though Ywp1 contributes to glucan masking in normal cells, the increase in glucan masking as yeast cells mature relies on additional mechanisms, as it occurs even in the absence of Ywp1. Studies that attempt to quantify glucan exposure must thus take into consideration numerous aspects of population growth and measurement.

Phosphate limitation during growth of yeast forms greatly diminishes the quantity of phosphodiester-linked β-1,2-mannotriose on the surface (S4 Fig), but has a minor effect on the accessibility of cell wall Ywp1 and β-1,3-glucan to antibodies (Figs 2G, 2H and 4B). This is consistent with the observation that genetic ablation of mannosylphosphate does not increase recognition of *C*. *albicans* by macrophages [48], which might be expected if more glucan were exposed on those cells. Phosphate limitation during growth of yeast forms also upregulates *YWP1* expression [22]. Interestingly, relative to phosphate replete cultures, phosphate starved cultures have been found to accumulate at least 10× more Ywp1-Gfp-Ywp1 in their cell walls, while the increase in accumulated cytosolic Gfp reporting expression of *YWP1* is less than 10× (*e*.*g*., Fig 9, S5 Fig, and [22]). (The increase in secreted Ywp1-Gfp has been harder to quantify because it does not accumulate in the cells, and the amount produced depends on the stage of growth at which the culture senses phosphate limitation.) The basis for this difference between cytosolic and wall-anchored Gfp is unknown, but may include differences in biosynthetic processing or turnover that are affected by phosphate availability, or technical limitations or artifacts of the measurements. It is illustrative of the difficulty of quantifying cellular proteins under different growth conditions.

### Potential artifacts of genetic probe insertion

In the current study, Gfp was inserted 32 amino acids downstream from the N-terminus of the Ywp1 core that is created by posttranslational propeptide cleavage. Some portion or all of this 32 aa segment is responsible for the strong, persistent, noncovalent association of the cleaved propeptide with the downstream core of Ywp1 [21]. The HA epitope assembly was inserted within this 32 aa segment, just 9 aa from the N-terminus [28], and as such apparently inhibits cleavage of the propeptide. It also inhibits attachment of the sole N-glycan to the asparagine that is just 6 aa upstream from the tribasic cleavage site. The anomalous electrophoretic properties caused by the HA insertion may be a result of these secondary effects rather than to the HA assembly itself. Whether this apparent aggregation or conformational change in the polypeptides occurs before or after secretion, or during subsequent experimental manipulation, is not known. Persistent, low mobility forms have also been observed during SDS-PAGE for other cell wall proteins, including purified flocculin Flo11 of *Saccharomyces cerevisiae* [108] and purified, anchor-negative versions of *C*. *albicans* Als5 that have a V5 epitope and 6×His tag on their C-termini [109]; the latter aggregates are thought to be due to amyloid formation through short segments of Als5 that have high (93%) β-aggregation potential [110, 111]. Secreted Ywp1-HA-Gfp has two segments with similarly high (94–98%) β-aggregation potential (LAAFFTFV and VQFYYTIV positioned in the Ywp1 propeptide), and it is conceivable that these segments might manifest themselves and mediate aggregation in the abnormal absence of N-glycosylation and propeptide cleavage that has been observed for the Ywp1-HA-Gfp constructs.

### Advantages and limitations of Gfp as a cell wall tag

Along with the many advantages of Gfp as a tag for fungal cell wall proteins come several limitations that were evident in the current study. Inserted into Ywp1, Gfp evidently interfered to some extent with the normal biosynthesis or transport of the glycoprotein to the cell wall, as it accumulated in the wall in lower quantities than expected (as compared to cytosolic Gfp made through the same *YWP1* promoter, as mentioned above). The problematic step or steps have not been determined, but post-secretion wall anchorage did not appear to be one of them, as little if any of the Gfp fluorescence was lost to the culture medium. Furthermore, the fluorescence of Gfp is pH dependent [112], so that optimal signal strengths necessitate viewing and measurement at a pH of around 8, which is typically much more alkaline than conditions that foster the growth of yeast forms. Direct measurements in even slightly acidic media may thus miss or underestimate the quantity of Gfp present. The stability of Gfp is also pH dependent, with temporary or permanent loss of fluorescence occurring at pH values substantially below 6; cultures must thus be carefully buffered to prevent them from ever moving too far toward the pH of 2 attained in unbuffered MM13, for example, if Gfp fluorescence is to be used quantitatively. Similar instability was recently observed for Gfp as well as a version of Gfp with pH-dependent fluorescence properties in wall-anchored Pga59 of *C*. *albicans* [113]. Some morphological transitions of *C*. *albicans* are strongly influenced by temperature, but nascent Gfp folds and becomes fluorescent less efficiently as the temperature increases within that range [80], potentially limiting the use of Gfp as a quantitative marker in some experimental situations. Finally, fluorescence excitation and emission filters commonly found in microscopes and flow cytometers do not discriminate between Gfp fluorescence and the fluorescence of other cellular components, notably flavins, which can result in diminished signal-to-noise ratios for Gfp at lower levels of expression [114]. Alternative fluorescent proteins [115] may thus be better tags for some experimental situations. Despite these limitations, however, the intrinsic fluorescence of Gfp obviates potential accessibility issues of epitope and ligand tags that may cause them to be masked and therefore undetectable. Also, the otherwise robust nature of Gfp fluorescence was an advantage in the current study, as it largely survived heating to 60°C (and even 50°C in the presence of excess SDS), 8M urea, mild fixation with formaldehyde, and ethanol up to about 70%, properties evidently unaffected by the Ywp1 sequences appended to each end of Gfp or to the deletion of the five C-terminal amino acids of Gfp.

### Reassessment of Ywp1 as a strictly yeast-specific marker

*C*. *albicans* can assume a variety of unicellular yeast-form and multicellular filamentous morphologies, depending on environmental conditions and host niche [18]. Although filamentous, the pseudohyphal form in some ways more resembles yeast than true hyphae, in that nuclear division occurs across the mother-daughter neck, constrictions are present at those junctions, and the cells are elongated ovoids rather than parallel-sided [19]. Regarding pseudohyphae as an intermediate or transitional state between yeast and hyphae is supported by the difficulty of creating homogeneous pseudohyphal cultures and by the genetic demonstration of a dose-dependent continuum of morphologies from yeast to hyphae that follows the expression level of the transcription factor *UME6* [116]. The expression of *YWP1* is strongly upregulated under growth conditions that favor yeast forms, and strongly downregulated under hypha-favoring conditions (reviewed in [21]), but the status of *YWP1* in pseudohyphae is not well characterized. Using soluble, cytosolic Gfp as a reporter of *YWP1* expression previously left open the possibility that Gfp or its mRNA might passively diffuse from mother yeast cells to daughter filaments and incorrectly suggest expression by the cells of those filaments [21]. The wall-anchored Ywp1-Gfp-Ywp1 reported here allows the first direct look at the status of anchored Ywp1 in individual pseudohyphae, and has revealed that Ywp1 is not exclusively restricted to the walls of yeast forms, although not always present in pseudohyphae. In cultures designed to produce pure populations of yeast or hyphae, pseudohyphal forms were occasionally seen; they were less rare under intermediate culture conditions, but still morphologically diverse enough to preclude regarding any of them as typical. Often, but not always, these forms had Gfp-tagged Ywp1 in their walls, but usually less abundantly than in the walls of the surrounding or attached yeast forms. The presence of some negative daughter pseudohyphae (and all germ tubes) emanating from positive yeast cells confirmed that wall-anchored Ywp1 did not significantly diffuse laterally into the daughter walls. Thus, if pseudohyphae are indeed intermediates between yeast and hyphal states, there appear to be conditions in which *YWP1* continues to be expressed in pseudohyphal morphotypes. The Gfp-tagged strains created here may help to better define these intermediate stages and explore their stability and properties through more systematic studies.

### Future research

The spontaneous changes that resulted in greater epitope accessibility in the cell walls of three different strains of *C*. *albicans* that were isolated and described here have not been identified, nor is it known if there is any overlap among those three. Whole genome sequencing might pinpoint those changes, or at least provide candidates for further investigation, and reveal whether they are new or previously described genes that contribute to masking. By normally providing barriers to recognition by the immune system, they may be useful therapeutic targets. Isolation of such variants from virulent strains (rather than from attenuated laboratory strains as reported here) will allow direct assessment of their ability to resist host immune attack.

The study of Ywp1 will greatly benefit from development of specific monoclonal antibodies. In current and past studies, the anti-Ywp1 mouse sera generated by DNA vaccination have been quite limited in quantity and titer. Even though these sera have shown little or no off-target binding to yeast forms of *C*. *albicans*, alternative reactivities in these sera have often shown binding to unidentified antigens of filamentous forms (as assessed by immunofluorescence microscopy). The existing sera were therefore not useful for looking at the presence or accessibility of Ywp1 in pseudohyphae, for example, or of ectopic Ywp1 in hyphae. Ywp1 has multiple antigenic polypeptide epitopes, and monoclonal antibodies specific for these will facilitate studies of Ywp1 localization, quantitation and accessibility. Furthermore, glycosylation mutants of *C*. *albicans* [117] may then be useful for revealing whether any of the glycans of Ywp1 mask any of the polypeptide epitopes of Ywp1.

Ywp1 evidently has an antiadhesive effect as well a masking effect when anchored in the cell wall. The molecular mechanism remains unknown. The possibility that one or both of these effects is due to the sole N-glycan rather than the polypeptide backbone of Ywp1 has not yet been explored. This question may be readily answerable by creating a Ywp1 mutant that lacks this N-glycan (for example, a homozygous version of Ywp1 with N115Q or T117V mutations). In addition, since this single N-glycan is attached to the 11 kDa cleaved propeptide that remains firmly associated with the Ywp1 core but is dissociable with hot SDS, a monoclonal antibody specific for the propeptide could be used to immunoaffinity purify this glycan; its structure could then be determined and compared to other N-glycans from the same cells, and the relationship of its structure to growth conditions could be assessed. Similarly, ectopic expression of Ywp1 by hyphae [21] would allow yeast-hyphal differences in glycosylation to be further delineated, and in a more refined way than looking at wall glycans in bulk [118, 119]. Ywp1 would then join a secreted, engineered version of a single N-glycan carrier that has already been developed and investigated in *C*. *albicans* yeast forms [120], but Ywp1 would additionally represent a wild type carrier with wall anchorage.

Even though nutrient limitation in general does not appear to upregulate *YWP1* expression, sulfur limitation has recently been found to result in substantial induction of *YWP1*, although not to the same extent as phosphate limitation. Although both phosphate and sulfate can bind to the same protein domains, it seems likely that phosphate homeostasis is controlled by intermediary inositol polyphosphate signaling molecules [121, 122], and that sulfur sensing and response involve independent pathways [123]. The effect of sulfur starvation on epitope accessibility has not yet been investigated for *C*. *albicans*, but the effect on *YWP1* expression is documented here to inform future investigation (see S11 Fig).

Finally, wall-anchored Ywp1-Gfp-Ywp1, which is present in yeast forms but absent from hyphae, may be a useful tool for identifying and studying the various occurrences and forms of pseudohyphae. The triple auxotrophy (*Δarg4 Δhis1 Δura3*) of some of these strains produced here will facilitate additional genetic manipulations.

## Supporting information

S1 File Description of pGEM-GFP-URA3-GFP. (PDF)

S1 Fig Some growth conditions result in cell morphology differences. Flow cytometry further revealed that BWP17x under some growth conditions apparently exhibits delayed mother/daughter cell separation relative to BWP17c, resulting in differences in light scattering profiles (S1A Fig). This panel represents the same experiment as shown in Fig 2A, but includes an additional antiserum as well as a control with just secondary antibody. A separate experiment that utilized an alternative secondary antibody and fluorochrome revealed the same patterns (S1B–S1D Fig). Mean and median forward scatter (FSC) values were 1.2–1.3× greater for BWP17x than BWP17c, and side scatter (SSC) values were 1.6–1.7× greater; fluorescence values were 57–64× greater, however, indicating that the per-cell antibody binding differences could not be attributed to differences in cell size or granularity. In other experiments, however, there was often little difference between the light scattering profiles of BWP17c and BWP17x, depending on the medium, phosphate availability, and whether growth was in liquid culture or on solid agar (S1E–S1I Fig). This phenomenon has not yet been investigated systematically, and critical parameters have not yet been discerned for these differences.
(TIF)

S2 Fig Comparison of Ywp1 protein from BWP17c and BWP17x. SDS-PAGE followed by protein staining with Coomassie Blue was used to visualize the cleaved, deglycosylated propeptide (*) of Ywp1. Two independent colonies (1 and 2) of BWP17c (“c”) and BWP17x (“x”) were each grown to stationary phase in phosphate-limited BMM13. Culture supernatants, 50°C SDS extracts, and subsequent 70°C SDS extracts were precipitated with ethanol, deglycosylated with PNGase F, and resolved by SDS-PAGE. Each lane represents 2.5 ml of culture. The image includes the stacking gel at the top and marker proteins (M) on the right (with masses shown in kilodaltons). The propeptide quantities and extraction properties appear similar for BWP17c and BWP17x, but more total protein (including the inducible acid phosphatase Pho100 migrating at ~29 kDa) was extracted by SDS from the latter strain, suggesting differences in wall structure or permeability.
(TIF)

S3 Fig BWP17c and BWP17x differ in their growth sensitivities and adhesion properties. (A) Stationary phase yeast cultures were serially diluted 1/8 (5 times, left to right) and spotted onto YPD agar containing the indicated compounds. The arrays were photographed after 44 hr of growth at 30°C. Sensitivity reduces colony size and/or number. (B) Droplets of dilute yeast microcultures were arrayed on a polystyrene plate and grown to stationary phase in MM13 at 30°C; nonadherent cells were gently rinsed away, and the adherent cells were stained with Crystal Violet [21, 22]. Two independent colonies of BWP17c and two independent colonies of BWP17x were compared; as controls, strain SC5314 (wild type parent of BWP17) and strain 4L1 (Ywp1-negative *ywp1*::*ARG4 / ywp1*::*URA3*) were included in duplicate.
(TIF)

S4 Fig Inverse relationship of Ywp1-Gfp-Ywp1 and mannotriose accumulations in phosphate-limited strain YGY. Yeast cells were grown to stationary phase in batch liquid cultures of BMM13 that started with 2.0, 0.5 or 0.2 mM phosphate, as indicated; this resulted in no phosphate starvation (2.0 mM) or phosphate starvation being experienced by a progressively greater proportion of each population. The cells were fixed with formaldehyde, labeled with anti-mannotriose mAb G11.1 followed by a fluorescent red secondary antibody, and analyzed by flow cytometry. Earlier limitation of available phosphate correlated with increased Ywp1-Gfp-Ywp1 accumulation and decreased phosphodiester-linked mannotriose accumulation.
(TIF)

S5 Fig Comparison of two *YWP1-GFP* strains by flow cytometry. In one strain (BJ4eS8, equivalent to independent transformant BJ3 that was described previously [22]), *GFP* is a reporter of *YWP1* expression and generates soluble Gfp that accumulates in the cytosol; in the other (strain YGY), *GFP* is inserted into the coding sequence of *YWP1*, generating a Ywp1-Gfp-Ywp1 glycoprotein that becomes anchored in the cell wall. Each strain has one copy of *GFP* inserted into one of its two alleles of *YWP1*. Parallel cultures of yeast forms were grown in BMM13 containing surplus (2.5 mM) or limiting (0 mM) phosphate (P_i_). Aliquots of live cells were periodically analyzed for Gfp fluorescence, forward scatter (a function of size), and side scatter (a function of complexity), as shown; the latter two parameters confirm that the increases in fluorescence over time were not attributable to cell aggregation or diminished cell separation. Semi-synchronous growth and budding slightly complicated fluorescence quantitation at early time points in this analysis using ungated samples, as unseparated mother-daughter pairs were measured as single events (as evidenced by the forward scatter and side scatter plots), but in the phosphate-limited cultures the fluorescence rose steadily. A lag period of ~2 hr was followed by yeast growth and budding; first separation of daughter cells from mother cells was prevalent between 4 and 8 hr, but showed less synchrony thereafter. Each point represents the mean or median of 100,000 events; single yeast cells and unseparated mother-daughter doublets are each measured as one event. Phosphate starvation increases *YWP1* expression; in the first 24 hr of these cultures, phosphate starvation increased the mean accumulated cytosolic Gfp 7× and the wall-anchored Gfp 40× (relative to phosphate-replete cultures).
(TIF)

S6 Fig Comparison of propeptide properties of Ywp1 and Ywp1-Gfp-Ywp1. Two derivatives of strain YGY were compared: One with only wild type Ywp1, and the other with only Ywp1-Gfp-Ywp1 (as in Fig 8C and 8D), as indicated below each lane. Samples were prepared as described for S2 Fig. Each lane represents 2.5 ml of stationary phase BMM13 culture that started with 0.2 mM phosphate. The deglycosylated Ywp1 propeptide band is indicated with an arrow. Lanes are from two identical gels run simultaneously in the same apparatus.
(TIF)

S7 Fig Effect of Gfp insertion on the antiadhesive effect of Ywp1. Nine strains were cultured as individual droplets in a polystyrene plate in unbuffered BMM13 containing either 2.5 mM phosphate (left panel) or 0.1 mM phosphate (middle panel). The number of wild type *YWP1* alleles per strain is 2 (G), 1 (A, B, E, F) or 0 (C, D, H, I); A secretes Ywp1-Gfp, while B, D, F and H have wall-anchored Ywp1-Gfp-Ywp1. This is shown schematically in the right panel as yeast cells possessing no Ywp1 or wall-anchored Ywp1 (Y-), secreted Ywp1-Gfp (YG), and/or wall-anchored Ywp1-Gfp-Ywp1 (YGY-). Wall-anchored Ywp1-Gfp-Ywp1 thus confers an antiadhesive effect; this effect may be comparable to wild type Ywp1, considering that Ywp1-Gfp-Ywp1 is less abundant in the wall.
Detailed description of strains (all derived from BWP17):
A    Strain YG        BWP17 with *GFP-URA3* inserted into one allele of *YWP1* to encode secreted Ywp1-Gfp
B    Strain YGY        Strain YG (A) that has lost *URA3* to encode wall-anchored Ywp1-Gfp-Ywp1
C    4L1        Ywp1-negative double knockout (*ywp1*::*ARG4 / ywp1*::*URA3*)
D    Strain YGY-Y        Strain YGY (B) in which the remaining wild type *YWP1* allele was disrupted with *URA3*
E    Strain YGY-G        Strain YGY (B) in which the *YWP1-GFP-YWP1* allele was disrupted with *URA3*
F    Strain YGY+U        Strain YGY (B) in which *URA3* allele was inserted outside of either *YWP1* locus
G    DAY185        BWP17 with its auxotrophies restored by insertion of *ARG4*, *URA3* and *HIS1*
H    Strain YGY-Y’        Subclone of Strain YGY-Y (D) with similar intensity of Gfp fluorescence
I    Strain YGY-Y-G        Subclone of Strain YGY-Y (D) with no Gfp fluorescence (spontaneous mutant).
(TIF)

S8 Fig Comparison of the properties of strains YHY and YHYx. (A) Stationary phase yeast cultures were serially diluted 1/10 (5 times, left to right) and spotted onto YPD agar containing the indicated compounds. The arrays were photographed after 69 hr of growth at 30°C. Sensitivity reduces colony size and/or number. (B) Droplets of dilute yeast microcultures were arrayed on a polystyrene plate and grown to stationary phase in MM13 at 30°C; nonadherent cells were gently rinsed away, and the adherent cells were stained with Crystal Violet. Each spot was grown from one colony picked from a streaked plate during the isolation procedure; genetic analysis identified them as either YHY or YHYx, as indicated, with the latter phenotypically more adhesive, as revealed here. (C) SDS-PAGE followed by protein staining with Coomassie Blue was used to visualize the cleaved, deglycosylated propeptide (*) of Ywp1. Three subpopulations (c; strains YHY) containing less than 0.1%, 0.1% and 10% (respectively, left to right in each set) of high-binding cells were compared to a clonal population (x; strains YHYx) with 100% high-binding cells; each was grown to stationary phase in phosphate-limited BMM13. Culture supernatants, rinses with Tris/EDTA/NaCl at pH 8.1, 50°C SDS extracts, and subsequent 70°C SDS extracts were precipitated with ethanol, deglycosylated with PNGase F, and resolved by SDS-PAGE. The image includes the stacking gel above the resolving gel (as in S2 Fig).
(TIF)

S9 Fig Sensitivity of Ywp1-HA-Gfp to secreted acid protease activity. Cells were grown for 45–46 hr in shaking 30°C batch cultures in BMM13 (containing 100 mM MES, 80 mM Bis-Tris and 0.02% Tween-80) that started with 0.3 mM phosphate. Culture supernatants (pH ~5.9) were alkalinized to pH ~8.0 with 240 mM Tris and 9 mM EDTA. For the lower panel, half of each alkalinized supernatant was then heated to 60°C for 30 min; the other half was kept at 24°C, as indicated. All supernatants were then subjected to centrifugal ultrafiltration through a 10K MWCO filter, and the retained proteins were washed with 10 mM Tris / 1 mM CDTA (pH 7.6). Top panel: Ultrafilter retentate containing Ywp1-3HA-Gfp (derived from a strain YHYx parent). Two aliquots were heated to 70°C for 5 min (Δ), and six aliquots were acidified to pH 5.15–6.31 as indicated by adding MES to 167 mM and Bis-Tris to 17–150 mM. All aliquots were incubated 24 hr at 24°C, then mixed with electrophoresis buffer (giving final concentrations of 1% SDS, 20 mM DTT, 200 mM Tris, 100 mM HCl, 2% Ficol-400, 40μM Phenol Red, pH 8.3) and divided in half, with one of each pair heated to 95°C for 3 min (as indicated below the upper panels) before subjecting both to SDS-PAGE. Laser scanning for Gfp fluorescence (right panel) was followed by staining for protein with Coomassie Blue R-250 (left panel). Note that both heating to 70°C and increasing the pH to above 6 each inhibited the activity of endogenous protease(s), diminishing the conversion of the low-mobility smears to sharp mid-gel bands, which consist of fluorescent Gfp but no detectable HA epitopes (as determined by Western blotting). The slight degradation evident in the inhibited samples may have occurred during growth of the culture at pH 5.9. Lower panel: Retentates contained Ywp1-6HA-Gfp or Ywp1-3HA-Gfp (derived from strain YHY or YHYx parents, as indicated). Aliquots of heated (60°C) and unheated (24°C) washed retentates were left at pH 7.6 or acidified to pH 5.8 with 125 mM MES and 50 mM Bis-Tris, then incubated for 19 hr at 24°C before adding SDS sample buffer (as above) and heating to 50°C for 10 min (which did not affect the Gfp fluorescence but would have dissociated any cleaved Ywp1 propeptide from the Ywp1 core). As indicated under the lower panel, one culture was grown in the presence of the protease inhibitor N-acetyl-leucyl-leucyl-norleucinal (ALLN): 20μM was added at the beginning of the culture and after 22 hr of growth. An extra 40 mM ammonium chloride (in addition to the 80 mM already present) was also added to this culture to ensure that the nitrogen source was not limiting, but other experiments suggested this was unnecessary. In conclusion, both heating to 60°C and alkaline pH each inhibited the activity of endogenous protease(s), as did the ALLN. **Note** that the major fluorescent, acid-dependent degradation product is a single band for Ywp1-6HA-Gfp and a double band for Ywp1-3HA-Gfp; the reason for this difference is unknown, but may be related to the proline-to-serine mutation in Ywp1-3HA-Gfp described in the text. Intriguingly, in comparison to the Ywp1-3HA-Gfp bands in the upper panels, the single Ywp1-6HA-Gfp band comigrates with the lower band in the unheated samples and with the upper band in the heated samples. **Note** also (in the lower panel) that less Ywp1-6HA-Gfp accumulated in the culture medium of a strain derived from strain YHY than from strain YHYx (~60% as much, according to fluorometric measurements), and there was also less acid protease activity; how this might be related to the greater accessibility of the HA epitope in the YHYx strains is not known.
(TIF)

S10 Fig Mannosidase digestion of Ywp1-6HA-Gfp. Secreted Ywp1-6HA-Gfp (derived from parental strain YHYx) was concentrated by ultrafiltration (as described for the upper panels in S9 Fig) or by precipitation with an equal volume of ethanol, as indicated. Aliquots of the ultrafiltration retentate were kept at 24°C or heated to inactivate acid proteases (5 min at 70°C, or 3 min at 95°C, as indicated); ethanol precipitation eliminated most of the acid protease activity. Each aliquot was divided in half and incubated for 24 hr at 24°C with (+) or without (-) α(1–2,3,6)-mannosidase in a zinc-containing sodium acetate buffer at pH 5.0 (slightly above the pH optimum of the enzyme, but not low enough to denature the Gfp). Electrophoresis buffer containing SDS and DTT (as described for S9 Fig) was added, and half of each sample was heated to 95°C for 3 min (as indicated below the panels) before SDS-PAGE, fluorescence scanning, and protein staining (as described for S9 Fig). Marker masses (in kilodaltons) are shown on the left. The mannosidase (30 mU per lane) appears as a sharp band near the top of the resolving gel; it converts to smaller (~46 and ~60 kDa) bands upon heating to 95°C in SDS. Control experiments using the chromogenic substrate p-nitrophenyl-α-D-mannopyranoside showed that the mannosidase was highly active in the pH 5 buffer and was not inhibited by anything in the culture supernatant retentate; the retentate also had no mannosidase activity of its own. In conclusion, the mannosidase caused no obvious mobility shift in Ywp1-6HA-Gfp, suggesting that if any O-glycans are present, they are too short or scarce to be responsible for the anomalous migration of undegraded Ywp1-6HA-Gfp. Similar results were obtained for Ywp1-3HA-Gfp.
(TIF)

S11 Fig Sulfur starvation also increases *YWP1* expression, but to a lesser extent than phosphate starvation. Flow cytometric analyses of *YWP1* expression reported by wall-anchored Ywp1-Gfp-Ywp1 (strain YGYx) and cytosolic Gfp (strain BJ4eS2) show induction upon sulfur limitation during growth. Cells were grown in 30°C MM13 with starting concentrations of sulfate (S) and phosphate (P) as indicated (1 mM MgSO_4_ in MM13 was replaced with 1 mM MgCl_2_, then ammonium sulfate was added to 25–600 μM; for B and C, 5 mM KH_2_PO_4_ was replaced with 5 mM KCl, then KH_2_PO_4_ was added to 0.1–3.0 mM). Cultures were buffered with 50 mM phthalic acid and 130 mM (A) or 150 mM (B,C) Bis-Tris, and shaken in flasks for 65 hr (A) or 46–48 hr (B,C) prior to alkalinization to pH 8 for flow cytometry. Mean fluorescence values estimate the *YWP1* expression: Panel A suggests that sulfate becomes limiting in MM13 at initial concentrations of less than about 250 μM. In B, under phosphate-replete conditions (3 mM), sulfate limitation (50 μM *vs* 300 μM) resulted in 2–3× more fluorescence, while under sulfate-replete conditions (300 μM), phosphate limitation (0.3 mM *vs* 3.0 mM) resulted in about 20× more fluorescence. In A, sulfate limitation (50 μM *vs* 300 μM) resulted in about 7× more fluorescence, but this ratio may be artificially high because the increased growth of the 300 μM culture dropped the pH to about 0.3 units lower than the 50 μM culture, which may have adversely affected the stability of the external Gfp (especially since these cells were at this pH in stationary phase for 18–20 hr longer than the cells in B, which also started 0.2 pH units higher because of an extra 20 mM Bis-Tris in their medium). Indeed, Gfp that was exposed only to cytosolic pH shows only a doubling of the fluorescence upon sulfate limitation (C). The *YWP1* induction upon phosphate limitation thus appears to be about an order of magnitude greater than its induction upon sulfur limitation, as assayed by wall-anchored Gfp; for unknown reasons, cytosolic Gfp (cf. S5 Fig) reports a smaller difference. **Note:** Elimination of the sulfate from BMM13 was found to have little effect on cell growth, but elimination of both the sulfate and the MES buffer stopped growth. Even 1 mM MES supported growth in the absence of sulfate, suggesting that the cells obtained sulfur from MES itself rather than from a contaminant. The same was also observed for HEPES and DMSO as sulfur sources. Phthalic acid was thus used in place of MES for pH buffering (in combination with Bis-Tris).
(TIF)
