# Supplementary material for: Accessibility and contribution to glucan masking of natural and genetically tagged versions of yeast wall protein 1 of Candida albicans
Source: PLoS One. 2018 Jan 12;13(1):e0191194. doi: 10.1371/journal.pone.0191194 (PMC5766240; doi:10.1371/journal.pone.0191194)
Supplement: S1 File — (PDF) [file pone.0191194.s001.pdf]

S1 File

Description of pGEM-GFP-URA3-GFP and instructions for use

This plasmid serves as a template for the creation of yeast transfection cassettes that can result in the insertion of green fluorescent protein (Gfp) into any endogenous protein of *Candida albicans*. As detailed below, the initial transformation results in a chimeric fusion protein that has full-length Gfp at its C-terminus and any portion of the endogenous protein at its N-terminus; subsequent growth in the presence of 5-fluoroorotic acid (5-FOA) selects for loss of an internal *URA3* gene and consequent fusing of the Gfp in-frame to the C-terminal portion of the endogenous protein. Thus, Gfp thus becomes embedded within the endogenous protein; it can be inserted between consecutive amino acids of the endogenous protein, or it can replace an internal segment, as desired. Flanking genomic regions remain unaltered, fostering native regulation and expression of the Gfp-tagged version of the endogenous protein.

Features of pGEM-GFP-URA3-GFP

Arrangement of components (not to scale):

pGEM vector	Full *GFP* coding sequence	*URA3* gene (coding sequence + flanking regions)	Partial *GFP* coding sequence	Optional linker	pGEM vector

Numbering starts at the first nucleotide of the unique *Nsi* I site:

1-62 Multiple cloning site (*Nsi* I to *Spe* I) of pGEM-T [which is Promega pGEM-5Zf(+) that has been cut with *Eco*R V and then T-tailed]

63 Synthetic, non-templated nucleotide added by *Taq* polymerase during PCR

80-796 Full coding sequence of yEGFP3 (yeast enhanced green fluorescent protein 3) of Cormack *et al.* [1]; similar to GenBank accession number U73901; codon-optimized for *Saccharomyces cerevisiae* and *Candida albicans*; has the fluorescence-enhancing S65G and S72A mutations.

797-2179 *URA3* from *Candida albicans* strain 3153A, consisting of the 819 bp coding sequence, 435 upstream bp (including the promoter for *URA3* as well as the presumptive transcription terminator of the upstream *IRO1* gene, which serves as the terminator for *GFP*), and 129 downstream bp (through the *Xba* I site).

2176-2374 Partial coding sequence of yEGFP3. These 199 bp are an exact, direct repeat of nucleotides 580-778. This segment facilitates looping out and loss of the intervening *URA3* segment by homologous recombination.

2375-2398 Encodes a linker segment (SSASPSGS) that matches a segment of *Candida albicans* yeast wall protein 1 (Ywp1). In the final transfection construct, this segment can be partially or fully included, excluded, or replaced with an alternative sequence, if desired.

2399-5300 pGEM-T [Promega pGEM-5Zf(+)], from the unique *Apa* I site up to (but not including) the unique *Nsi* I site.

Additional notes

pGEM-GFP-URA3-GFP is a derivative of pGT-GFP-URA3-14 of Granger *et al.* [2], which has GenBank accession number AY656808; nucleotides 1-2179 exactly match nucleotides 1-2180 of AY656808 except for the deletion of one erroneous nucleotide between 2132 and 2133. pGEM-GFP-URA3-GFP is available from Addgene (www.addgene.org/72606/).

The 199 bp partial sequence of *GFP* (nucleotides 2176-2374, as numbered above) omits the last 5 codons and stop codon of *GFP*, so that the final Gfp that is inserted internally into the endogenous protein lacks the C-terminal 5 amino acids of Gfp (*i.e.*, -DELYK.) This segment is unnecessary for creation of a stable, fluorescent Gfp [3].

Instructions for Use

A PCR amplicon encompassing the *GFP-URA3-GFP* segment serves as the transfecting DNA for *ura3* strains of yeast (which require uracil or uridine for growth because their *URA3* is defective or missing). Transformants grow in the absence of exogenous uracil or uridine upon stable integration of the *GFP-URA3-GFP* cassette into their genome by homologous recombination. PCR primers are designed to target the insertion to the point of interest (within the coding sequence of a gene). Subsequent growth of transformants in the presence of 5-FOA selects for rare individual cells that have lost their inserted *URA3* through recombination of the homologous flanking *GFP* sequences (which share 199 bp of identical sequence); this fuses the upstream *GFP* coding sequence with the downstream coding sequence, resulting in a protein with an internal Gfp insertion.

PCR primer design: 80-nucleotide DNA primers have been used routinely and successfully for these transformations. The 5’ 60 or so nucleotides should match the genomic insertion target, and the 3’ 20 or so nucleotides should match the ends of the *GFP-URA3-GFP* cassette; the amplicon will therefore have about 60 base pairs of target sequence at each end, with any length of sequence between those two targets in the genomic DNA (even none, if a lossless insertion is desired rather than a replacement). The upstream primer sequence is an in-frame fusion of the target coding sequence and the *GFP* coding sequence (possibly bypassing the first codon or two of *GFP*, if desired). The downstream primer sequence is an in-frame fusion of the complement of the target sequence and the complement of the end of the *GFP* gene (with or without part or all of the optional linker segment, which encodes SSASPSGS).

Success has routinely been achieved using primers from IDT that have simply been desalted, not gel-purified.

If your PCR polymerase is likely to add an untemplated “A” to the 3’ ends of the amplicons, make sure this addition will match the target sequence (*i.e.*, the target should have a “T” immediately upstream from the first [5’] base of your primers).

For the most efficient amplification of the *GFP-URA3-GFP* cassette from the pGFP-URA3-GFP plasmid, pre-digest the plasmid with *Hin*d III and *Apa* I. The liberated 2.33 kbp insert can also be gel purified for amplification if you anticipate multiple uses of this cassette.

This system has been used successfully in each attempt with six different strains of *Candida albicans* (all derivatives of strains CaI4 and BWP17, which are both derivatives of strain SC5314); it has not yet been attempted in other strains, species or genera. So far, it has been tried only for a GPI-anchored cell wall protein (Ywp1); the initial transformants secreted the Ywp1-Gfp fusion protein into the culture medium, as the chimera lacked the C-terminal anchor of Ywp1; the 5-FOA survivors incorporated Ywp1-Gfp-Ywp1 into the cell wall, as no amino acids were altered or lost from Ywp1 in this assembly and the inserted Gfp did not prevent transport to that destination.

Alternatives

A similar plasmid template (pMG2082 = pGUG = pGFP-URA3-GFP = pGF-URA3-FP) has been described by Gerami-Nejad *et al.* [4]. That plasmid consists of *URA3* flanked by partial *GFP* sequences, so that fluorescent Gfp is observed only after recombinative excision of the *URA3*. In contrast, the current pGEM-GFP-URA3-GFP plasmid has a full upstream *GFP* that allows initial transformants to be confirmed visually or spectroscopically prior to 5-FOA selection, and creates a fusion protein (with Gfp comprising the C-terminal moiety) that may also be useful for study; the PCR amplicon used for transfection is also more streamlined, being almost 1 kbp shorter than the one generated from pMG2082.

Gerami-Nejad *et al.* also constructed a plasmid template similar to pMG2082, but with *URA3* replaced by *NAT1*, which confers resistance to nourseothricin [5]; this allows positive selection of transformants, and thus does not rely on *ura3* auxotrophs as hosts. Correct insertion verification requires PCR analysis of the initial transformants; loss of *NAT1* and generation of Gfp fluorescence then requires visual or flow-cytometric screening for identification.

References

1. Cormack BP, Bertram G, Egerton M, Gow NA, Falkow S, Brown AJ. Yeast-enhanced green fluorescent protein (yEGFP): a reporter of gene expression in Candida albicans. Microbiology (Reading, England). 1997;143 (Pt 2):303-11. Epub 1997/02/01. doi: 10.1099/00221287-143-2-303. PubMed PMID: 9043107.
2. Granger BL, Flenniken ML, Davis DA, Mitchell AP, Cutler JE. Yeast wall protein 1 of Candida albicans. Microbiology (Reading, England). 2005;151(Pt 5):1631-44. Epub 2005/05/05. doi: 10.1099/mic.0.27663-0. PubMed PMID: 15870471.
3. Dopf J, Horiagon TM. Deletion mapping of the Aequorea victoria green fluorescent protein. Gene. 1996;173(1 Spec No):39-44. Epub 1996/01/01. PubMed PMID: 8707054.
4. Gerami-Nejad M, Dulmage K, Berman J. Additional cassettes for epitope and fluorescent fusion proteins in Candida albicans. Yeast (Chichester, England). 2009;26(7):399-406. Epub 2009/06/09. doi: 10.1002/yea.1674. PubMed PMID: 19504625; PubMed Central PMCID: PMCPMC3086567.
5. Gerami-Nejad M, Forche A, McClellan M, Berman J. Analysis of protein function in clinical C. albicans isolates. Yeast (Chichester, England). 2012;29(8):303-9. Epub 2012/07/11. doi: 10.1002/yea.2910. PubMed PMID: 22777821; PubMed Central PMCID: PMCPMC3449217.

Full nucleotide sequence of pGEM-GFP-URA3-GFP

(5300 bases; non-vector protein-coding regions are capitalized)

atgcatccaacgcgttgggagctctcccatatggtcgacctgcaggcggccgcactagtgattgttaagctttattaaaATGTCTAAAGGTGA
AGAATTATTCACTGGTGTTGTCCCAATTTTGGTTGAATTAGATGGTGATGTTAATGGTCACAAATTTTC
TGTCTCCGGTGAAGGTGAAGGTGATGCTACTTACGGTAAATTGACCTTAAAATTTATTTGTACTACTG
GTAAATTGCCAGTTCCATGGCCAACCTTAGTCACTACTTTCGGTTATGGTGTTCAATGTTTTGCGAGA
TACCCAGATCATATGAAACAACATGACTTTTTCAAGTCTGCCATGCCAGAAGGTTATGTTCAAGAAAG
AACTATTTTTTTCAAAGATGACGGTAACTACAAGACCAGAGCTGAAGTCAAGTTTGAAGGTGATACCT
TAGTTAATAGAATCGAATTAAAAGGTATTGATTTTAAAGAAGATGGTAACATTTTAGGTCACAAATTGG
AATACAACTATAACTCTCACAATGTTTACATCATGGCTGACAAACAAAAGAATGGTATCAAAGTTAACT
TCAAAATTAGACACAACATTGAAGATGGTTCTGTTCAATTAGCTGACCATTATCAACAAAATACTCCAA
TTGGTGATGGTCCAGTCTTGTTACCAGACAACCATTACTTATCCACTCAATCTGCCTTATCCAAAGAT
CCAAACGAAAAGAGAGACCACATGGTCTTGTTAGAATTTGTTACTGCTGCTGGTATTACCCATGGTAT
GGATGAATTGTACAAATAAgagtaattacaaagtactaatcggaattgatttggatggtataaacggaaacaaaaaaaagagctggta
ctactttctttaaaattattttattatttgattttatttaatagtatatattatattttgaacgtagattattttgttgaaagttgctgtagtgccattgattcgtaacacta

attctgtattagtcattcctcttgtttgatagtatccaaaaaaacggctattttttgcaatcttatttcctgcatattatacagataacataatgaaagaaaag
atcttttttttgttcatcaatgatgatttcaaccattcttttaaacattgatcaattcctgagcaacaaccccatacacactggtttatatatcgccccttttaca
gttgaagaaagaaatagaaatagaaatagcaaacaaaagatATGACAGTCAACACTAAGACCTATAGTGAGAGAGCAG
AAAGGAAAACTCATGCCTCACCAGTAGCACAACGATTATTTCGATTAATGGAACTGAAGAAAACCAAT
TTATGTGCATCAATTGATGTTGATACCACTAAGGAATTCCTTGAATTAATTGATAAATTGGGTCCTTAT
GTATGCTTAATCAAGACTCATATTGATATAATCAATGATTTTTCCTATGAATCCACTATTGAACCATTAT
TAGAACTTTCACGTAAACATCAATTTATGATTTTTGAAGATAGAAAATTTGCTGATATTGGTAATACCG
TGAAGAAACAATATATTGGTGGAGTTTATAAAATTAGTAGTTGGGCAGATATTACTAATGCTCATGGT
GTCACTGGGAATGGAGTAGTTGAAGGATTAAAACAGGGAGCTAAAGAAACCACCACCAACCAAGAG
CCAAGAGGGTTATTGATGTTAGCTGAATTATCATCAGTGGGATCATTAGCATATGGAGAATATTCTCA
AAAAACTGTTGAAATTGCTAAATCCGATAAGGAATTTGTTATTGGATTTATTGCCCAACGTGATATGG
GTGGACAAGAAGAAGGATTTGATTGGCTTATTATGACACCTGGAGTTGGATTAGATGATAAAGGTGA
TGGATTAGGACAACAATATAGAACTGTTGATGAAGTTGTTAGCACTGGAACTGATATTATCATTGTTG
GTAGAGGATTGTTTGGTAAAGGAAGAGATCCAGATATTGAAGGTAAAAGGTATAGAGATGCTGGTTG
GAATGCTTATTTGAAAAAAACTGGCCAATTATAAatgtgaaggggggagattttcactttattagatttgtatatatgtataataaata
aataaataagttaaataaataattaaataagggtggtaattattactatttacaatcaaaggtggtccttctAGACACAACATTGAAGATGG
TTCTGTTCAATTAGCTGACCATTATCAACAAAATACTCCAATTGGTGATGGTCCAGTCTTGTTACCAGA
CAACCATTACTTATCCACTCAATCTGCCTTATCCAAAGATCCAAACGAAAAGAGAGACCACATGGTCT
TGTTAGAATTTGTTACTGCTGCTGGTATTACCCATGGTATGAGTTCTGCTTCCCCATCGGGTTCTGGG
CCCaattcgccctatagtgagtcgtattacaattcactggccgtcgttttacaacgtcgtgactgggaaaaccctggcgttacccaacttaatcgcctt
gcagcacatccccctttcgccagctggcgtaatagcgaagaggcccgcaccgatcgcccttcccaacagttgcgcagcctgaatggcgaatggac
gcgccctgtagcggcgcattaagcgcggcgggtgtggtggttacgcgcagcgtgaccgctacacttgccagcgccctagcgcccgctcctttcgcttt
cttcccttcctttctcgccacgttcgccggctttccccgtcaagctctaaatcgggggctccctttagggttccgatttagtgctttacggcacctcgacccc
aaaaaacttgattagggtgatggttcacgtagtgggccatcgccctgatagacggtttttcgccctttgacgttggagtccacgttctttaatagtggactc
ttgttccaaactggaacaacactcaaccctatctcggtctattcttttgatttataagggattttgccgatttcggcctattggttaaaaaatgagctgatttaa
caaaaatttaacgcgaattttaacaaaatattaacgcttacaatttcctgatgcggtattttctccttacgcatctgtgcggtatttcacaccgcatcaggtg
gcacttttcggggaaatgtgcgcggaacccctatttgtttatttttctaaatacattcaaatatgtatccgctcatgagacaataaccctgataaatgcttca
ataatattgaaaaaggaagagtatgagtattcaacatttccgtgtcgcccttattcccttttttgcggcattttgccttcctgtttttgctcacccagaaacgct
ggtgaaagtaaaagatgctgaagatcagttgggtgcacgagtgggttacatcgaactggatctcaacagcggtaagatccttgagagttttcgcccc
gaagaacgttttccaatgatgagcacttttaaagttctgctatgtggcgcggtattatcccgtattgacgccgggcaagagcaactcggtcgccgcata
cactattctcagaatgacttggttgagtactcaccagtcacagaaaagcatcttacggatggcatgacagtaagagaattatgcagtgctgccataac
catgagtgataacactgcggccaacttacttctgacaacgatcggaggaccgaaggagctaaccgcttttttgcacaacatgggggatcatgtaact
cgccttgatcgttgggaaccggagctgaatgaagccataccaaacgacgagcgtgacaccacgatgcctgtagcaatggcaacaacgttgcgca
aactattaactggcgaactacttactctagcttcccggcaacaattaatagactggatggaggcggataaagttgcaggaccacttctgcgctcggcc
cttccggctggctggtttattgctgataaatctggagccggtgagcgtgggtctcgcggtatcattgcagcactggggccagatggtaagccctcccgt
atcgtagttatctacacgacggggagtcaggcaactatggatgaacgaaatagacagatcgctgagataggtgcctcactgattaagcattggtaa
ctgtcagaccaagtttactcatatatactttagattgatttaaaacttcatttttaatttaaaaggatctaggtgaagatcctttttgataatctcatgaccaaa
atcccttaacgtgagttttcgttccactgagcgtcagaccccgtagaaaagatcaaaggatcttcttgagatcctttttttctgcgcgtaatctgctgcttgc
aaacaaaaaaaccaccgctaccagcggtggtttgtttgccggatcaagagctaccaactctttttccgaaggtaactggcttcagcagagcgcagat
accaaatactgttcttctagtgtagccgtagttaggccaccacttcaagaactctgtagcaccgcctacatacctcgctctgctaatcctgttaccagtgg
ctgctgccagtggcgataagtcgtgtcttaccgggttggactcaagacgatagttaccggataaggcgcagcggtcgggctgaacggggggttcgt
gcacacagcccagcttggagcgaacgacctacaccgaactgagatacctacagcgtgagctatgagaaagcgccacgcttcccgaagggaga
aaggcggacaggtatccggtaagcggcagggtcggaacaggagagcgcacgagggagcttccagggggaaacgcctggtatctttatagtcct
gtcgggtttcgccacctctgacttgagcgtcgatttttgtgatgctcgtcaggggggcggagcctatggaaaaacgccagcaacgcggcctttttacgg
ttcctggccttttgctggccttttgctcacatgttctttcctgcgttatcccctgattctgtggataaccgtattaccgcctttgagtgagctgataccgctcgcc
gcagccgaacgaccgagcgcagcgagtcagtgagcgaggaagcggaagagcgcccaatacgcaaaccgcctctccccgcgcgttggccgat
tcattaatgcagctggcacgacaggtttcccgactggaaagcgggcagtgagcgcaacgcaattaatgtgagttagctcactcattaggcacccca
ggctttacactttatgcttccggctcgtatgttgtgtggaattgtgagcggataacaatttcacacaggaaacagctatgaccatgattacgccaagctat
ttaggtgacactatagaatactcaagct
