# Supplementary figures and images for: Accessibility and contribution to glucan masking of natural and genetically tagged versions of yeast wall protein 1 of Candida albicans
Source: PLoS One. 2018 Jan 12;13(1):e0191194. doi: 10.1371/journal.pone.0191194 (PMC5766240; doi:10.1371/journal.pone.0191194)

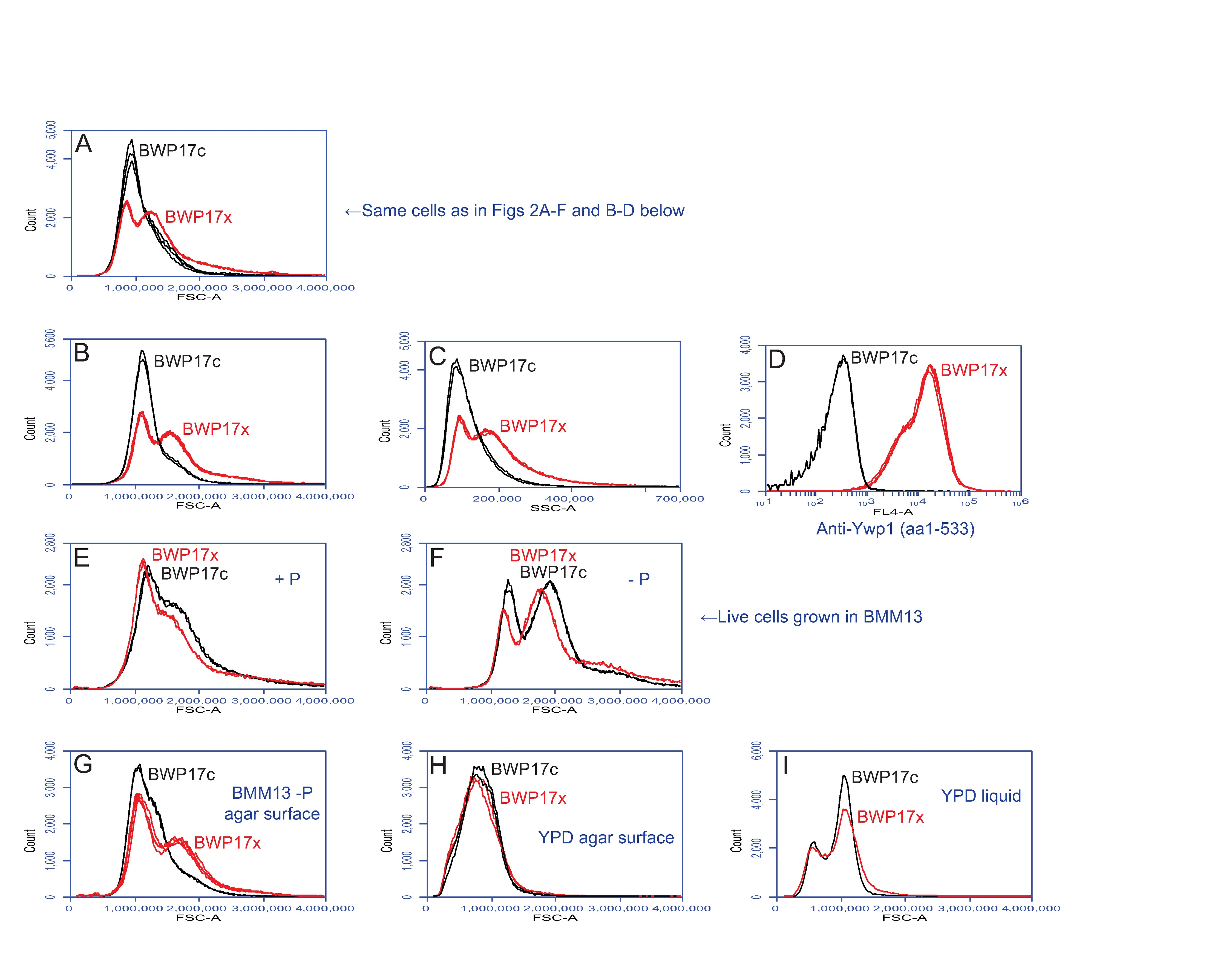

Supplement: S1 Fig — Flow cytometry further revealed that BWP17x under some growth conditions apparently exhibits delayed mother/daughter cell separation relative to BWP17c, resulting in differences in light scattering profiles (S1A Fig). This panel represents the same experiment as shown in Fig 2A, but includes an additional antiserum as well as a control with just secondary antibody. A separate experiment that utilized an alternative secondary antibody and fluorochrome revealed the same patterns (S1B–S1D Fig). Mean and median forward scatter (FSC) values were 1.2–1.3× greater for BWP17x than BWP17c, and side scatter (SSC) values were 1.6–1.7× greater; fluorescence values were 57–64× greater, however, indicating that the per-cell antibody binding differences could not be attributed to differences in cell size or granularity. In other experiments, however, there was often little difference between the light scattering profiles of BWP17c and BWP17x, depending on the medium, phosphate availability, and whether growth was in liquid culture or on solid agar (S1E–S1I Fig). This phenomenon has not yet been investigated systematically, and critical parameters have not yet been discerned for these differences. (TIF) [file pone.0191194.s002.tif]

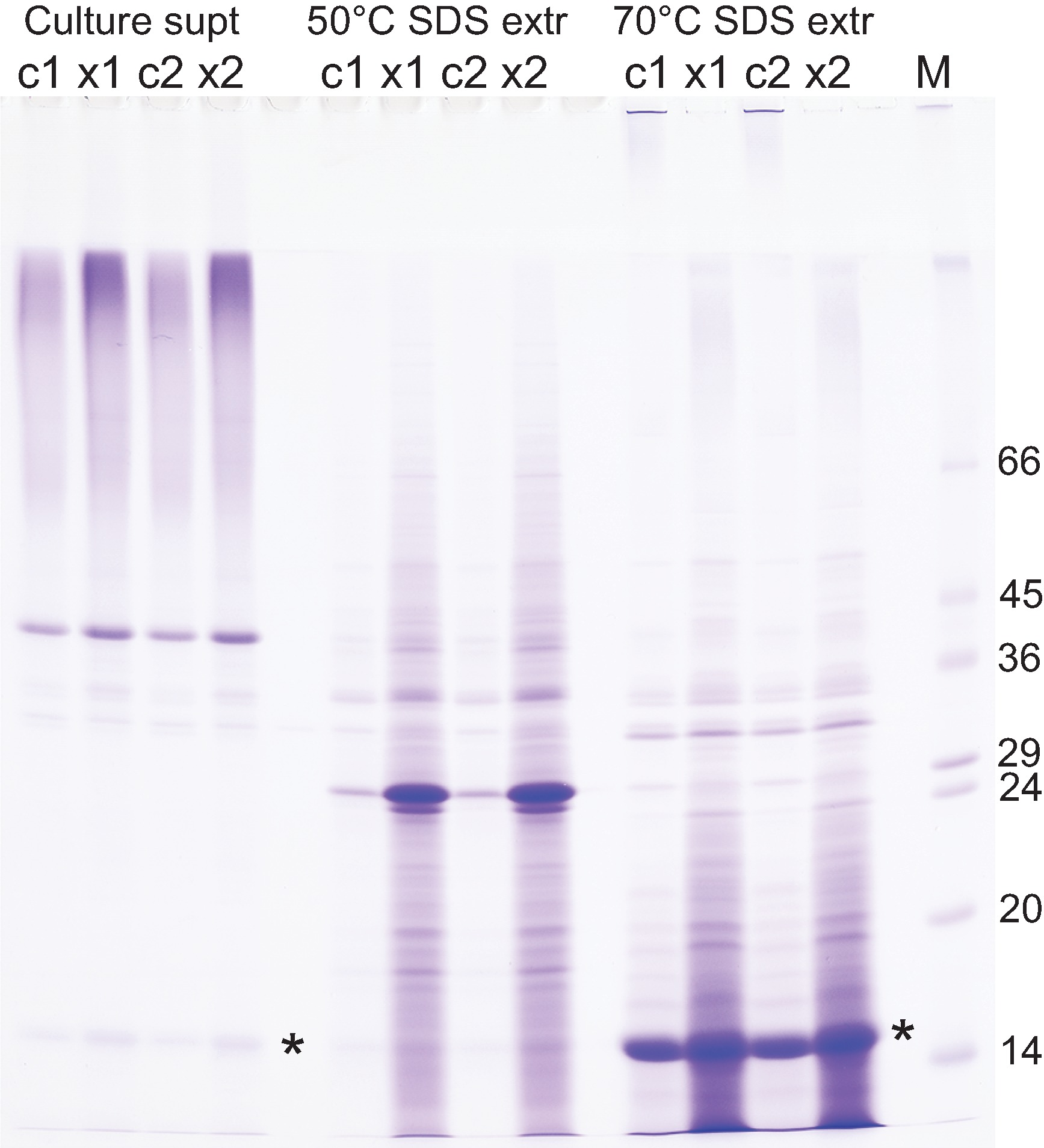

Supplement: S2 Fig — SDS-PAGE followed by protein staining with Coomassie Blue was used to visualize the cleaved, deglycosylated propeptide (*) of Ywp1. Two independent colonies (1 and 2) of BWP17c (“c”) and BWP17x (“x”) were each grown to stationary phase in phosphate-limited BMM13. Culture supernatants, 50°C SDS extracts, and subsequent 70°C SDS extracts were precipitated with ethanol, deglycosylated with PNGase F, and resolved by SDS-PAGE. Each lane represents 2.5 ml of culture. The image includes the stacking gel at the top and marker proteins (M) on the right (with masses shown in kilodaltons). The propeptide quantities and extraction properties appear similar for BWP17c and BWP17x, but more total protein (including the inducible acid phosphatase Pho100 migrating at ~29 kDa) was extracted by SDS from the latter strain, suggesting differences in wall structure or permeability. (TIF) [file pone.0191194.s003.tif]

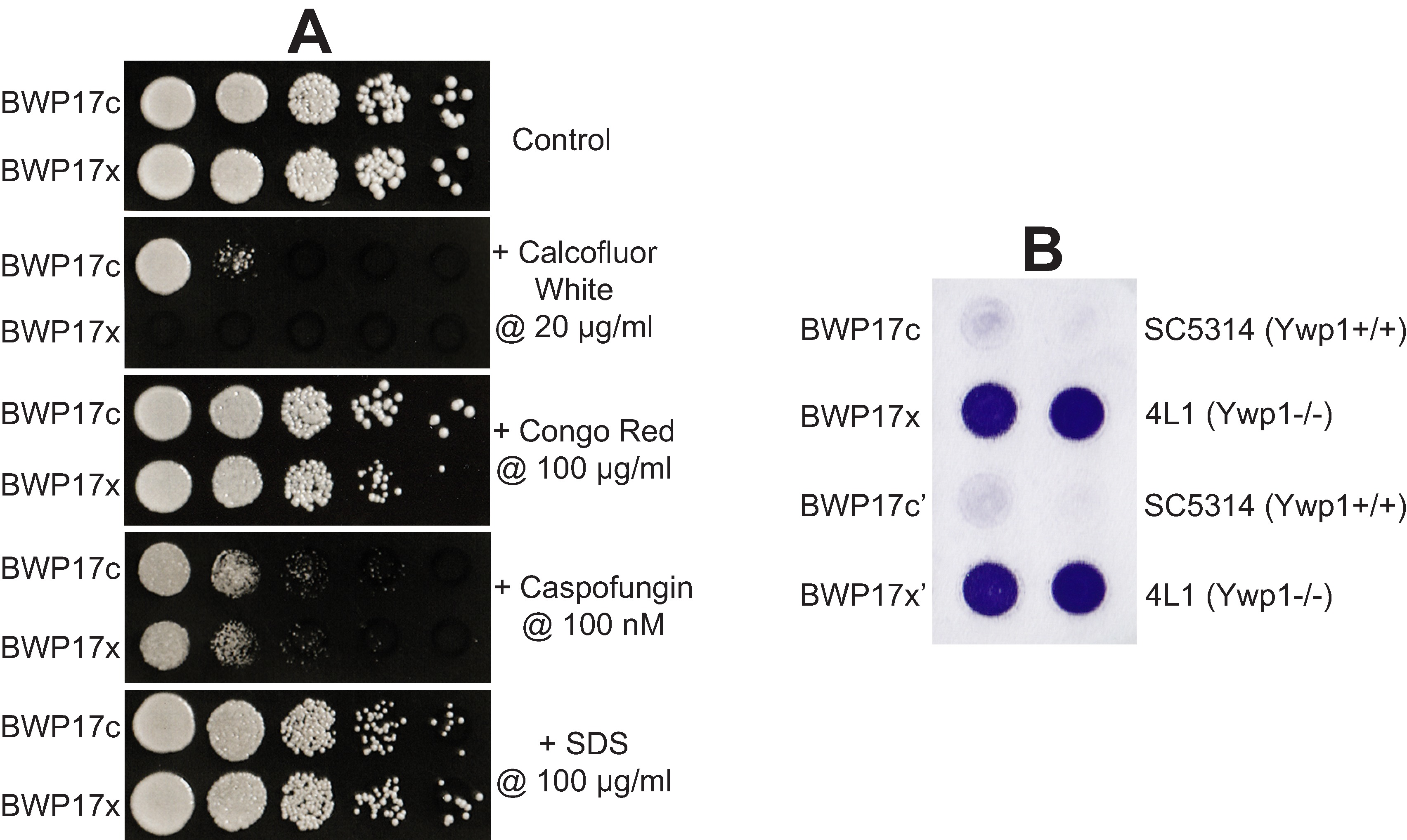

Supplement: S3 Fig — (A) Stationary phase yeast cultures were serially diluted 1/8 (5 times, left to right) and spotted onto YPD agar containing the indicated compounds. The arrays were photographed after 44 hr of growth at 30°C. Sensitivity reduces colony size and/or number. (B) Droplets of dilute yeast microcultures were arrayed on a polystyrene plate and grown to stationary phase in MM13 at 30°C; nonadherent cells were gently rinsed away, and the adherent cells were stained with Crystal Violet [21, 22]. Two independent colonies of BWP17c and two independent colonies of BWP17x were compared; as controls, strain SC5314 (wild type parent of BWP17) and strain 4L1 (Ywp1-negative ywp1::ARG4 / ywp1::URA3) were included in duplicate. (TIF) [file pone.0191194.s004.tif]

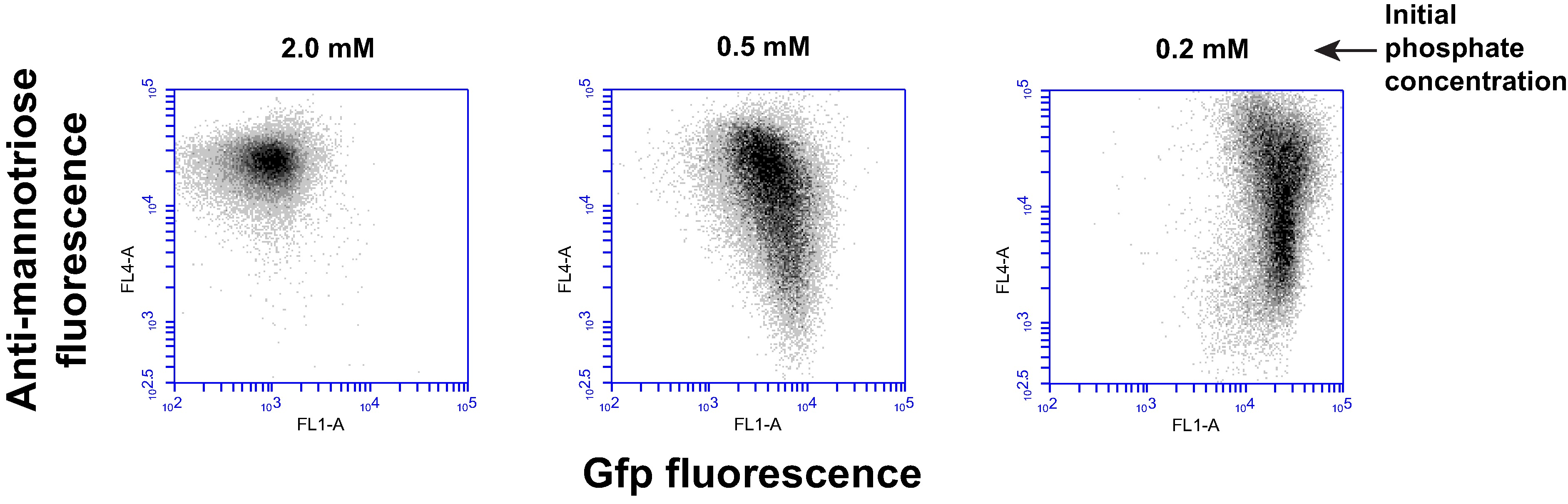

Supplement: S4 Fig — Yeast cells were grown to stationary phase in batch liquid cultures of BMM13 that started with 2.0, 0.5 or 0.2 mM phosphate, as indicated; this resulted in no phosphate starvation (2.0 mM) or phosphate starvation being experienced by a progressively greater proportion of each population. The cells were fixed with formaldehyde, labeled with anti-mannotriose mAb G11.1 followed by a fluorescent red secondary antibody, and analyzed by flow cytometry. Earlier limitation of available phosphate correlated with increased Ywp1-Gfp-Ywp1 accumulation and decreased phosphodiester-linked mannotriose accumulation. (TIF) [file pone.0191194.s005.tif]

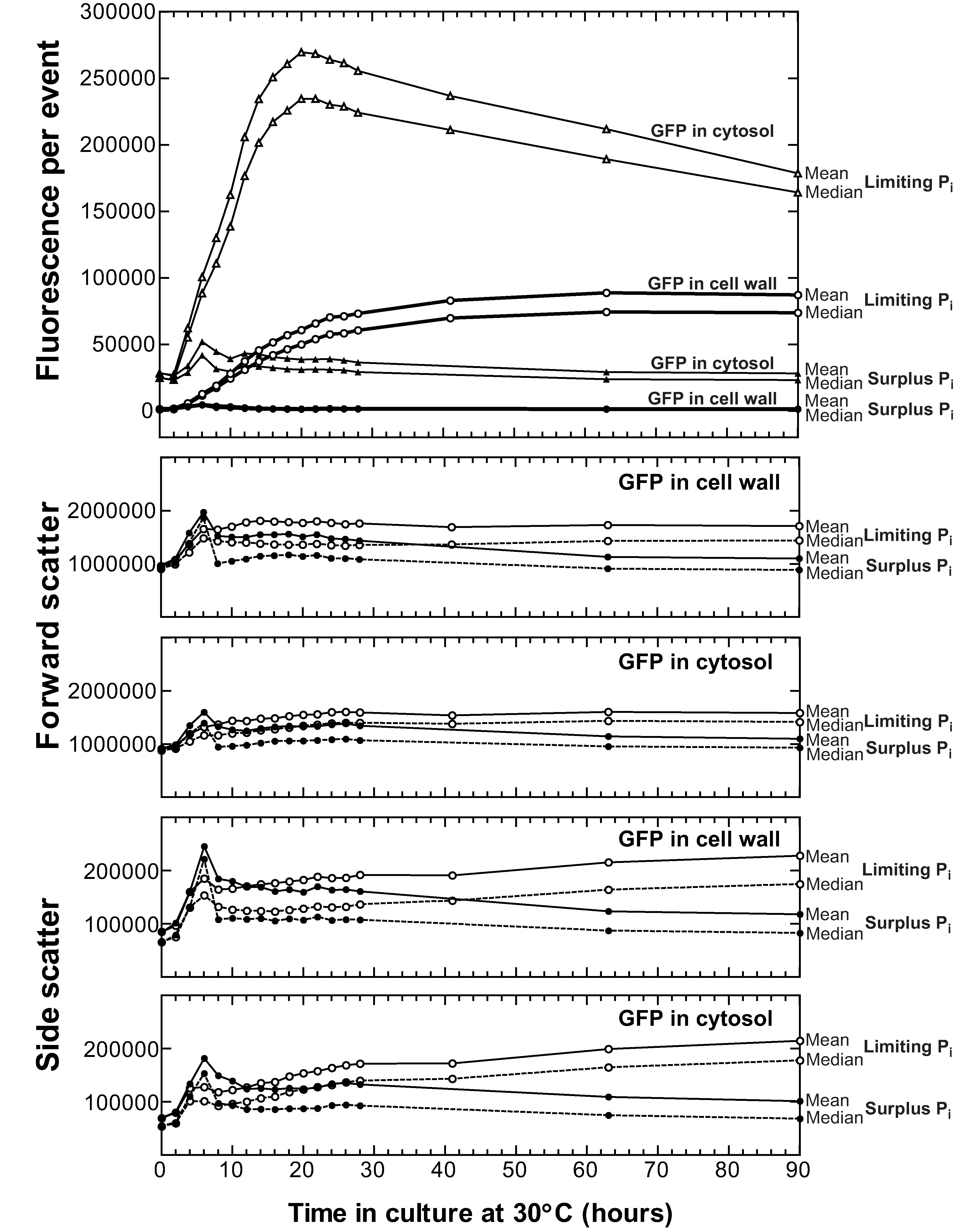

Supplement: S5 Fig — In one strain (BJ4eS8, equivalent to independent transformant BJ3 that was described previously [22]), GFP is a reporter of YWP1 expression and generates soluble Gfp that accumulates in the cytosol; in the other (strain YGY), GFP is inserted into the coding sequence of YWP1, generating a Ywp1-Gfp-Ywp1 glycoprotein that becomes anchored in the cell wall. Each strain has one copy of GFP inserted into one of its two alleles of YWP1. Parallel cultures of yeast forms were grown in BMM13 containing surplus (2.5 mM) or limiting (0 mM) phosphate (Pi). Aliquots of live cells were periodically analyzed for Gfp fluorescence, forward scatter (a function of size), and side scatter (a function of complexity), as shown; the latter two parameters confirm that the increases in fluorescence over time were not attributable to cell aggregation or diminished cell separation. Semi-synchronous growth and budding slightly complicated fluorescence quantitation at early time points in this analysis using ungated samples, as unseparated mother-daughter pairs were measured as single events (as evidenced by the forward scatter and side scatter plots), but in the phosphate-limited cultures the fluorescence rose steadily. A lag period of ~2 hr was followed by yeast growth and budding; first separation of daughter cells from mother cells was prevalent between 4 and 8 hr, but showed less synchrony thereafter. Each point represents the mean or median of 100,000 events; single yeast cells and unseparated mother-daughter doublets are each measured as one event. Phosphate starvation increases YWP1 expression; in the first 24 hr of these cultures, phosphate starvation increased the mean accumulated cytosolic Gfp 7× and the wall-anchored Gfp 40× (relative to phosphate-replete cultures). (TIF) [file pone.0191194.s006.tif]

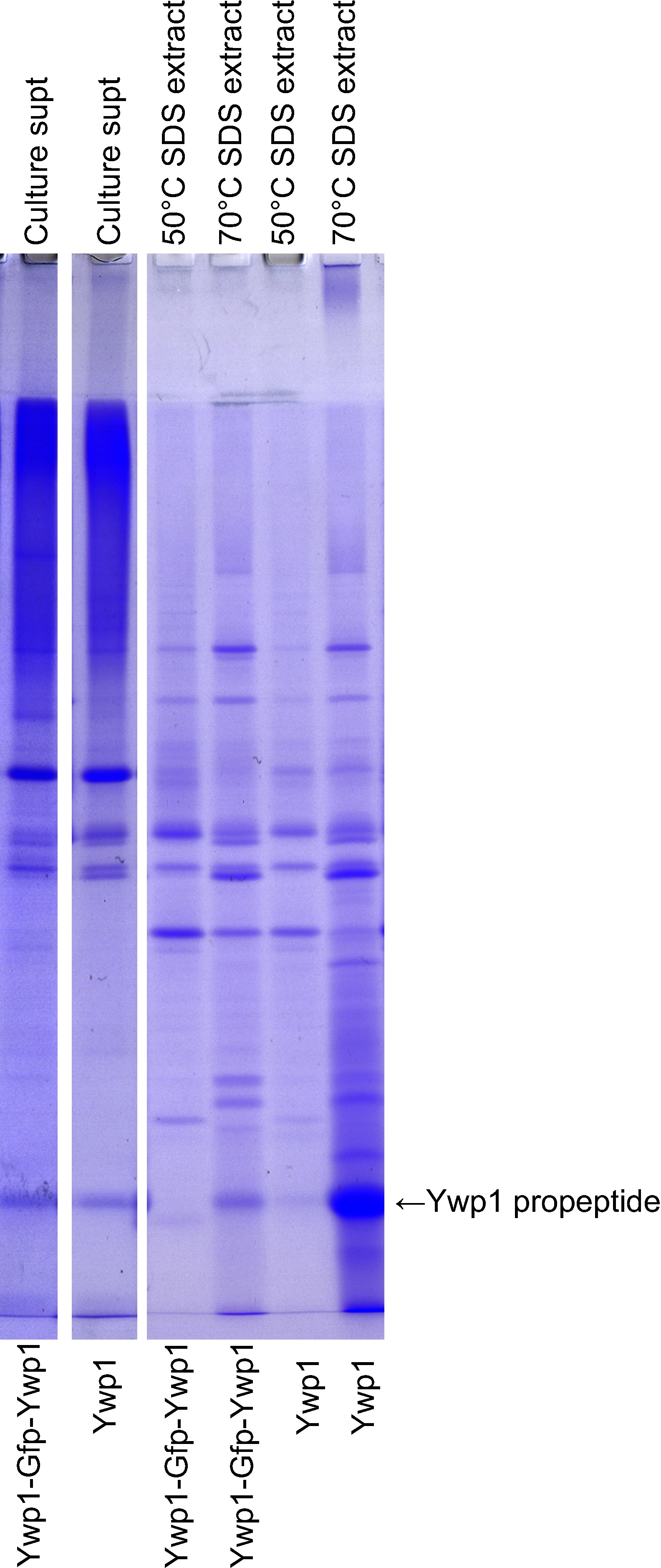

Supplement: S6 Fig — Two derivatives of strain YGY were compared: One with only wild type Ywp1, and the other with only Ywp1-Gfp-Ywp1 (as in Fig 8C and 8D), as indicated below each lane. Samples were prepared as described for S2 Fig. Each lane represents 2.5 ml of stationary phase BMM13 culture that started with 0.2 mM phosphate. The deglycosylated Ywp1 propeptide band is indicated with an arrow. Lanes are from two identical gels run simultaneously in the same apparatus. (TIF) [file pone.0191194.s007.tif]

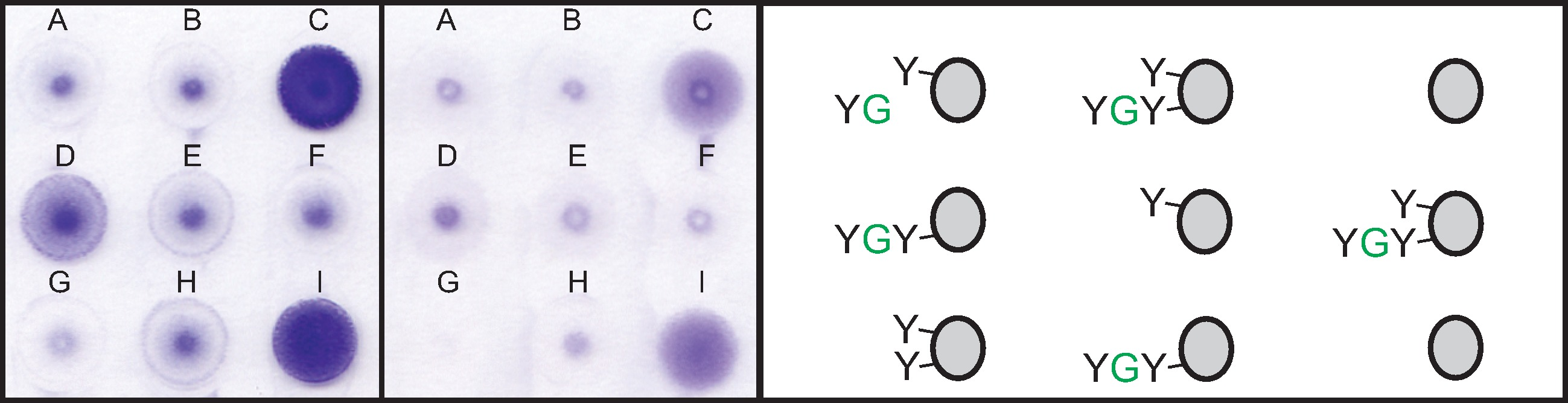

Supplement: S7 Fig — Nine strains were cultured as individual droplets in a polystyrene plate in unbuffered BMM13 containing either 2.5 mM phosphate (left panel) or 0.1 mM phosphate (middle panel). The number of wild type YWP1 alleles per strain is 2 (G), 1 (A, B, E, F) or 0 (C, D, H, I); A secretes Ywp1-Gfp, while B, D, F and H have wall-anchored Ywp1-Gfp-Ywp1. This is shown schematically in the right panel as yeast cells possessing no Ywp1 or wall-anchored Ywp1 (Y-), secreted Ywp1-Gfp (YG), and/or wall-anchored Ywp1-Gfp-Ywp1 (YGY-). Wall-anchored Ywp1-Gfp-Ywp1 thus confers an antiadhesive effect; this effect may be comparable to wild type Ywp1, considering that Ywp1-Gfp-Ywp1 is less abundant in the wall. Detailed description of strains (all derived from BWP17): A Strain YG BWP17 with GFP-URA3 inserted into one allele of YWP1 to encode secreted Ywp1-Gfp B Strain YGY Strain YG (A) that has lost URA3 to encode wall-anchored Ywp1-Gfp-Ywp1 C 4L1 Ywp1-negative double knockout (ywp1::ARG4 / ywp1::URA3) D Strain YGY-Y Strain YGY (B) in which the remaining wild type YWP1 allele was disrupted with URA3 E Strain YGY-G Strain YGY (B) in which the YWP1-GFP-YWP1 allele was disrupted with URA3 F Strain YGY+U Strain YGY (B) in which URA3 allele was inserted outside of either YWP1 locus G DAY185 BWP17 with its auxotrophies restored by insertion of ARG4, URA3 and HIS1 H Strain YGY-Y’ Subclone of Strain YGY-Y (D) with similar intensity of Gfp fluorescence I Strain YGY-Y-G Subclone of Strain YGY-Y (D) with no Gfp fluorescence (spontaneous mutant). (TIF) [file pone.0191194.s008.tif]

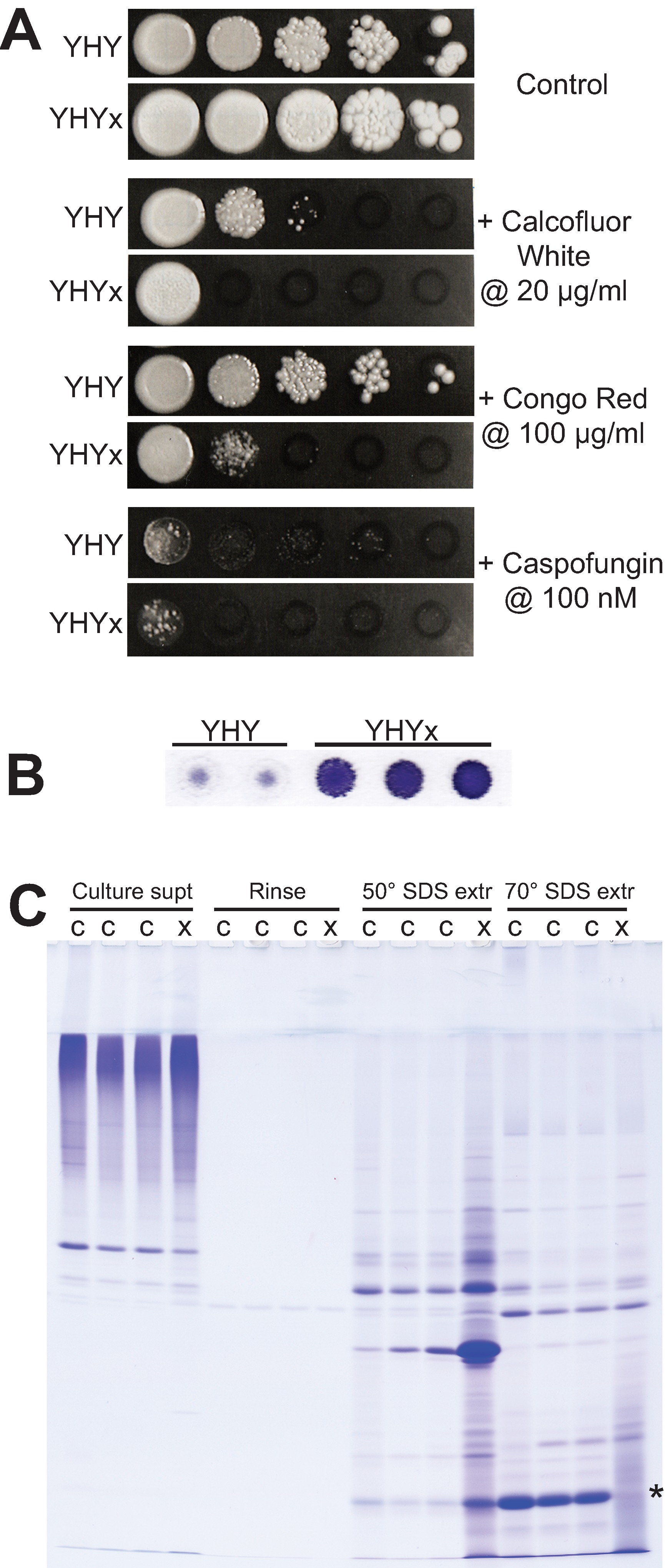

Supplement: S8 Fig — (A) Stationary phase yeast cultures were serially diluted 1/10 (5 times, left to right) and spotted onto YPD agar containing the indicated compounds. The arrays were photographed after 69 hr of growth at 30°C. Sensitivity reduces colony size and/or number. (B) Droplets of dilute yeast microcultures were arrayed on a polystyrene plate and grown to stationary phase in MM13 at 30°C; nonadherent cells were gently rinsed away, and the adherent cells were stained with Crystal Violet. Each spot was grown from one colony picked from a streaked plate during the isolation procedure; genetic analysis identified them as either YHY or YHYx, as indicated, with the latter phenotypically more adhesive, as revealed here. (C) SDS-PAGE followed by protein staining with Coomassie Blue was used to visualize the cleaved, deglycosylated propeptide (*) of Ywp1. Three subpopulations (c; strains YHY) containing less than 0.1%, 0.1% and 10% (respectively, left to right in each set) of high-binding cells were compared to a clonal population (x; strains YHYx) with 100% high-binding cells; each was grown to stationary phase in phosphate-limited BMM13. Culture supernatants, rinses with Tris/EDTA/NaCl at pH 8.1, 50°C SDS extracts, and subsequent 70°C SDS extracts were precipitated with ethanol, deglycosylated with PNGase F, and resolved by SDS-PAGE. The image includes the stacking gel above the resolving gel (as in S2 Fig). (TIF) [file pone.0191194.s009.tif]

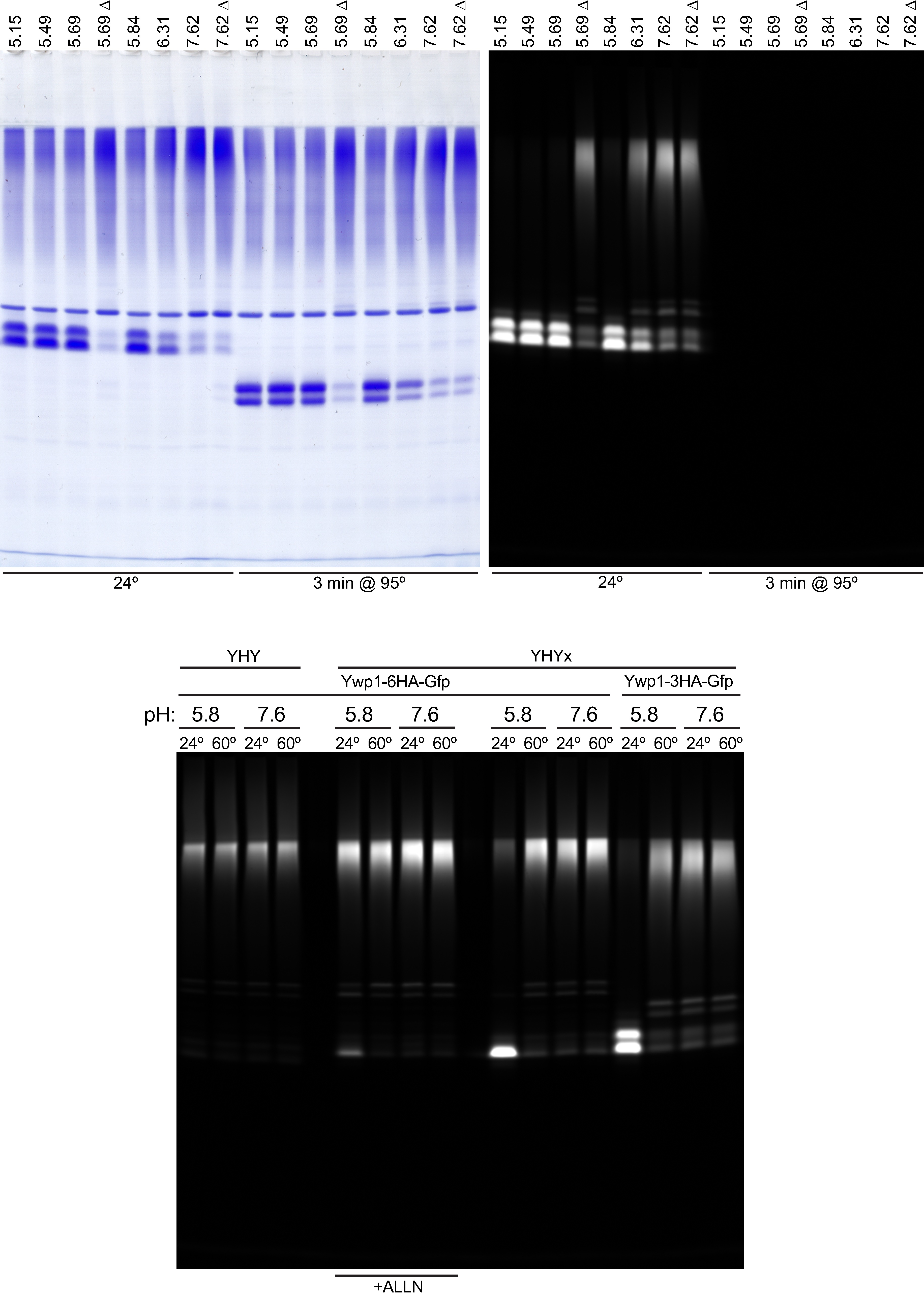

Supplement: S9 Fig — Cells were grown for 45–46 hr in shaking 30°C batch cultures in BMM13 (containing 100 mM MES, 80 mM Bis-Tris and 0.02% Tween-80) that started with 0.3 mM phosphate. Culture supernatants (pH ~5.9) were alkalinized to pH ~8.0 with 240 mM Tris and 9 mM EDTA. For the lower panel, half of each alkalinized supernatant was then heated to 60°C for 30 min; the other half was kept at 24°C, as indicated. All supernatants were then subjected to centrifugal ultrafiltration through a 10K MWCO filter, and the retained proteins were washed with 10 mM Tris / 1 mM CDTA (pH 7.6). Top panel: Ultrafilter retentate containing Ywp1-3HA-Gfp (derived from a strain YHYx parent). Two aliquots were heated to 70°C for 5 min (Δ), and six aliquots were acidified to pH 5.15–6.31 as indicated by adding MES to 167 mM and Bis-Tris to 17–150 mM. All aliquots were incubated 24 hr at 24°C, then mixed with electrophoresis buffer (giving final concentrations of 1% SDS, 20 mM DTT, 200 mM Tris, 100 mM HCl, 2% Ficol-400, 40μM Phenol Red, pH 8.3) and divided in half, with one of each pair heated to 95°C for 3 min (as indicated below the upper panels) before subjecting both to SDS-PAGE. Laser scanning for Gfp fluorescence (right panel) was followed by staining for protein with Coomassie Blue R-250 (left panel). Note that both heating to 70°C and increasing the pH to above 6 each inhibited the activity of endogenous protease(s), diminishing the conversion of the low-mobility smears to sharp mid-gel bands, which consist of fluorescent Gfp but no detectable HA epitopes (as determined by Western blotting). The slight degradation evident in the inhibited samples may have occurred during growth of the culture at pH 5.9. Lower panel: Retentates contained Ywp1-6HA-Gfp or Ywp1-3HA-Gfp (derived from strain YHY or YHYx parents, as indicated). Aliquots of heated (60°C) and unheated (24°C) washed retentates were left at pH 7.6 or acidified to pH 5.8 with 125 mM MES and 50 mM Bis-Tris, then incubated for 19 hr at 24°C befor [file pone.0191194.s010.tif]

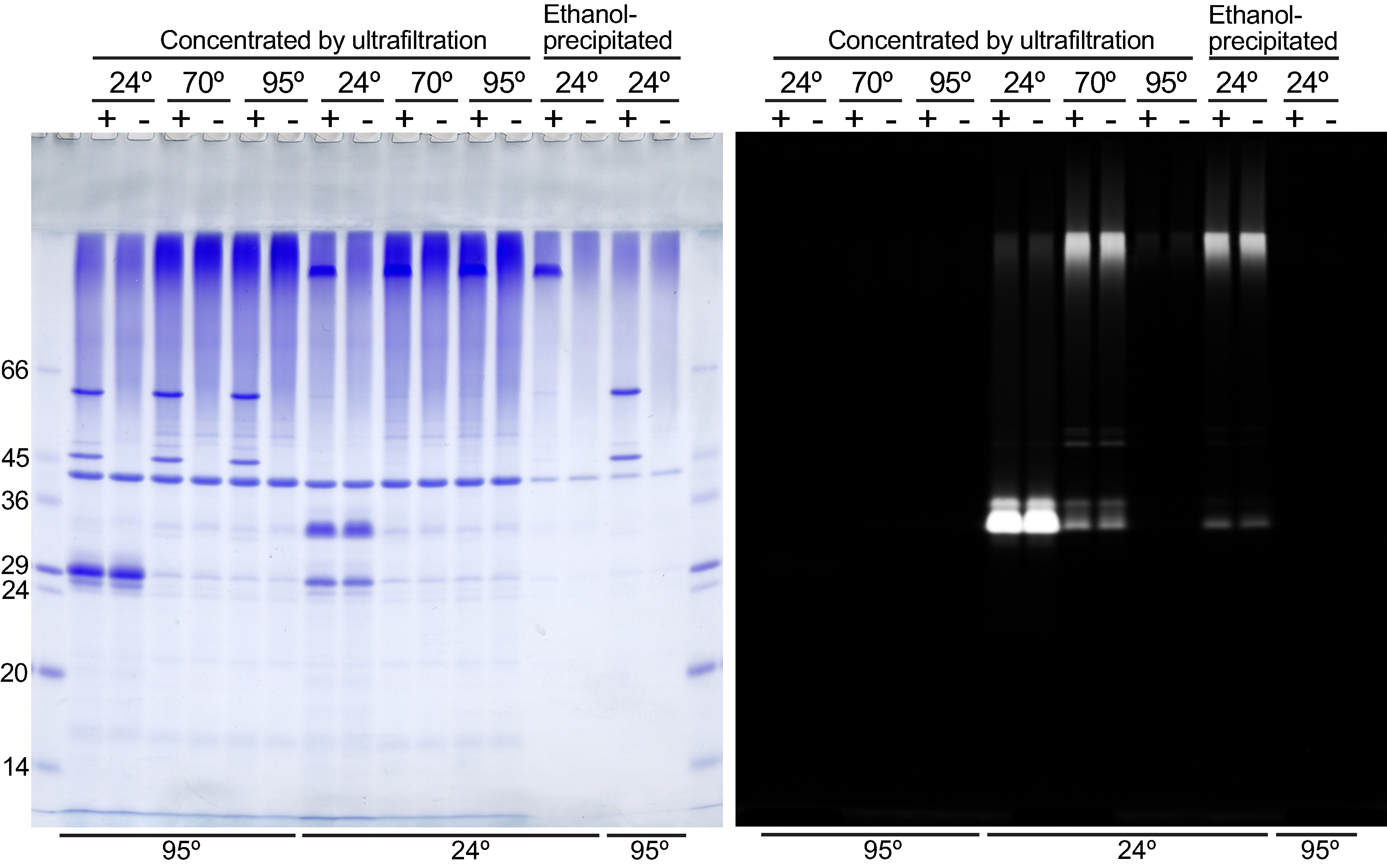

Supplement: S10 Fig — Secreted Ywp1-6HA-Gfp (derived from parental strain YHYx) was concentrated by ultrafiltration (as described for the upper panels in S9 Fig) or by precipitation with an equal volume of ethanol, as indicated. Aliquots of the ultrafiltration retentate were kept at 24°C or heated to inactivate acid proteases (5 min at 70°C, or 3 min at 95°C, as indicated); ethanol precipitation eliminated most of the acid protease activity. Each aliquot was divided in half and incubated for 24 hr at 24°C with (+) or without (-) α(1–2,3,6)-mannosidase in a zinc-containing sodium acetate buffer at pH 5.0 (slightly above the pH optimum of the enzyme, but not low enough to denature the Gfp). Electrophoresis buffer containing SDS and DTT (as described for S9 Fig) was added, and half of each sample was heated to 95°C for 3 min (as indicated below the panels) before SDS-PAGE, fluorescence scanning, and protein staining (as described for S9 Fig). Marker masses (in kilodaltons) are shown on the left. The mannosidase (30 mU per lane) appears as a sharp band near the top of the resolving gel; it converts to smaller (~46 and ~60 kDa) bands upon heating to 95°C in SDS. Control experiments using the chromogenic substrate p-nitrophenyl-α-D-mannopyranoside showed that the mannosidase was highly active in the pH 5 buffer and was not inhibited by anything in the culture supernatant retentate; the retentate also had no mannosidase activity of its own. In conclusion, the mannosidase caused no obvious mobility shift in Ywp1-6HA-Gfp, suggesting that if any O-glycans are present, they are too short or scarce to be responsible for the anomalous migration of undegraded Ywp1-6HA-Gfp. Similar results were obtained for Ywp1-3HA-Gfp. (TIF) [file pone.0191194.s011.tif]

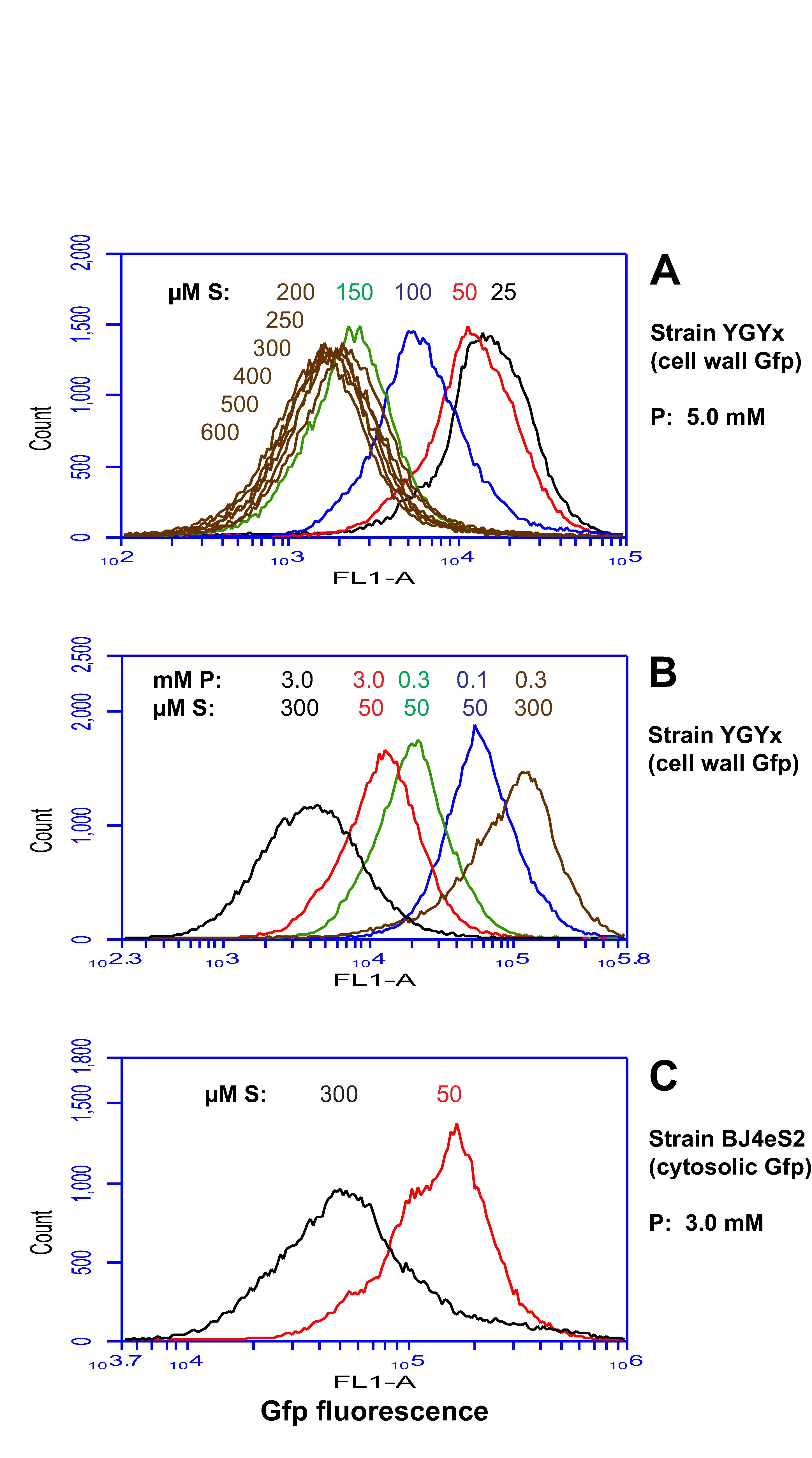

Supplement: S11 Fig — Flow cytometric analyses of YWP1 expression reported by wall-anchored Ywp1-Gfp-Ywp1 (strain YGYx) and cytosolic Gfp (strain BJ4eS2) show induction upon sulfur limitation during growth. Cells were grown in 30°C MM13 with starting concentrations of sulfate (S) and phosphate (P) as indicated (1 mM MgSO4 in MM13 was replaced with 1 mM MgCl2, then ammonium sulfate was added to 25–600 μM; for B and C, 5 mM KH2PO4 was replaced with 5 mM KCl, then KH2PO4 was added to 0.1–3.0 mM). Cultures were buffered with 50 mM phthalic acid and 130 mM (A) or 150 mM (B,C) Bis-Tris, and shaken in flasks for 65 hr (A) or 46–48 hr (B,C) prior to alkalinization to pH 8 for flow cytometry. Mean fluorescence values estimate the YWP1 expression: Panel A suggests that sulfate becomes limiting in MM13 at initial concentrations of less than about 250 μM. In B, under phosphate-replete conditions (3 mM), sulfate limitation (50 μM vs 300 μM) resulted in 2–3× more fluorescence, while under sulfate-replete conditions (300 μM), phosphate limitation (0.3 mM vs 3.0 mM) resulted in about 20× more fluorescence. In A, sulfate limitation (50 μM vs 300 μM) resulted in about 7× more fluorescence, but this ratio may be artificially high because the increased growth of the 300 μM culture dropped the pH to about 0.3 units lower than the 50 μM culture, which may have adversely affected the stability of the external Gfp (especially since these cells were at this pH in stationary phase for 18–20 hr longer than the cells in B, which also started 0.2 pH units higher because of an extra 20 mM Bis-Tris in their medium). Indeed, Gfp that was exposed only to cytosolic pH shows only a doubling of the fluorescence upon sulfate limitation (C). The YWP1 induction upon phosphate limitation thus appears to be about an order of magnitude greater than its induction upon sulfur limitation, as assayed by wall-anchored Gfp; for unknown reasons, cytosolic Gfp (cf. S5 Fig) reports a smaller difference. Note: Elimination of the sulfate f [file pone.0191194.s012.tif]
